# Learning Head and Neck Anatomy Through a Radiological Imaging Platform

**DOI:** 10.15766/mep_2374-8265.11230

**Published:** 2022-03-10

**Authors:** Daniel Hussey, Abigail V. Shaw, Pamela L. Brian, Michelle D. Lazarus

**Affiliations:** 1 Assistant Lecturer, Department of Anatomy and Developmental Biology, Faculty of Medicine, Nursing and Health Sciences, Monash University; 2 Radiologist and Phase I Anatomy Course Co-Director, Pennsylvania State University College of Medicine; 3 Associate Professor and Director, Centre for Human Anatomy Education, and Curriculum Integration Lead, Monash Centre for Scholarship in Health Education (MCSHE), Department of Anatomy and Developmental Biology, Faculty of Medicine, Nursing and Health Sciences, Monash University

**Keywords:** Head, Neck, Imaging, Radiology, Tutorial, Anatomy Review, Gross Anatomy

## Abstract

**Introduction:**

Head and neck anatomy is complex for students to learn and educators to teach. Instructing students on anatomy using radiological imaging can aid comprehension and prepare them for future clinical practice. Computer-aided anatomy instruction is accessible to diverse learners and avoids barriers identified with face-to-face teaching.

**Methods:**

We designed a self-guided PowerPoint tutorial with multiple medical imaging modalities, clinical correlations, and self-review questions incorporated throughout. The tutorial was evaluated with a group of 178 Australian preclinical medical students who had prior teaching related to head and neck anatomy. Student participants were divided into experimental and control groups. Participants completed two knowledge assessments: experimental group before and after tutorial engagement and control group before tutorial engagement. All participants were invited to provide feedback on their experiences with the tutorial via questionnaires.

**Results:**

Engagement with the tutorial improved overall head and neck anatomy knowledge (*p* < .001). Knowledge outcomes were maintained across question group type (e.g., multiple-choice questions, identification, and short-answer questions; *p* < .05), with participants reporting 96% overall positive feedback related to the tutorial experience.

**Discussion:**

Given the improved outcomes following tutorial exposure, our results suggest that this tutorial is efficacious when used in concert with existing anatomy curricula. Participants’ perceived value of the tutorial additionally suggests that it would be taken up well by medical students and is an effective addition to the existing tutorial series. Further research is needed to assess the tutorial's use as a stand-alone addition to the anatomy curriculum.

## Educational Objectives

By the end of this session, users will be able to:

1. Demonstrate basic understanding of the modalities of radiography, computed tomography (CT), and magnetic resonance imaging (MRI).
2. Identify anatomical structures of the head and neck using radiography, CT, and MRI.
3. Demonstrate comprehension of basic head and neck anatomy, as well as relevant relationships.
4. Apply knowledge of head and neck anatomy to clinical scenarios and/or applications.

## Introduction

Across medical curricula, anatomy teaching historically has relied on didactic lectures and laboratory-based learning, including donor dissection, as a means of learning anatomy and structural anatomical relationships.^1^ Modern anatomy teaching, however, increasingly incorporates digital platforms.^2^ Numerous factors have contributed to a move towards computer-aided anatomy instruction, including a shortage of qualified gross anatomy educators, increased student access to technology at both university and home, and, more recently, the barriers of face-to-face teaching related to COVID-19.^1–4^

Students must prepare for the digital world facing them in the clinical health care environment.^1^ Applying learned classroom basic science knowledge (e.g., anatomy) to a patient-focused, professional, working environment can be a daunting step for medical students.^5,6^ Many medical school courses have adopted an integrated course structure to help improve the transfer of preclinical year material by integrating it with knowledge and experience of the clinical environment throughout the duration of the medical program.^7,8^

The use of digital radiological imaging in the teaching of anatomy is one way to ease the integration of traditional preclinical anatomy education with clinical medical imaging.^8^ This approach also allows students to familiarize themselves with medical imaging investigations early in their training, increasing their preparedness for later clinical exposure.^8^ Furthermore, computer-aided anatomy instruction has been shown to enhance learning outcomes.^9^

Previous tutorials in this series have combined a digital anatomy education platform with radiological imaging aimed at novice anatomy learners, predominantly preclinical students who have covered abdominal, thoracic, upper limb, lower limb, and pelvic anatomy.^10–14^ These tutorials can enhance learner outcomes when used as supplementary anatomy instruction.^10–14^

The current project focused on developing a similar tutorial concentrating on the anatomy of the head and neck, a broad anatomical region. Imaging modalities used in this tutorial include computer tomography (CT), magnetic resonance imaging (MRI), and radiographs. CT and MRI are the primary imaging modalities used to evaluate inflammatory, traumatic, and neoplastic diseases of the head and neck according to current guidance.^15^ Radiographs are predominantly used in this region for dental evaluation.^15^ Ultrasound has only limited applications, such as evaluation of the thyroid and soft tissue of the neck, and thus has been excluded from the tutorial.^15^ In this way, the tutorial focuses on areas of medical imaging more readily translated to anatomical relationships: CT, MRI, and radiographs.

Our head and neck tutorial differs from those currently available in a variety of ways. Previously published *MedEdPORTAL* resources cover specific aspects of head and neck anatomy without integrating radiology and focus on narrow areas such as fascial spaces or lymphatics.^16,17^ Some published material does include radiology but again focuses on a limited anatomical region or head and neck topic, such as angiography to teach blood supply of the head and neck, supporting slides for dissection classes, and/or generalized head and neck anatomy atlases.^18–20^ Our tutorial adds to this literature with its holistic representation of head and neck anatomy from a radiological perspective. Our head and neck tutorial enhances interactivity and quizzing opportunities for learners by having image labeling appear prior to naming of anatomical structures, as well as by highlighting clinical correlations and self-evaluation quizzes throughout. This allows for opportunities of spaced learning assessment linked to improved learning outcomes.^21^ Our tutorial utilizes a self-directed modifiable PowerPoint format requiring no tutor input and minimal setup to support the benefits observed with computer-aided anatomy instruction.^9^

We assessed this tutorial as a supplement to the existing anatomy curriculum by evaluating medical students’ performance on a head and neck anatomy knowledge quiz. The assessment included multiple-choice questions (MCQs), image-identification questions, and short-answer questions (SAQs) using an experimental-control methodological design. Our hypothesis was that completion of the tutorial (i.e., experimental group) would improve student learning outcomes in head and neck anatomy, as compared to those who had no access to the tutorial (i.e., control participants).

## Methods

### Tutorial Development

We created a self-directed PowerPoint tutorial covering head and neck anatomy using radiological images (Appendix A). We developed the tutorial through collaboration between an anatomist, a clinical radiologist, a medical resident, and a surgical resident. All developers were working as anatomy educators, in addition to being researchers and clinicians. We chose PowerPoint for the development of the tutorial as this platform tended to be familiar and easy for students and had broad digital device compatibility. Additionally, the platform appeared to be effective based on prior tutorial series evaluations.^10–14^

The head and neck anatomy tutorial began with foundational radiology principles before progressing to an overview of head and neck anatomy, with tutorial progression built around the anatomy and function of the cranial nerves. The anatomical areas of head and neck anatomy covered included the skull, introductory neuroanatomy, major organs (including the eye, ear, nose and sinuses, and salivary glands), and the cervical spine, muscles, fascia, and vessels of the neck. We also included animations and text boxes to help with identification of anatomical structures. We used color-coded text boxes to provide explanations of included anatomy, radiological imaging, clinical correlations, and self-evaluation quizzes. We used radiological images from a deidentified historical imaging database in the tutorial. We designed the tutorial to cover anatomical content that would support existing anatomy curricula (similar to prior tutorial evaluations), and thus, the tutorial was meant to serve as an adjunct to an existing anatomy syllabus, rather than as stand-alone teaching material. The tutorial was written in comprehensible language and was formatted to be easily edited, allowing user engagement as self-directed learning, facilitator-led learning, or small-/large-group learning.^10–14^

### Tutorial Implementation

We recruited second-year medical students via email and obtained written consent from them prior to their participation in the tutorial. Similar to other tutorial evaluations, all students had recently completed their head and neck anatomy curriculum at the time of our tutorial evaluation as an adjunct to anatomy curricula.^10–14^ This curriculum targeted consensus head and neck anatomy knowledge required for general practitioners/primary care providers.^22^ Participation in the tutorial was voluntary and had no bearing on academic progress. Students were asked to bring their own digital device to complete the tutorial.

We randomly assigned student participants to either the control group or the experimental group in two large classrooms. As in previous tutorial evaluations, students in the control group had 1 hour to answer the pretest quiz questions (Appendix B) and then 1 hour to answer the posttest questions (Appendix C).^10–14^ After the control group members completed both quizzes, they left the classroom, and we emailed the head and neck anatomy tutorial to them to access at their own will. Students in the experimental group were given 1 hour to complete the pretest quiz (Appendix B); then, we emailed the head and neck tutorial to them, allowing 1 hour for tutorial review in the classroom. Experimental participants followed tutorial review with a posttest quiz (Appendix C) for 1 hour.

All quizzes were administered on paper and included radiological image-based questions. Tutorial use was self-directed, and the tutorial was viewed on participants’ own individual electronic devices. We invigilated the quizzes to prohibit collusion between students. We provided no didactic teaching or direction to either group during any stage of the evaluation. All results were anonymized and aggregated prior to analysis.

### Tutorial Evaluation

We compiled the pretest and posttest (Appendices B and C) quiz questions using a mixture of MCQs, image-identification questions, and SAQs. The quizzes focused on similar anatomical areas and concepts, including questions on anatomical knowledge and applied anatomy to clinical scenarios. In both tests, we included direct questions (e.g., answers specifically mentioned by the PowerPoint tutorial) and indirect questions (e.g., answers had to be inferred from knowledge taught in the tutorial). The same assessor (Daniel Hussey) marked all tests using an answer guide (Appendices D and E).

After the quiz, we gave all participants online access to the tutorial PowerPoint, the quiz questions (Appendices B and C), and accompanying answers (Appendices D and E). We also emailed all participants, asking them to complete an online feedback questionnaire (Appendix F) including Likert-scale and open-ended questions about their experiences with the tutorial. The questionnaire remained online for 2 weeks.

To analyze the open-ended comments, simple thematic content analysis was used on the manifest content. Michelle D. Lazarus led this analysis by reviewing all comments several times over, from which codes and themes were identified, as described previously.^10–14^ Codes were reviewed by the lead author (Daniel Hussey), with consensus reached.

### Statistical Analysis

We performed a two-tailed power analysis prior to testing. Based on the effect of previous tutorials in the series, an effect size was identified as 0.8, with a significance level of .05.^10–14^ A total of 183 students voluntarily agreed to participate in the evaluation exercise, meaning that 94 students were randomly allocated to the experimental group and 89 to the control group. The post hoc calculated study power was 0.898, suggesting a negligible likelihood for type II error.

We compared the results from the pretest and posttest quiz questions between the experimental and control groups using percentage change and a two-tailed paired *t* test. We conducted the analysis for direct versus indirect questions and question type (MCQ, image identification, and SAQ).

## Results

Voluntary recruitment resulted in a total of 183 second-year medical students being randomly assigned to the control group (*n* = 89) or experimental group (*n* = 94). Four participants from the control group and one participant from the experimental group did not complete both the pretest and posttest and were excluded from the evaluation.

The experimental group performed significantly better than control participants on the posttest by an average of 12% (*t* = 5.37, *p* < .001). Pretest results appeared slightly in favor of the experimental group, whose results were on average 1% higher, but this was not significant (*t* = 0.35, *p* = .73).

When we compared question content (directly from the tutorial vs. indirect tutorial knowledge) across the two groups, the experimental group outperformed the control group on direct questions by an average of 15% (*t* = 6.31, *p* < .001). For indirect questions, the experimental group was again significantly higher than control participants by 6% (*t* = 2.01, *p* = .04).

When we compared the two groups’ assessment performance in the pretest by question type, we found no statistically significant differences for any question type. However, when evaluating posttest performance, we identified a significant improvement of the experimental group in answering MCQs (*t* = 3.94, *p* < .001), image-identification questions (*t* = 5.21, *p* < .001), and SAQs (*t* = 2.93, *p* = .003). Table 1 shows the percentage change overall in the pretest and posttest scores between the control and experimental groups in MCQs, SAQs, image identification, and indirect and direct questions.

**Table 1.**
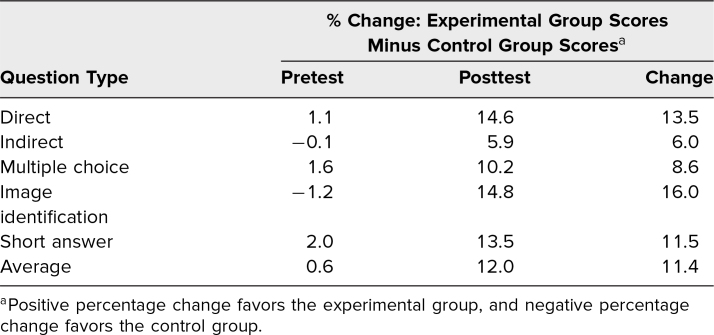
Percentage Change Comparing the Experimental Group Quiz Scores Minus the Control Group Quiz Scores

The tutorial evaluation questionnaire was completed by 48 students (28 female, 20 male) from both groups, providing a 27% response rate. This response rate was similar to that for other online surveys.^23^ The average age of respondents was 19.9 years, with 60% of students from metropolitan areas, 17% international, and 13% from rural or remote areas.

Five-point Likert scales were used for the students’ subjective assessment of the tutorial. Almost all students found the tutorial to be excellent (*n* = 24, 50%) or somewhat good (*n* = 22, 46%) overall, with a single student (2%) finding the tutorial not good. Most students felt the tutorial was effective (48%) or very effective (38%) at helping them revise head and neck anatomy. The majority (52%) felt they had adequate time for the tutorial with the 1-hour time limit provided. Around a third felt they required more time, with the remainder reporting needing less time with the tutorial.

The full set of responses pertaining to students’ confidence in medical imaging and preparedness for clinical placement is detailed in Table 2.

**Table 2.**
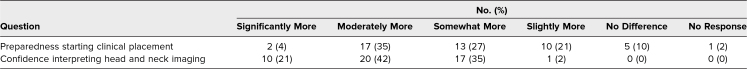
Students’ Responses in the Feedback Questionnaire After Completing the Tutorial

In addition to rating the tutorial, a series of open-ended questions was also posed. Thematic analysis of participant written responses is described below.

In response to “How (if at all) the tutorial has changed your perception of head and neck anatomy?”, two themes were identified: (1) improved awareness of anatomical relevance and (2) altered perceptions of head and neck anatomy. Students reported that “outlining the relevant anatomy helped a lot” and that “seeing outlines and highlighted regions [was] very useful” under the theme of improved awareness of anatomical relevance. With regard to altered perceptions of head and neck anatomy, many students reported that “it is not as hard as I thought it would be,” but others reported challenges such as “it is much more interesting and complicated than I thought.”

In response to “What was most effective in the tutorial and why?”, two themes were identified: (1) tutorial format and (2) clinical relevance. Within tutorial format, students described how the quizzes helped them learn, such as “questions [helped] consolidate knowledge.” Additionally, students appreciated the “ability to test [them]selves” as being helpful to identify “where the gaps in knowledge were.” Under the theme of clinical relevance, students reported that “approaching anatomy in terms of pathology… makes recall easier.”

Overall, thematic analysis supported the quantitative findings that this tutorial was a valued addition to student learning.

## Discussion

Our head and neck anatomy tutorial utilizing radiological imaging extends the existing series available in *MedEdPORTAL* by focusing on a novel area of anatomy. It adds to the existing *MedEdPORTAL* resources focused on head and neck anatomy by combining an introduction to radiological principles, familiarization with different imaging modality, and clinical correlations across a broad area of head and neck anatomy. Our head and neck tutorial is freely available, and its PowerPoint format enables easy access by students from any device.

Similar to previous tutorials in this series, tutorial exposure significantly improved learners’ regional anatomy knowledge.^10–14^ The participants’ applied anatomical knowledge (indirect knowledge that was not explicitly covered in the tutorial) also significantly improved, albeit less so than their performance on direct material covered in the tutorial. This performance difference may suggest a longer exposure time is needed for improvements of indirect knowledge or may indicate a limitation of this tutorial in preparing students for application of head and neck knowledge as compared to other regions.^10–14^ Regardless, knowledge measurably improved in the experimental group, suggesting the utility of this addition to the anatomical radiology series. Compared to previous tutorials in our series, however, the improvement in posttest scores was much smaller with this tutorial: on average, 12%, compared to 14%-21% for pelvic anatomy and 16%-32% for abdominal anatomy.^13,14^ This may reflect the fact that students often find head and neck anatomy the most difficult anatomical region to learn due to the reported complexity of the area, increasing time restraints within curricula, and intrinsic neurophobia engrained in medical students.^24^

Survey feedback indicated that students generally found the tutorial improved their confidence in medical imaging and preparedness for clinical placement. This is in line with previous research highlighting that the provision of clinically oriented education to undergraduate medical students increases their perception of preparedness for their venture into clinical clerkship and that clinical radiology exposure early in medical students training positively impacts their perception of the specialty and interest in pursuing radiology careers.^6,25^ Our tutorial appears to impact students in a similar manner.

Head and neck anatomy is an expansive topic to cover in a tutorial delivered in only a 1-hour session, and this was the first difficulty we encountered when designing the tutorial. We aimed to cover the key areas and had to be selective when choosing what material to include.^22^ Around half the students felt that the allocated time was sufficient, with around a third feeling as though more time would be useful. Previous work suggests that increased time facilitating anatomical education assists with the consolidation of knowledge; thus, future users may want to allot additional time to reviewing and engaging in our tutorial.^21^

Our tutorial was initially created for undergraduate preclinical Australian medical students who had just completed their core head and neck anatomy teaching. The PowerPoint format allows others to adapt this tutorial to their own learning contexts. Educators can selectively choose to enhance or refocus the depth of knowledge covered in the existing tutorial by adding or removing slides. For example, the initial slides covering principles of radiology could be expanded upon to focus the tutorial more on radiology, or regional topics could be split into shorter sessions to allow full concentration and processing of the material.

Additional areas of flexibility in tutorial execution exist in the delivery. Though not directly compared in this evaluation, the tutorial can be delivered either in a group tutorial setting or on the learners’ own time. In a group setting, the facilitator could guide progression of slides via a projector at the front. Alternatively, students could access the tutorial on their own digital devices in a tutorial room with a facilitator addressing specific aspects using available anatomical specimens or models to support the tutorial. Finally, the tutorial could be accessed individually by students on their own devices at any time for self-directed learning, without requiring a facilitator.

Despite the improvements observed with tutorial use, our work is not without limitations. Similar to other student research, because participation in this tutorial was opt-in, there may have been selection bias resulting in only enthusiastic students and/or those struggling with the content having participated.^26^ The analysis of the student performance in pretest and posttest quizzes did not take into consideration the students’ overall academic performance. Without the ability to stratify participants based on overall academic performance, we are unable to identify the impact of this tutorial on different groups of students. The tutorial evaluation was carried out on preclinical medical students due to the research question, and thus, we are unable to draw conclusions about the impact of the tutorial on clinical year medical students, qualified medical practitioners, or other types of student groups.

Despite these limitations, both the quantitative and subjective assessment data strongly suggest that the tutorial is successful for consolidation of knowledge of head and neck anatomy that students would be likely to require. Learning anatomy via engagement with imaging modalities not only helps students during clinical years but also improves their understanding of the three-dimensional spatial relationship of anatomical structures; this is especially important for future careers in clinical specialties such as clinical radiology and surgery.^27^ Further work could assess the tutorial's function as a stand-alone addition to an anatomy curriculum or for novice learners with no previous experience of this area of anatomy. A more robust measure of the tutorial's effectiveness could involve assessing long-term knowledge retention and whether the knowledge gained translates into improvement in knowledge and understanding in the clinical environment.

## Appendices


Head and Neck Imaging Tutorial.pptxPretest.docxPosttest.docxPretest Answers.docxPosttest Answers.docxHead and Neck Tutorial Survey.docx
*All appendices are peer reviewed as integral parts of the Original Publication.*
