# Supplementary material for: Learning Head and Neck Anatomy Through a Radiological Imaging Platform
Source: MedEdPORTAL. 2022 Mar 10;18:11230. doi: 10.15766/mep_2374-8265.11230 (PMC8907321; doi:10.15766/mep_2374-8265.11230)
Supplement: Supplementary file 1 — Head and Neck Imaging Tutorial.pptxPretest.docxPosttest.docxPretest Answers.docxPosttest Answers.docxHead and Neck Tutorial Survey.docx [file mep_2374-8265.11230-s001.zip › A. Head and Neck Imaging Tutorial.pptx]

## Slide 1
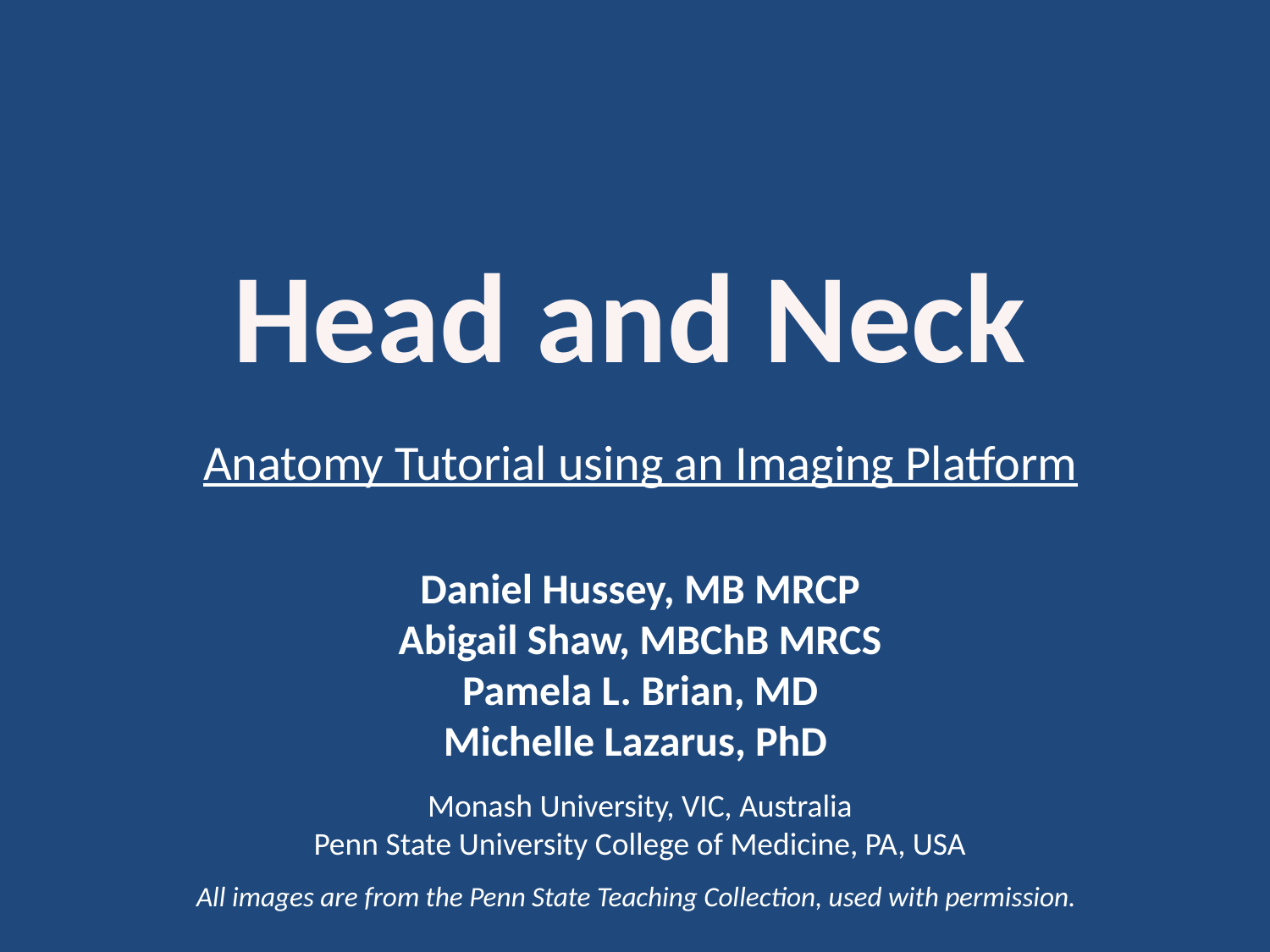

Head and Neck
Anatomy Tutorial using an Imaging Platform
# Daniel Hussey, MB MRCP Abigail Shaw, MBChB MRCS
Pamela L. Brian, MD
Michelle Lazarus, PhD
Monash University, VIC, Australia
Penn State University College of Medicine, PA, USA
All images are from the Penn State Teaching Collection, used with permission.

## Slide 2
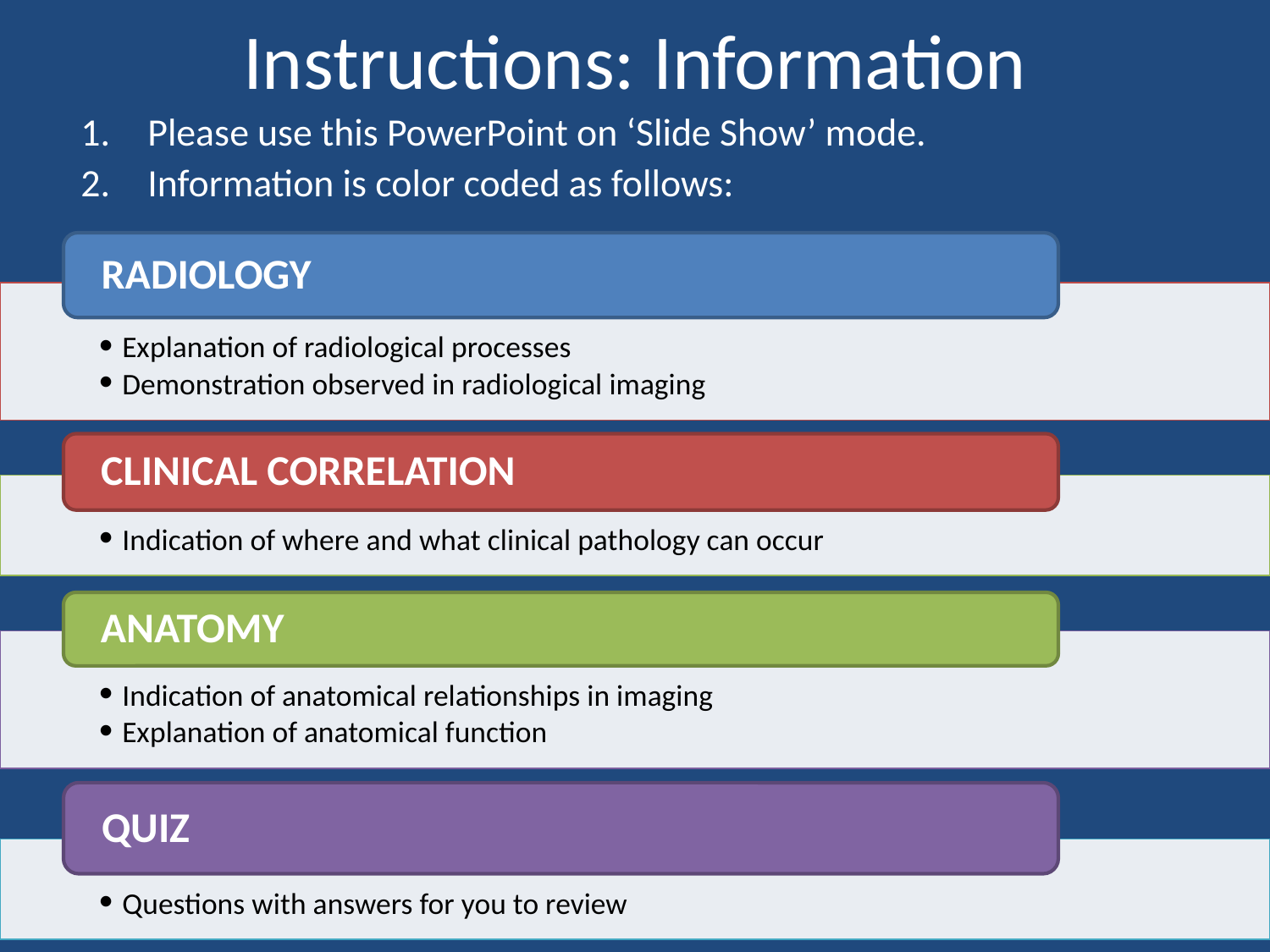

# Instructions: Information
Please use this PowerPoint on ‘Slide Show’ mode.
Information is color coded as follows:
RADIOLOGY
Explanation of radiological processes
Demonstration observed in radiological imaging
CLINICAL CORRELATION
Indication of where and what clinical pathology can occur
ANATOMY
Indication of anatomical relationships in imaging
Explanation of anatomical function
QUIZ
Questions with answers for you to review

## Slide 3
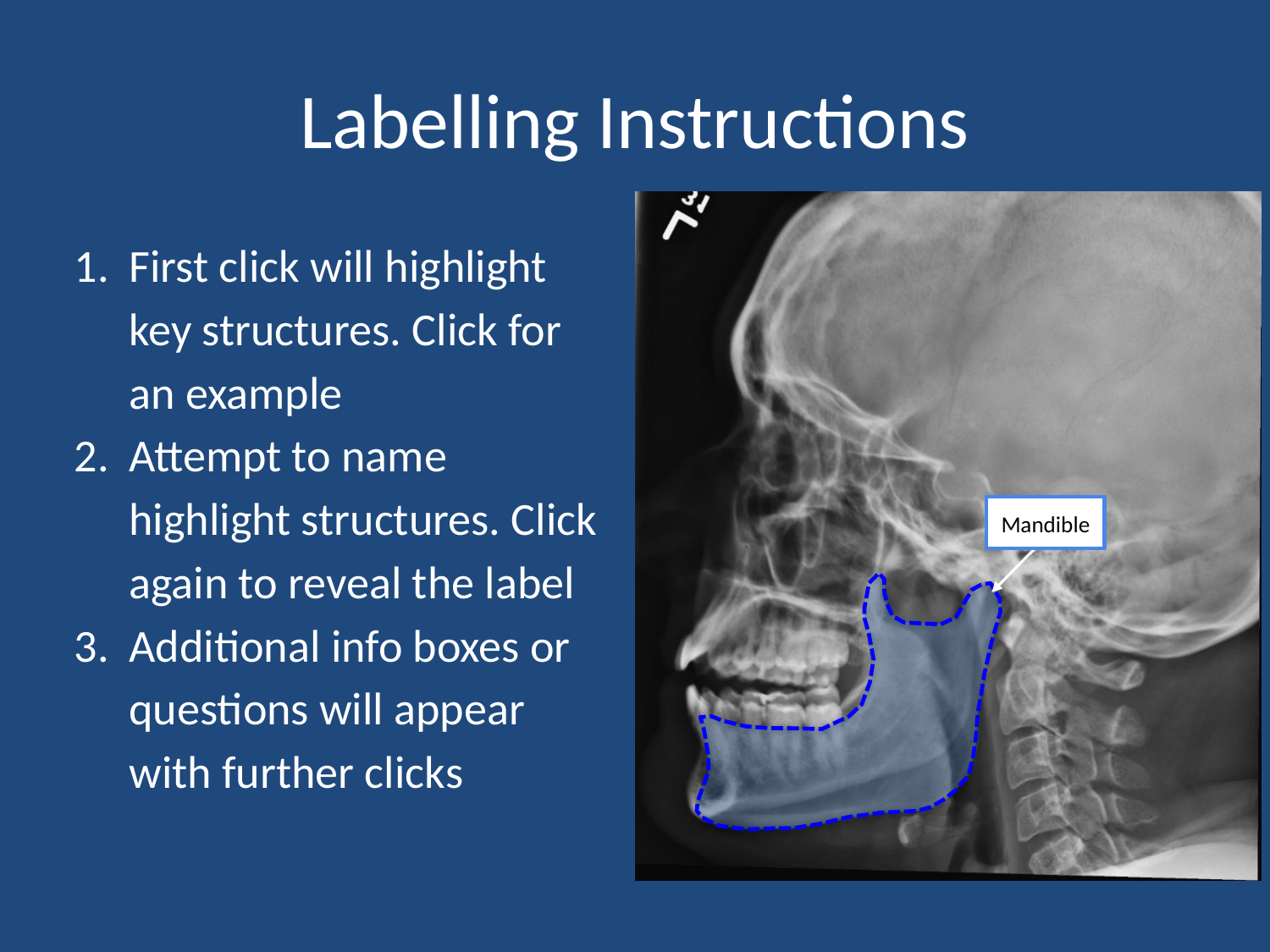

# Labelling Instructions
First click will highlight key structures. Click for an example
Attempt to name highlight structures. Click again to reveal the label
Additional info boxes or questions will appear with further clicks
Mandible

## Slide 4
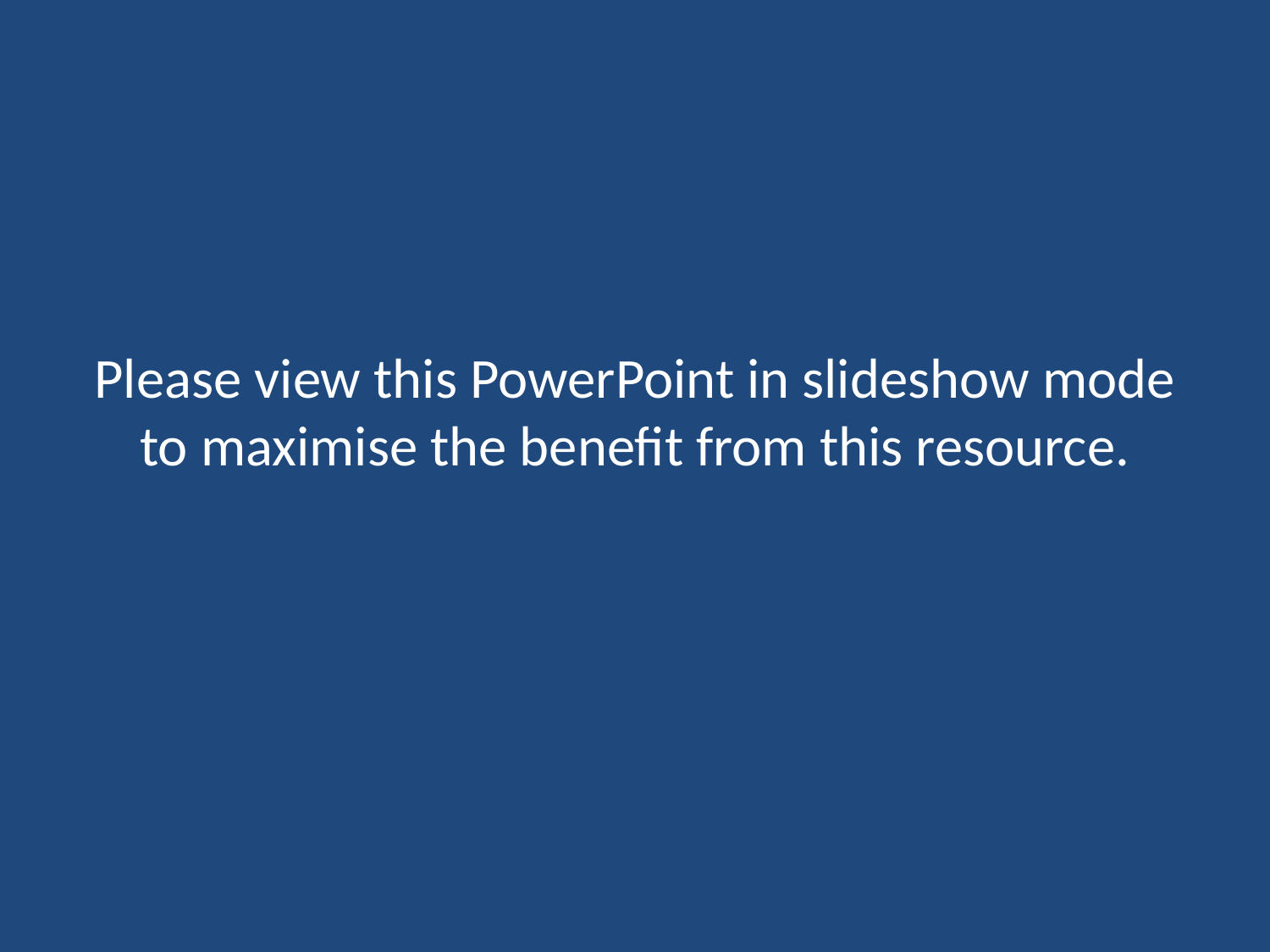

#
Please view this PowerPoint in slideshow mode to maximise the benefit from this resource.

## Slide 5
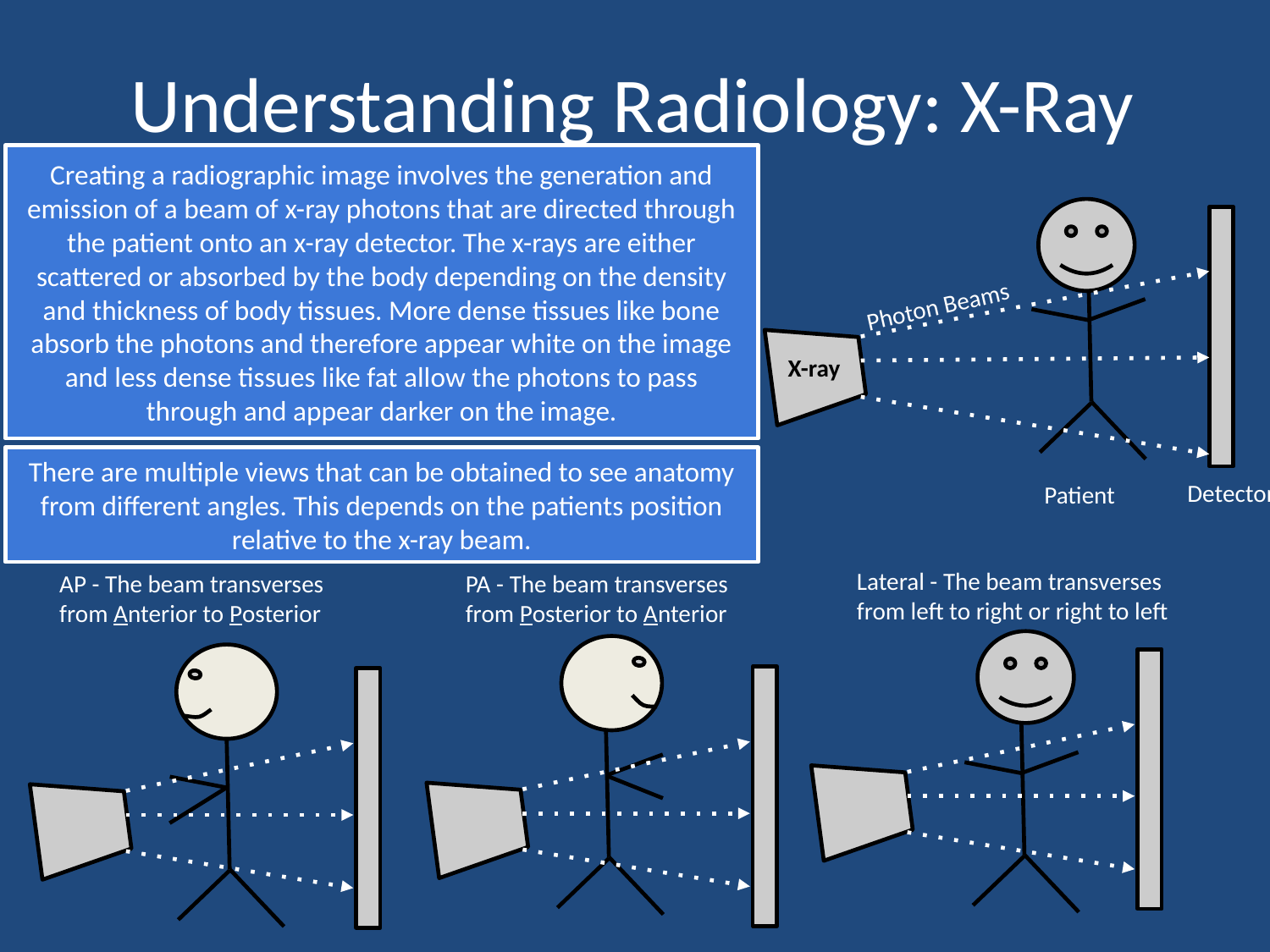

# Understanding Radiology: X-Ray
Creating a radiographic image involves the generation and emission of a beam of x-ray photons that are directed through the patient onto an x-ray detector. The x-rays are either scattered or absorbed by the body depending on the density and thickness of body tissues. More dense tissues like bone absorb the photons and therefore appear white on the image and less dense tissues like fat allow the photons to pass through and appear darker on the image.
Photon Beams
X-ray
There are multiple views that can be obtained to see anatomy from different angles. This depends on the patients position relative to the x-ray beam.
Detector
Patient
Lateral - The beam transverses from left to right or right to left
AP - The beam transverses from Anterior to Posterior
PA - The beam transverses from Posterior to Anterior

## Slide 6
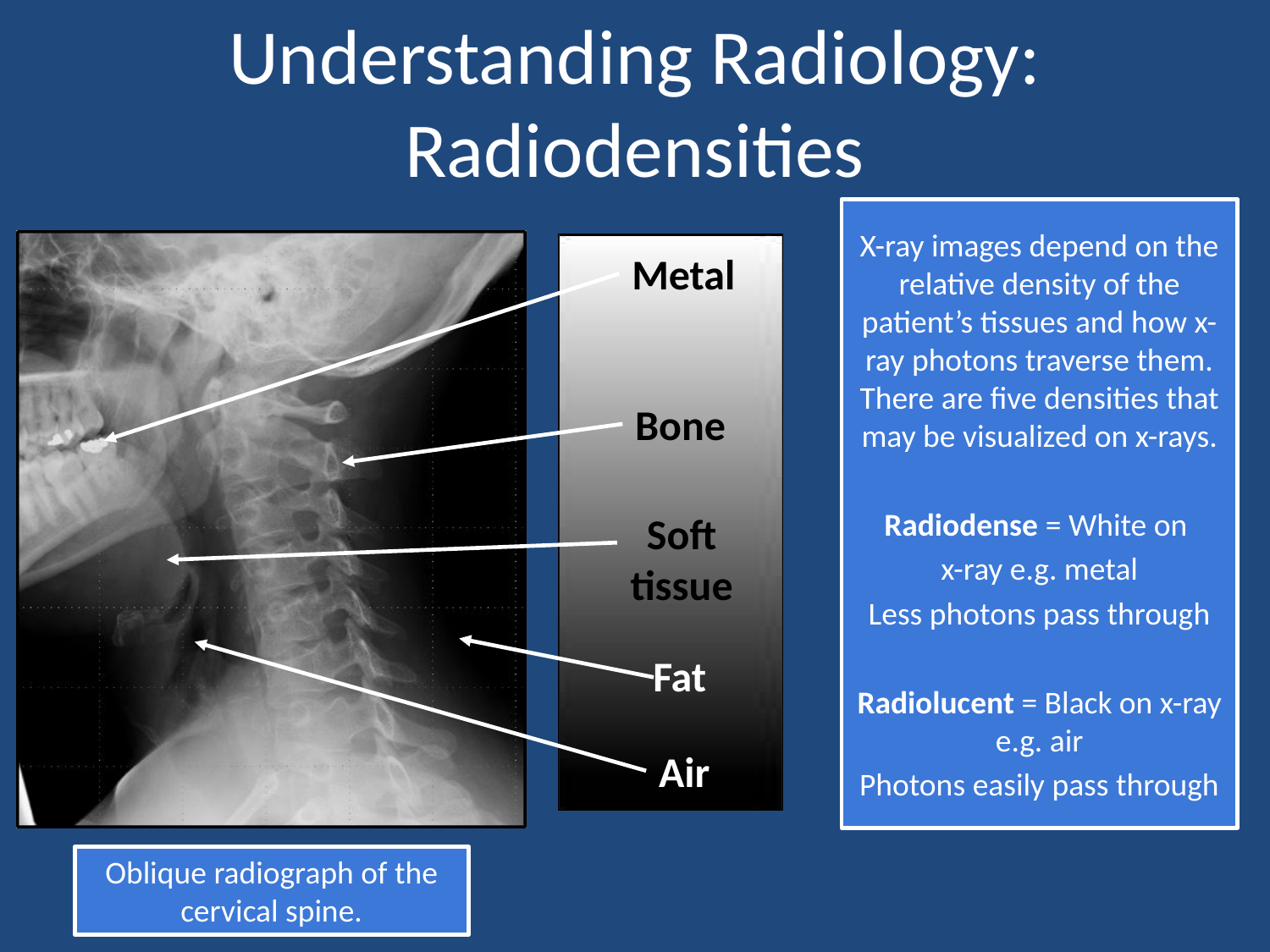

# Understanding Radiology: Radiodensities
X-ray images depend on the relative density of the patient’s tissues and how x-ray photons traverse them. There are five densities that may be visualized on x-rays.
Radiodense = White on
x-ray e.g. metal
Less photons pass through
Radiolucent = Black on x-ray e.g. air
Photons easily pass through
Metal
Bone
Soft tissue
Fat
Air
Oblique radiograph of the cervical spine.

## Slide 7
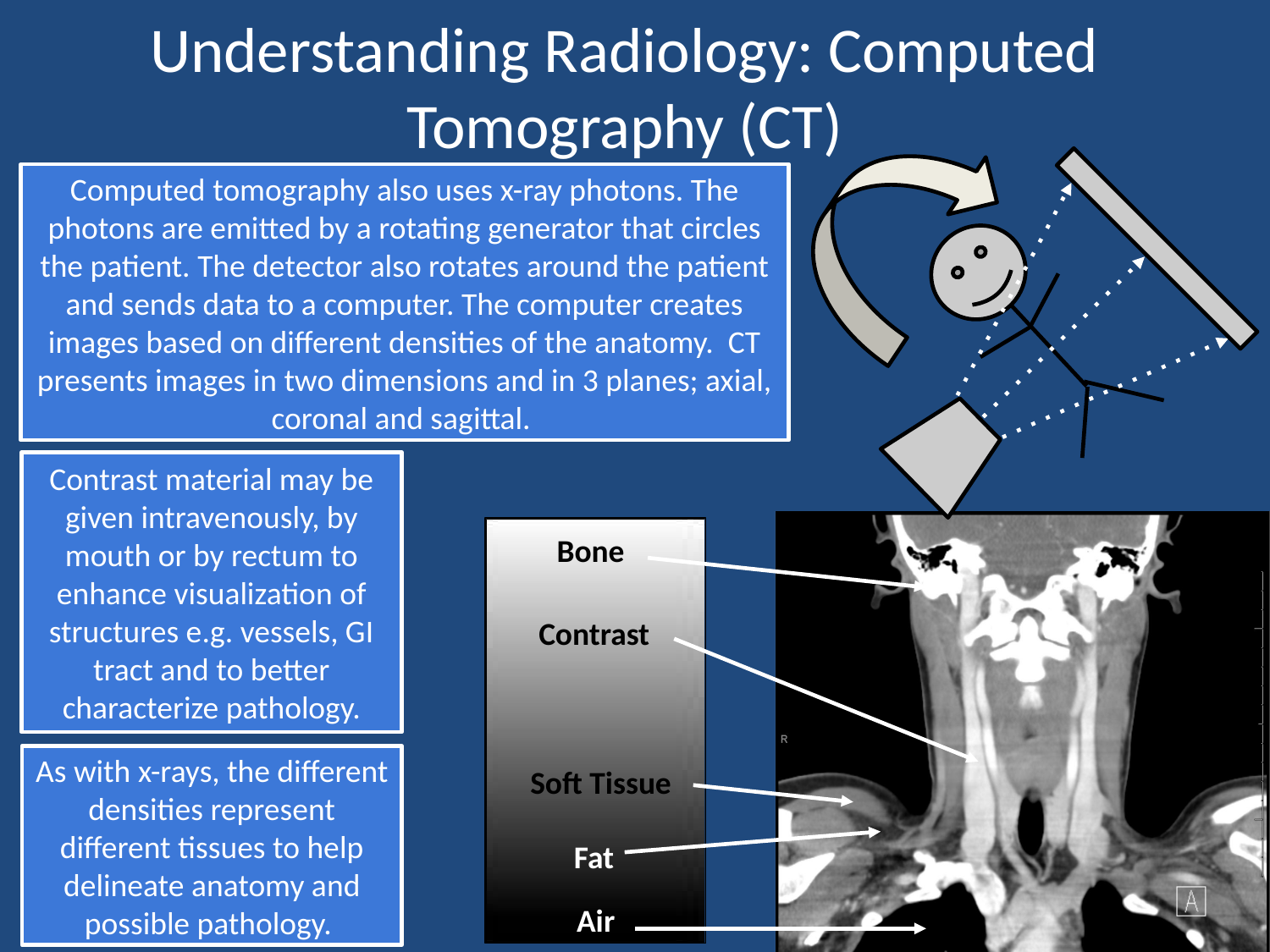

# Understanding Radiology: Computed Tomography (CT)
Computed tomography also uses x-ray photons. The photons are emitted by a rotating generator that circles the patient. The detector also rotates around the patient and sends data to a computer. The computer creates images based on different densities of the anatomy. CT presents images in two dimensions and in 3 planes; axial, coronal and sagittal.
Contrast material may be given intravenously, by mouth or by rectum to enhance visualization of structures e.g. vessels, GI tract and to better characterize pathology.
Bone
Contrast
As with x-rays, the different densities represent different tissues to help delineate anatomy and possible pathology.
Soft Tissue
Fat
Air

## Slide 8
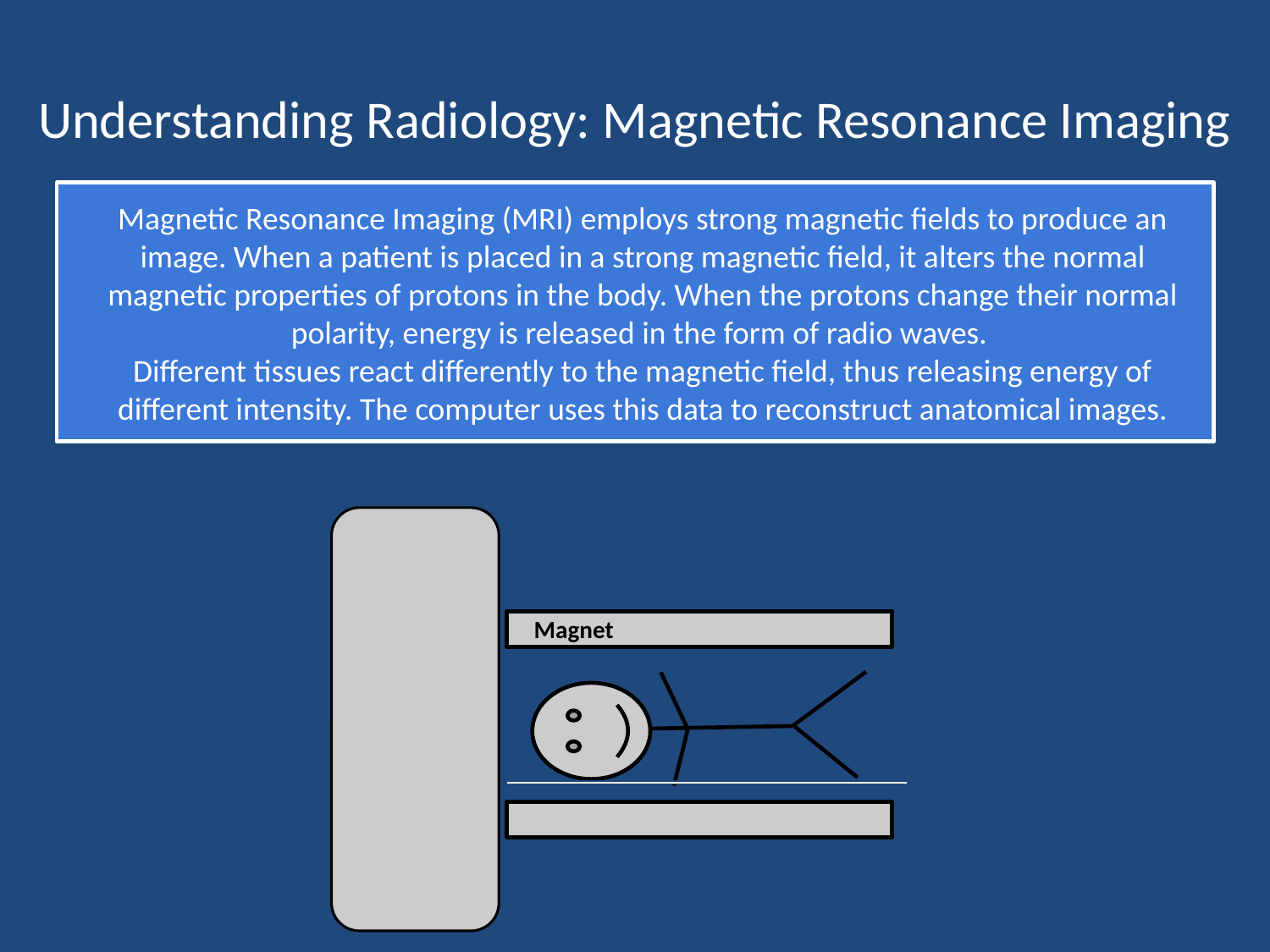

# Understanding Radiology: Magnetic Resonance Imaging
Magnetic Resonance Imaging (MRI) employs strong magnetic fields to produce an image. When a patient is placed in a strong magnetic field, it alters the normal magnetic properties of protons in the body. When the protons change their normal polarity, energy is released in the form of radio waves.
Different tissues react differently to the magnetic field, thus releasing energy of different intensity. The computer uses this data to reconstruct anatomical images.
Magnet

## Slide 9
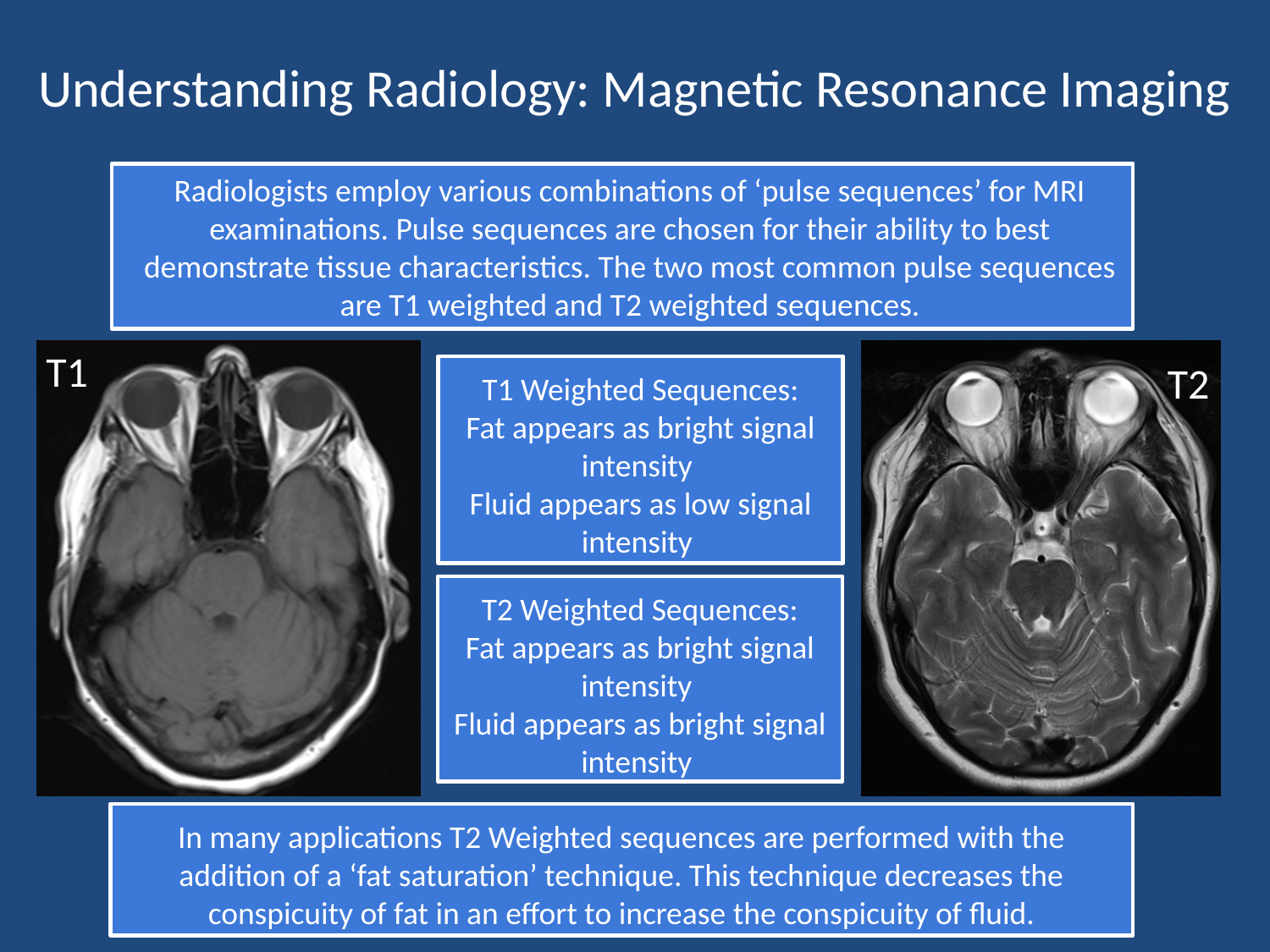

# Understanding Radiology: Magnetic Resonance Imaging
Radiologists employ various combinations of ‘pulse sequences’ for MRI examinations. Pulse sequences are chosen for their ability to best demonstrate tissue characteristics. The two most common pulse sequences are T1 weighted and T2 weighted sequences.
T1
T2
T1 Weighted Sequences:
Fat appears as bright signal intensity
Fluid appears as low signal intensity
T2 Weighted Sequences:
Fat appears as bright signal intensity
Fluid appears as bright signal intensity
In many applications T2 Weighted sequences are performed with the addition of a ‘fat saturation’ technique. This technique decreases the conspicuity of fat in an effort to increase the conspicuity of fluid.

## Slide 10
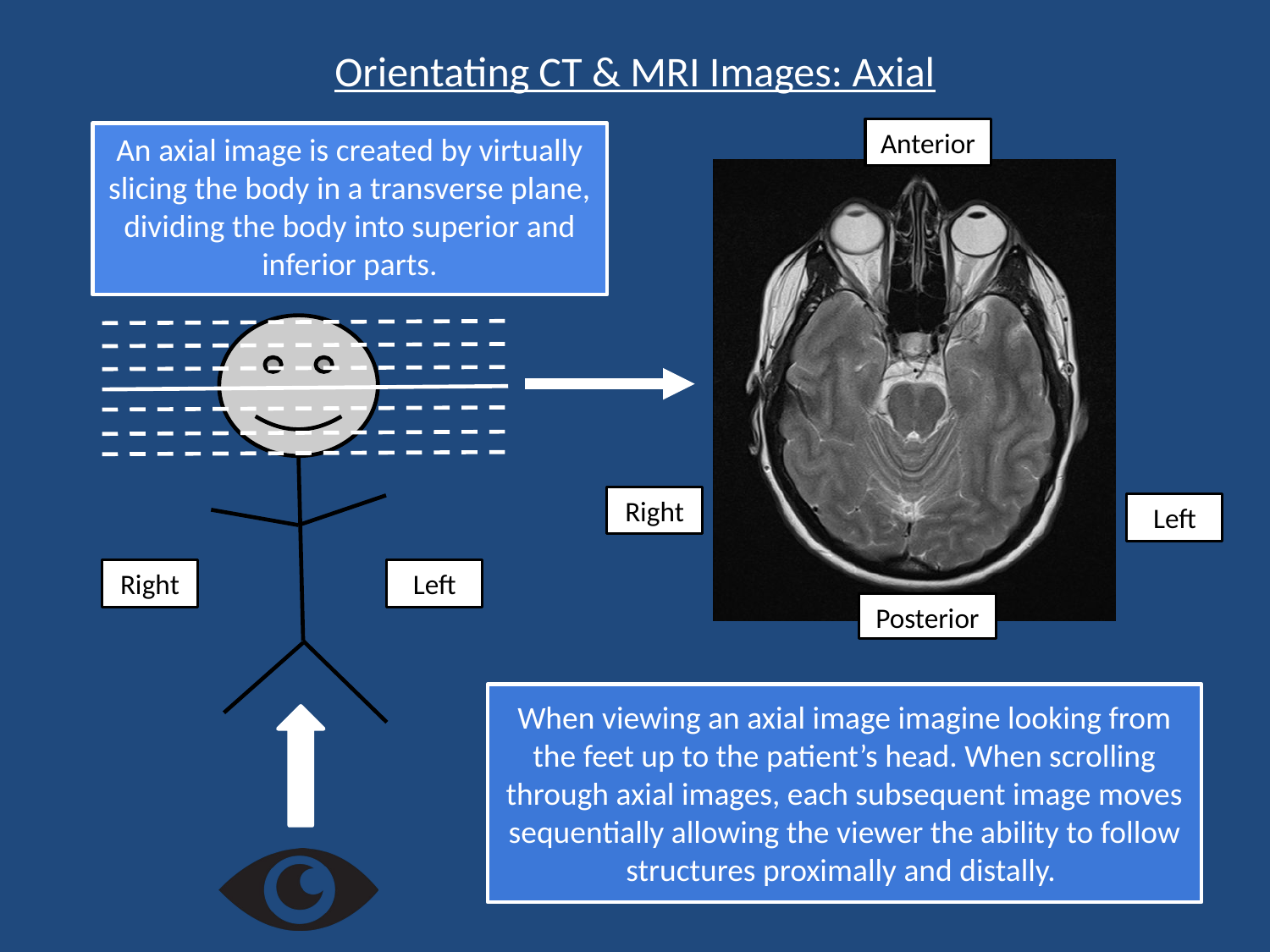

# Orientating CT & MRI Images: Axial
Anterior
Right
Left
Posterior
An axial image is created by virtually slicing the body in a transverse plane, dividing the body into superior and inferior parts.
Right
Left
When viewing an axial image imagine looking from the feet up to the patient’s head. When scrolling through axial images, each subsequent image moves sequentially allowing the viewer the ability to follow structures proximally and distally.

## Slide 11
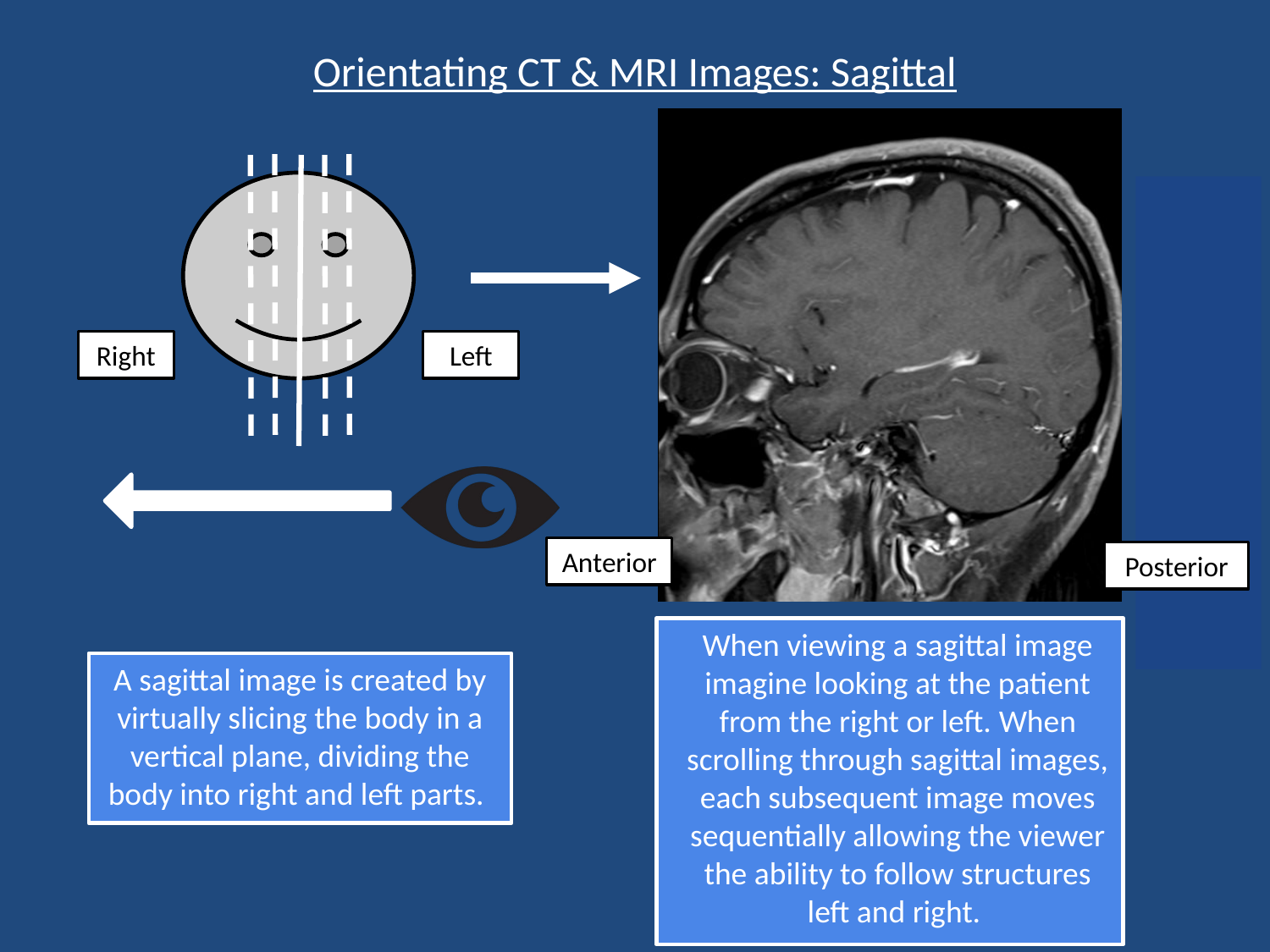

# Orientating CT & MRI Images: Sagittal
Anterior
Posterior
Right
Left
When viewing a sagittal image imagine looking at the patient from the right or left. When scrolling through sagittal images, each subsequent image moves sequentially allowing the viewer the ability to follow structures left and right.
A sagittal image is created by virtually slicing the body in a vertical plane, dividing the body into right and left parts.

## Slide 12
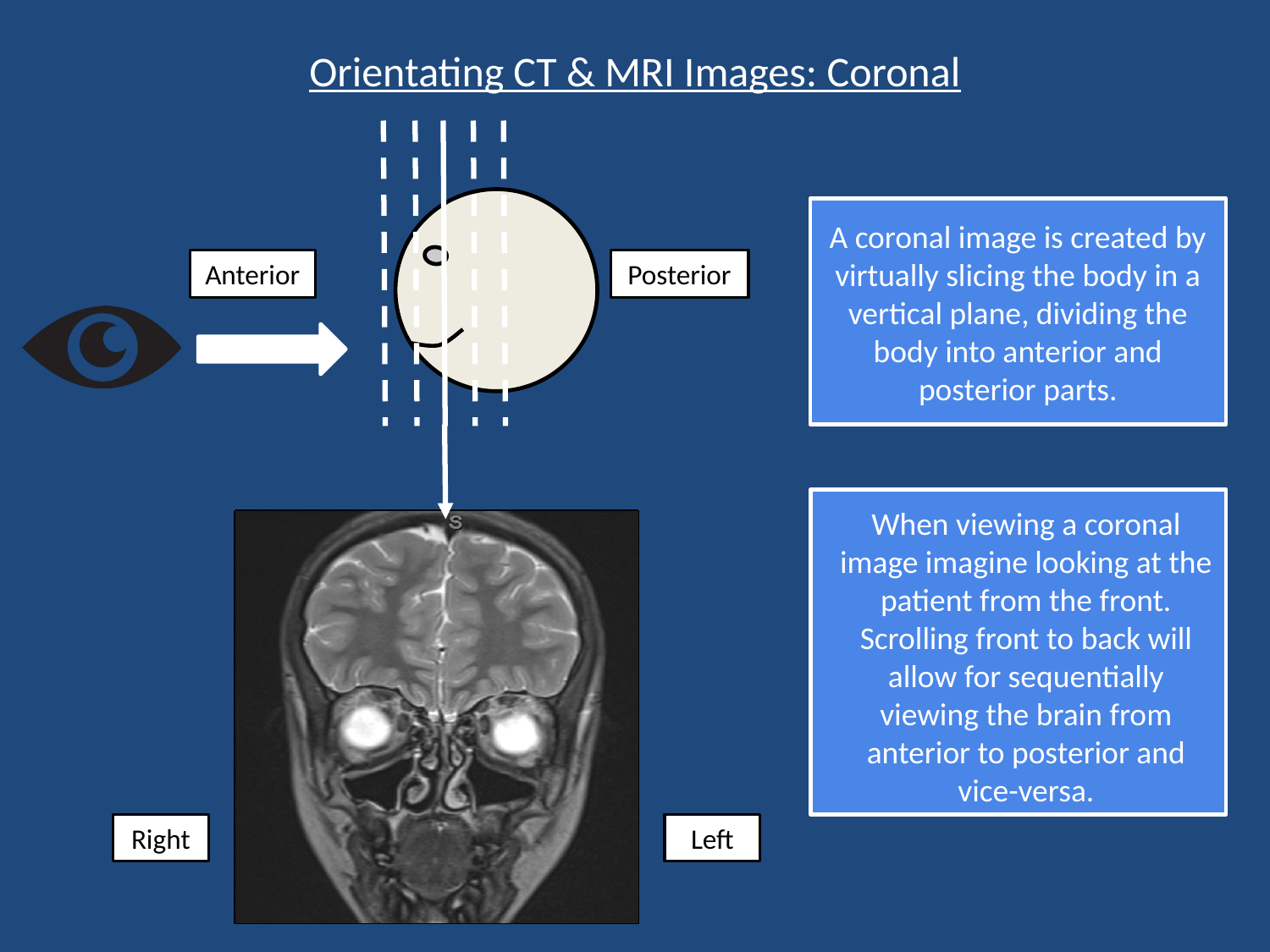

# Orientating CT & MRI Images: Coronal
A coronal image is created by virtually slicing the body in a vertical plane, dividing the body into anterior and posterior parts.
Anterior
Posterior
When viewing a coronal image imagine looking at the patient from the front. Scrolling front to back will allow for sequentially viewing the brain from anterior to posterior and vice-versa.
Right
Left

## Slide 13
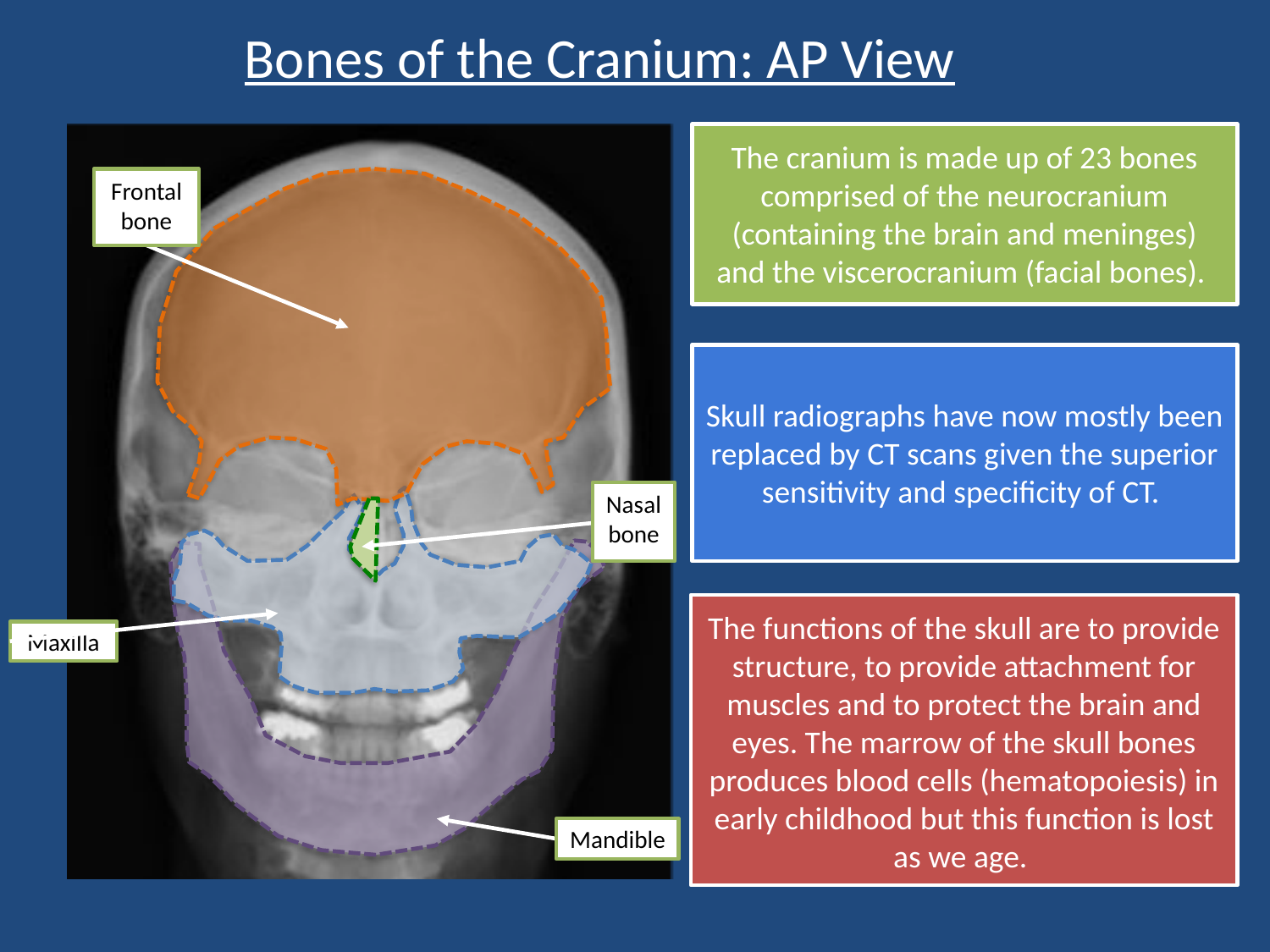

Bones of the Cranium: AP View
The cranium is made up of 23 bones comprised of the neurocranium (containing the brain and meninges) and the viscerocranium (facial bones).
Frontal bone
Skull radiographs have now mostly been replaced by CT scans given the superior sensitivity and specificity of CT.
Nasal bone
The functions of the skull are to provide structure, to provide attachment for muscles and to protect the brain and eyes. The marrow of the skull bones produces blood cells (hematopoiesis) in early childhood but this function is lost as we age.
Maxilla
Mandible

## Slide 14
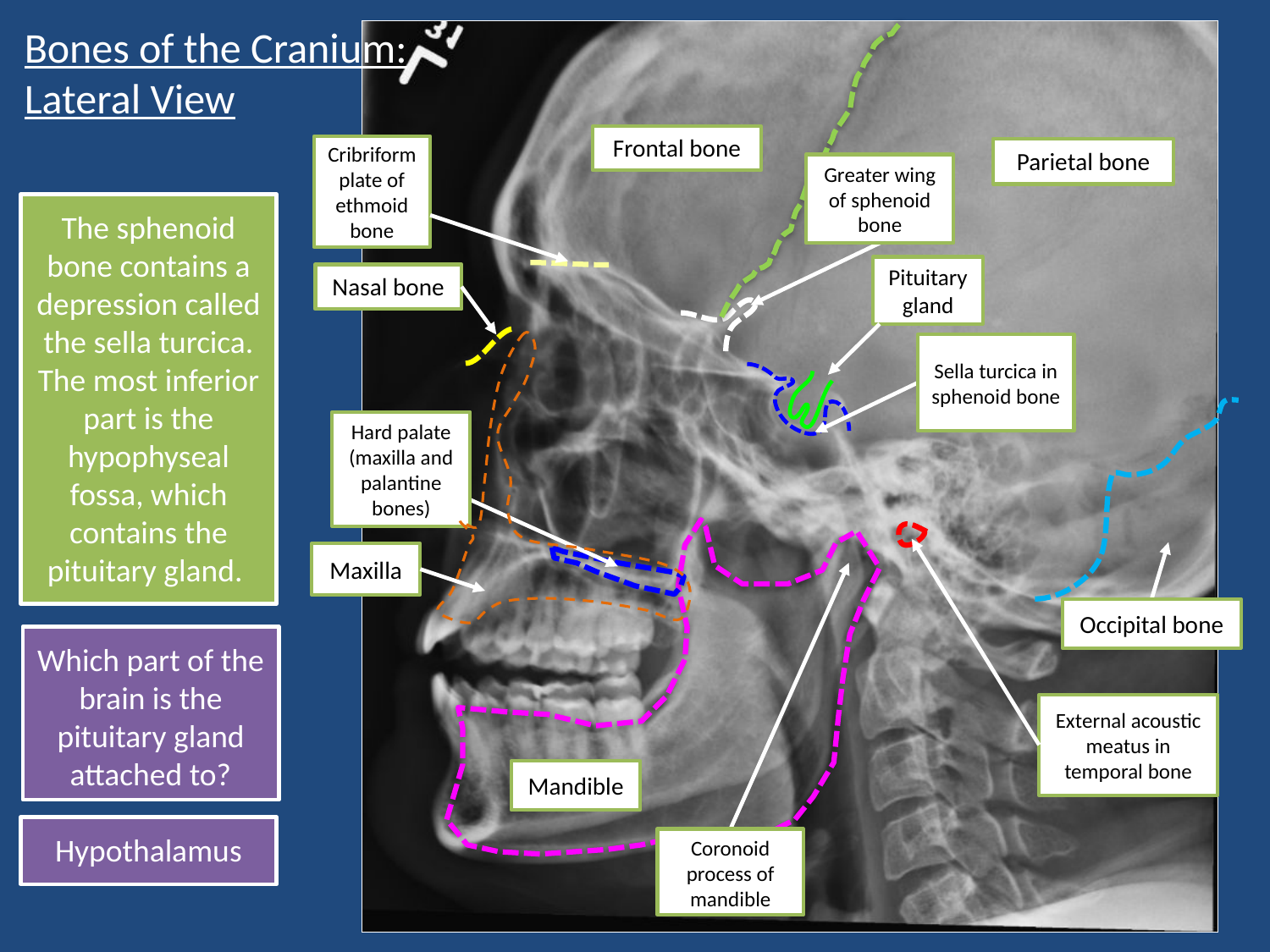

Bones of the Cranium: Lateral View
Frontal bone
Cribriform plate of ethmoid bone
Parietal bone
Greater wing of sphenoid bone
The sphenoid bone contains a depression called the sella turcica. The most inferior part is the hypophyseal fossa, which contains the pituitary gland.
Pituitary gland
Nasal bone
Sella turcica in sphenoid bone
Hard palate (maxilla and palantine bones)
Occipital bone
Maxilla
Which part of the brain is the pituitary gland attached to?
External acoustic meatus in temporal bone
Mandible
Hypothalamus
Coronoid process of mandible

## Slide 15
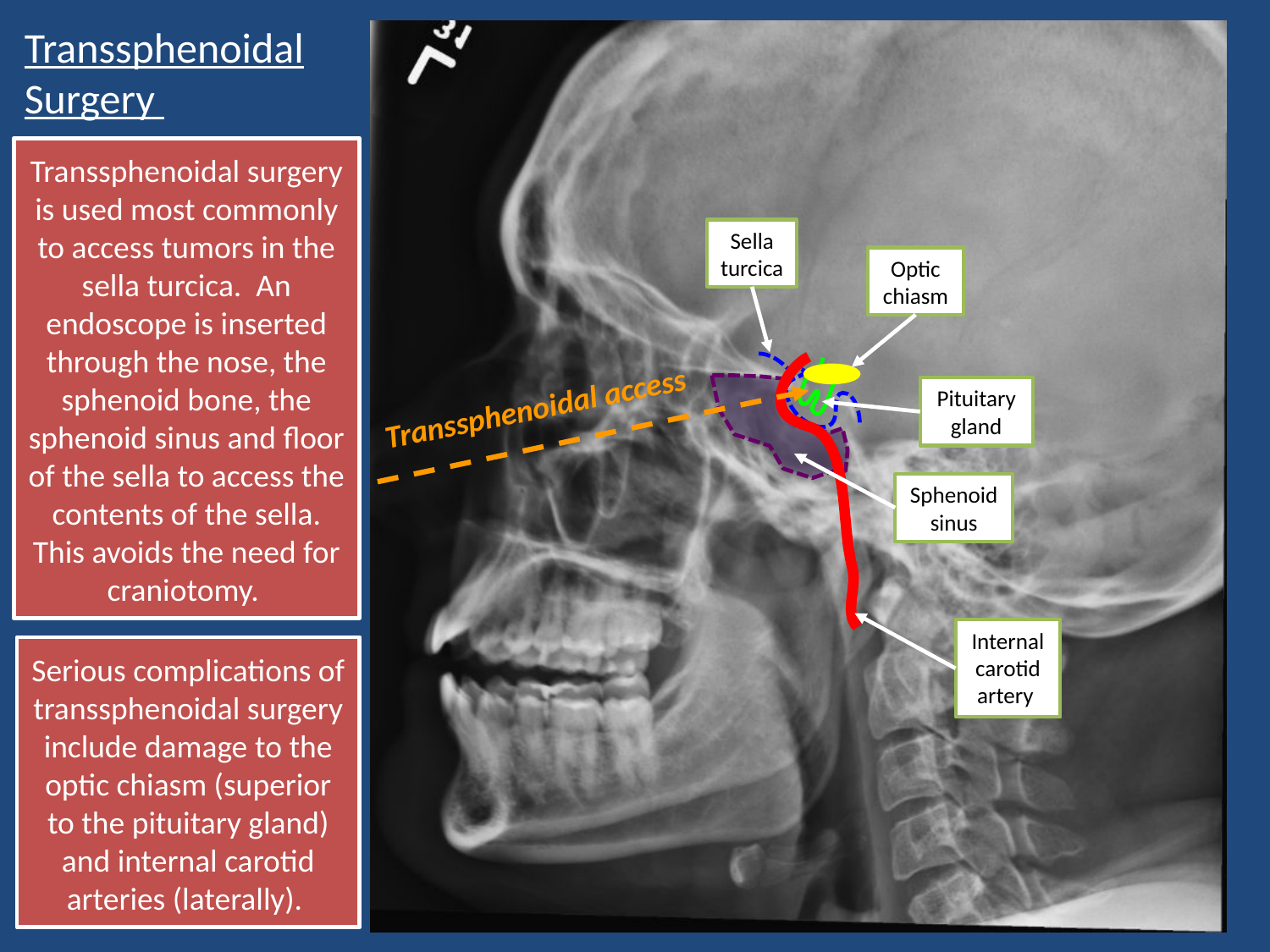

Transsphenoidal Surgery
Transsphenoidal surgery is used most commonly to access tumors in the sella turcica. An endoscope is inserted through the nose, the sphenoid bone, the sphenoid sinus and floor of the sella to access the contents of the sella. This avoids the need for craniotomy.
Sella turcica
Optic chiasm
Transsphenoidal access
Pituitary gland
Sphenoid sinus
Internal carotid artery
Serious complications of transsphenoidal surgery include damage to the optic chiasm (superior to the pituitary gland) and internal carotid arteries (laterally).

## Slide 16
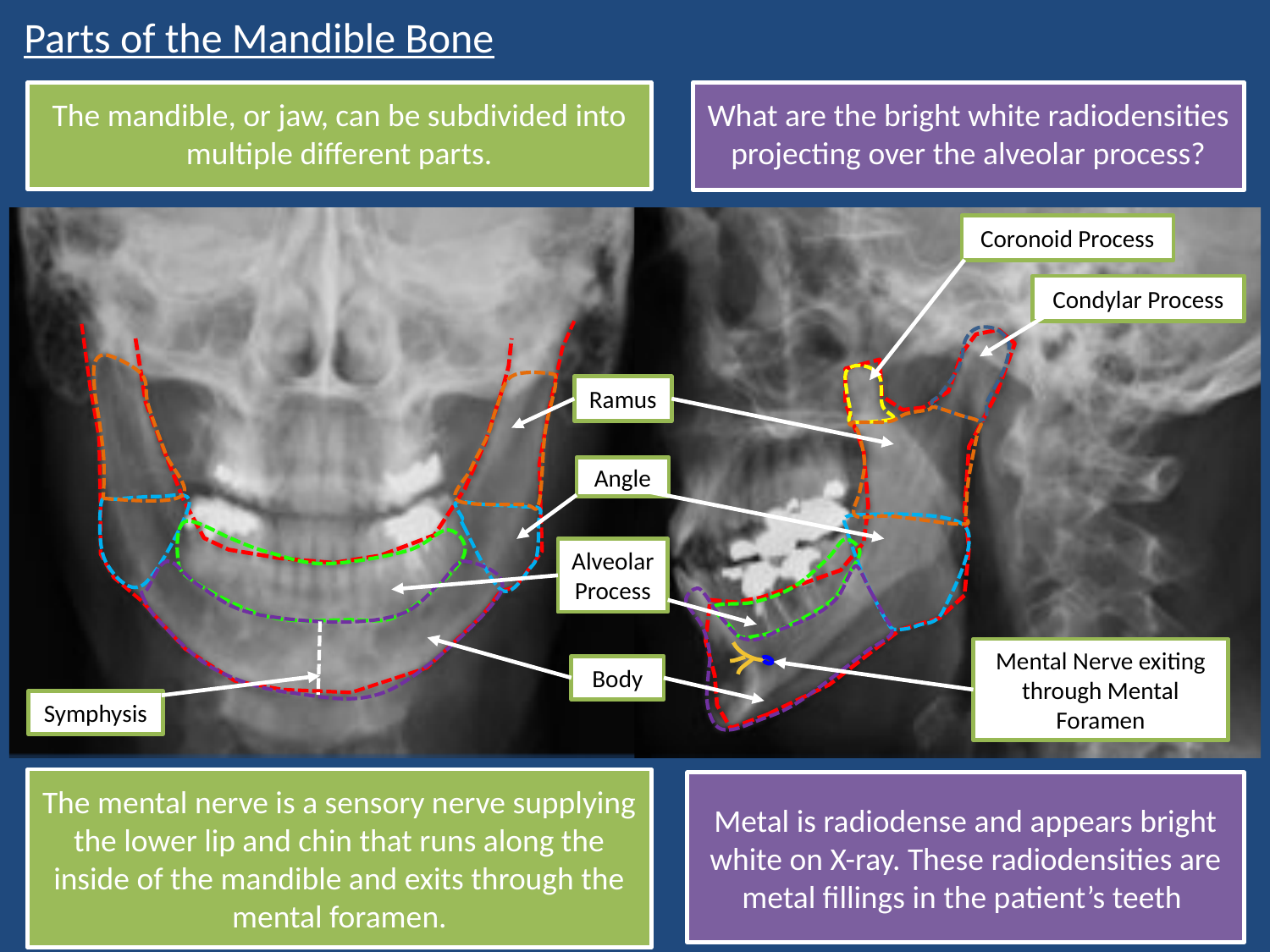

Parts of the Mandible Bone
The mandible, or jaw, can be subdivided into multiple different parts.
What are the bright white radiodensities projecting over the alveolar process?
Coronoid Process
Condylar Process
Ramus
Angle
Alveolar Process
Mental Nerve exiting through Mental Foramen
Body
Symphysis
The mental nerve is a sensory nerve supplying the lower lip and chin that runs along the inside of the mandible and exits through the mental foramen.
Metal is radiodense and appears bright white on X-ray. These radiodensities are metal fillings in the patient’s teeth

## Slide 17
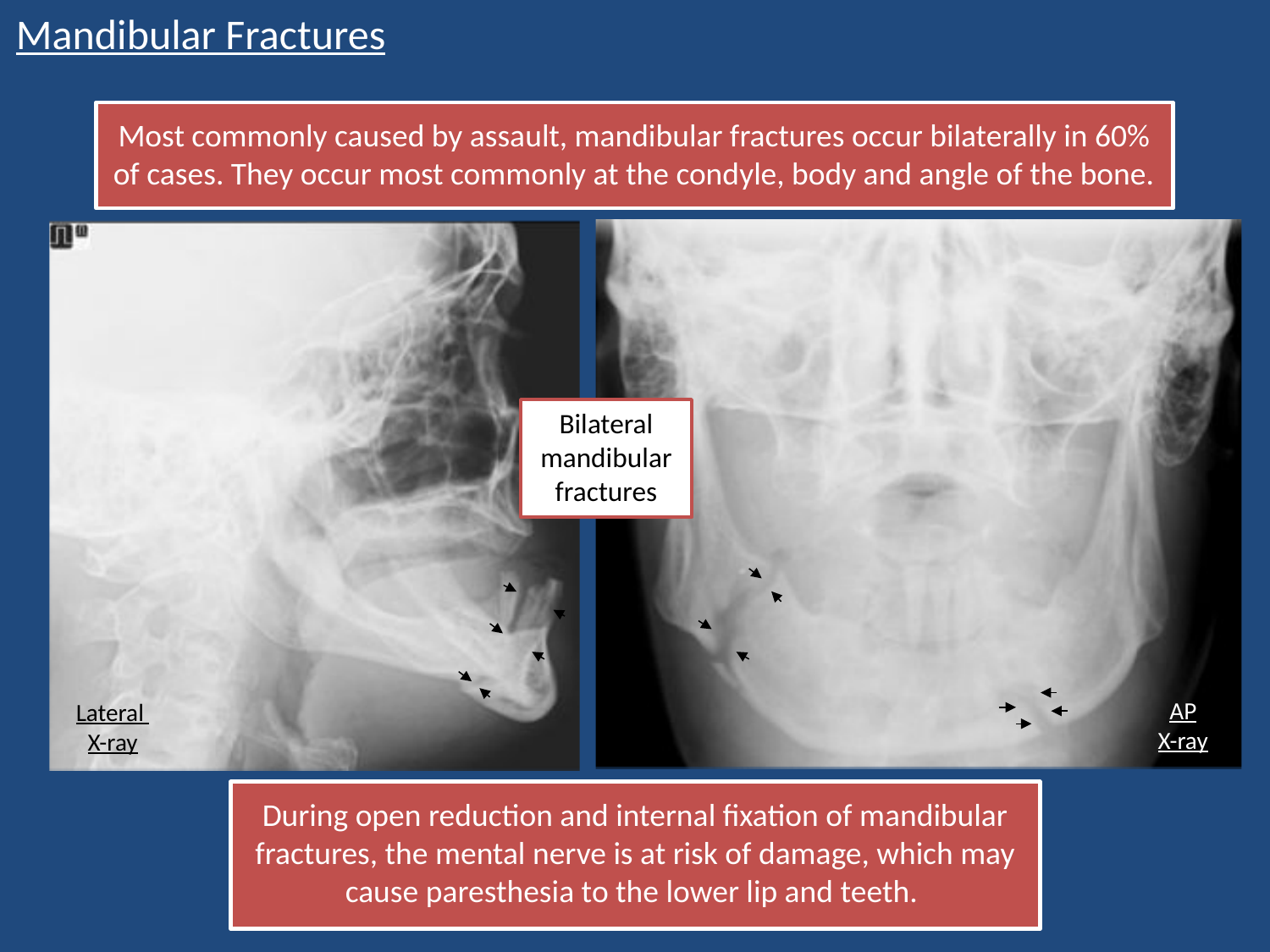

Mandibular Fractures
Most commonly caused by assault, mandibular fractures occur bilaterally in 60% of cases. They occur most commonly at the condyle, body and angle of the bone.
Bilateral mandibular fractures
AP
X-ray
Lateral
X-ray
During open reduction and internal fixation of mandibular fractures, the mental nerve is at risk of damage, which may cause paresthesia to the lower lip and teeth.

## Slide 18
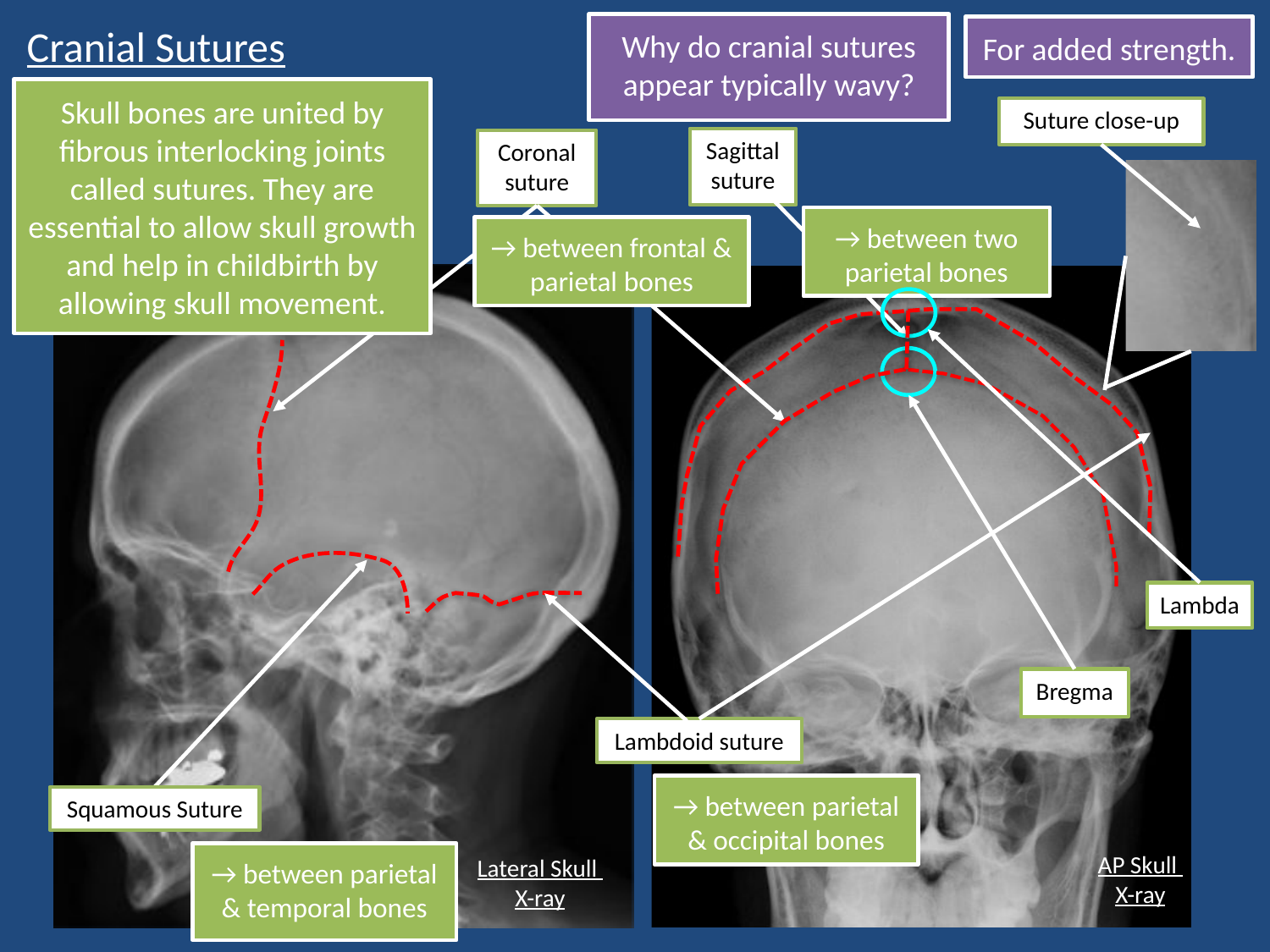

Cranial Sutures
Why do cranial sutures appear typically wavy?
For added strength.
Skull bones are united by fibrous interlocking joints called sutures. They are essential to allow skull growth and help in childbirth by allowing skull movement.
Suture close-up
Sagittal suture
Coronal suture
→ between two parietal bones
→ between frontal & parietal bones
Lambda
Bregma
Lambdoid suture
Squamous Suture
→ between parietal & occipital bones
AP Skull
X-ray
Lateral Skull
X-ray
→ between parietal & temporal bones

## Slide 19
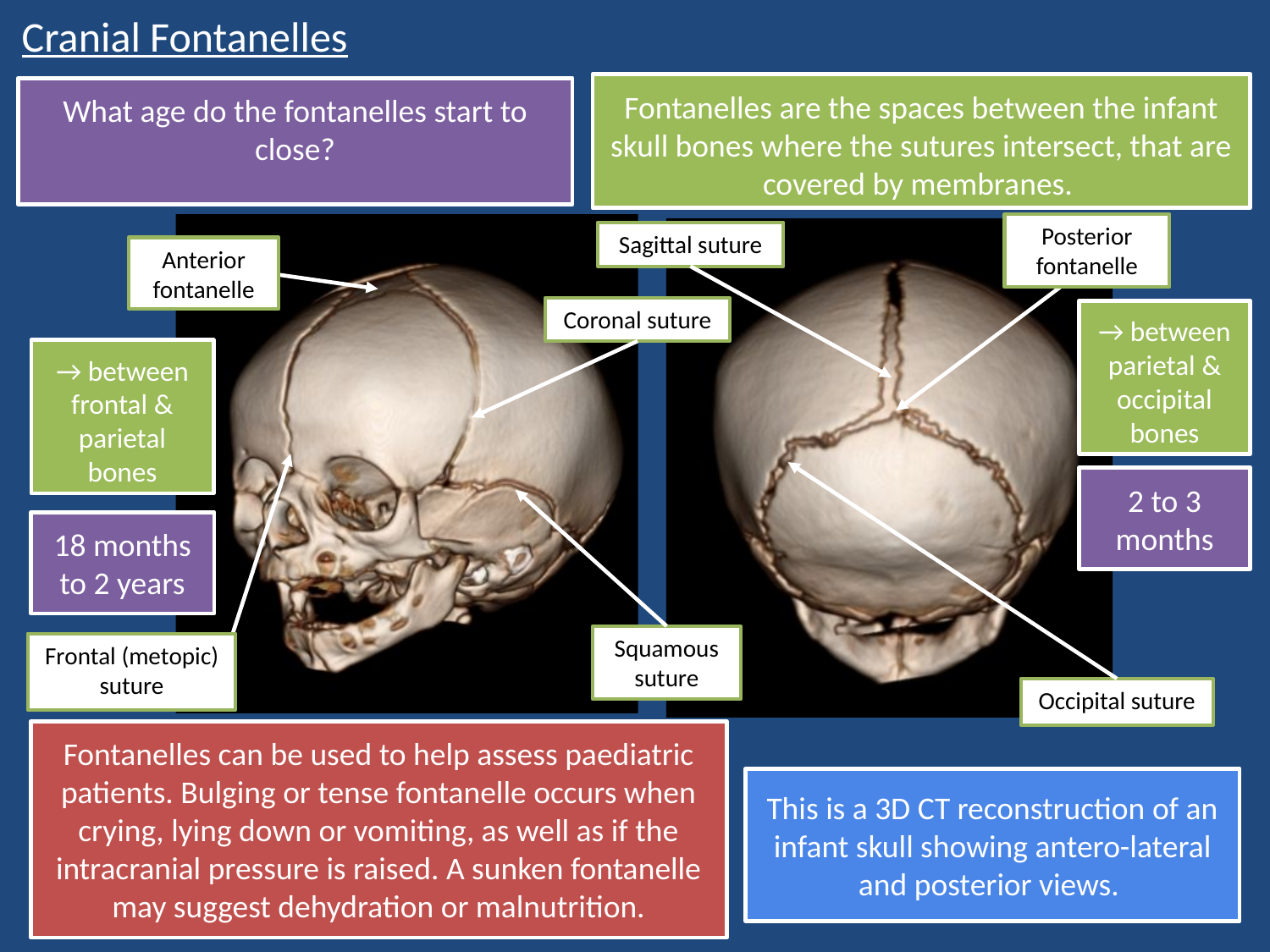

Cranial Fontanelles
Fontanelles are the spaces between the infant skull bones where the sutures intersect, that are covered by membranes.
What age do the fontanelles start to close?
Posterior fontanelle
Sagittal suture
Anterior fontanelle
Coronal suture
→ between parietal & occipital bones
→ between frontal & parietal bones
Frontal (metopic) suture
Occipital suture
2 to 3 months
Squamous suture
18 months to 2 years
Fontanelles can be used to help assess paediatric patients. Bulging or tense fontanelle occurs when crying, lying down or vomiting, as well as if the intracranial pressure is raised. A sunken fontanelle may suggest dehydration or malnutrition.
This is a 3D CT reconstruction of an infant skull showing antero-lateral and posterior views.

## Slide 20
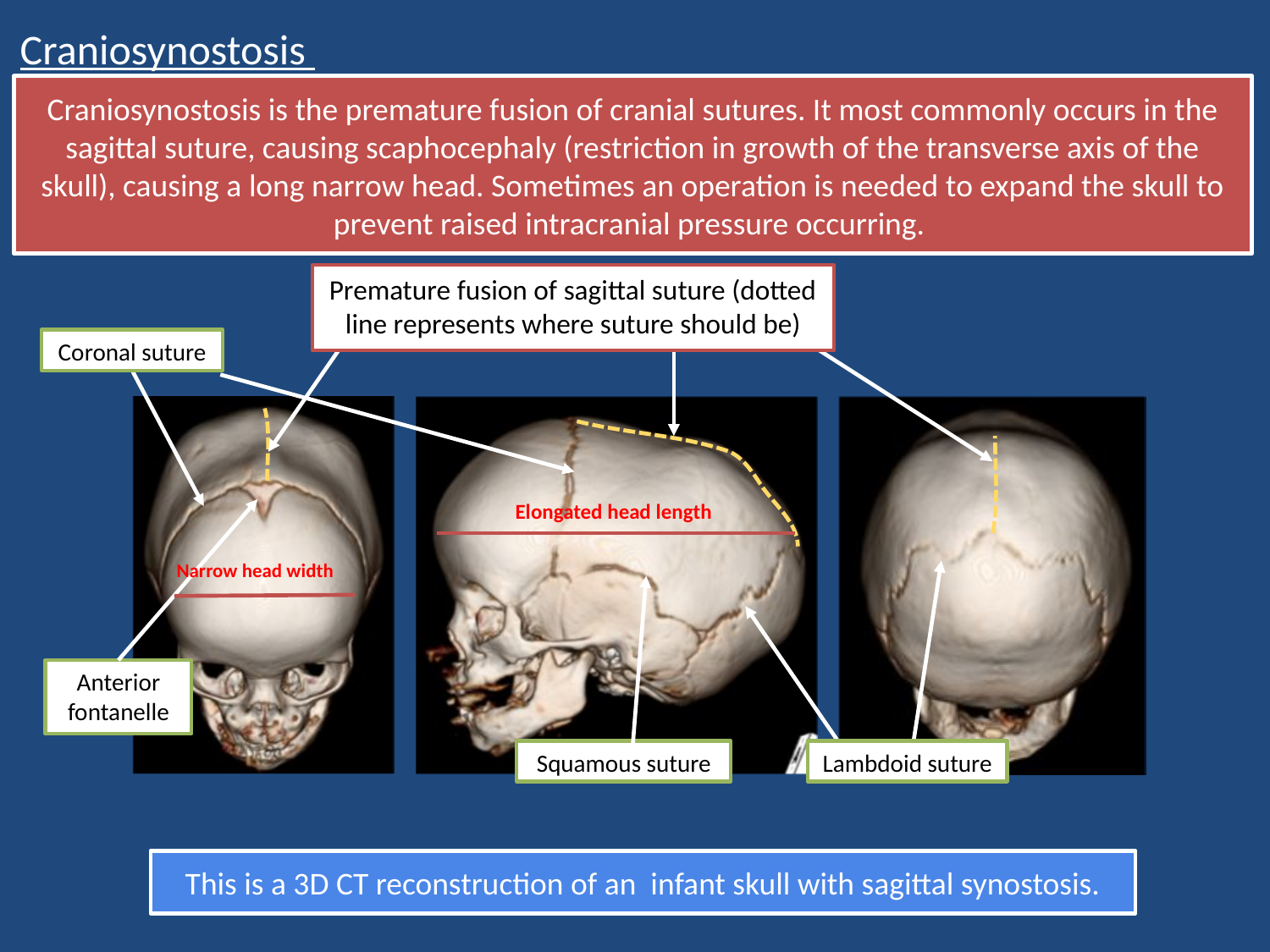

Craniosynostosis
Craniosynostosis is the premature fusion of cranial sutures. It most commonly occurs in the sagittal suture, causing scaphocephaly (restriction in growth of the transverse axis of the skull), causing a long narrow head. Sometimes an operation is needed to expand the skull to prevent raised intracranial pressure occurring.
Premature fusion of sagittal suture (dotted line represents where suture should be)
Coronal suture
Elongated head length
Anterior fontanelle
Narrow head width
Lambdoid suture
Squamous suture
This is a 3D CT reconstruction of an infant skull with sagittal synostosis.

## Slide 21
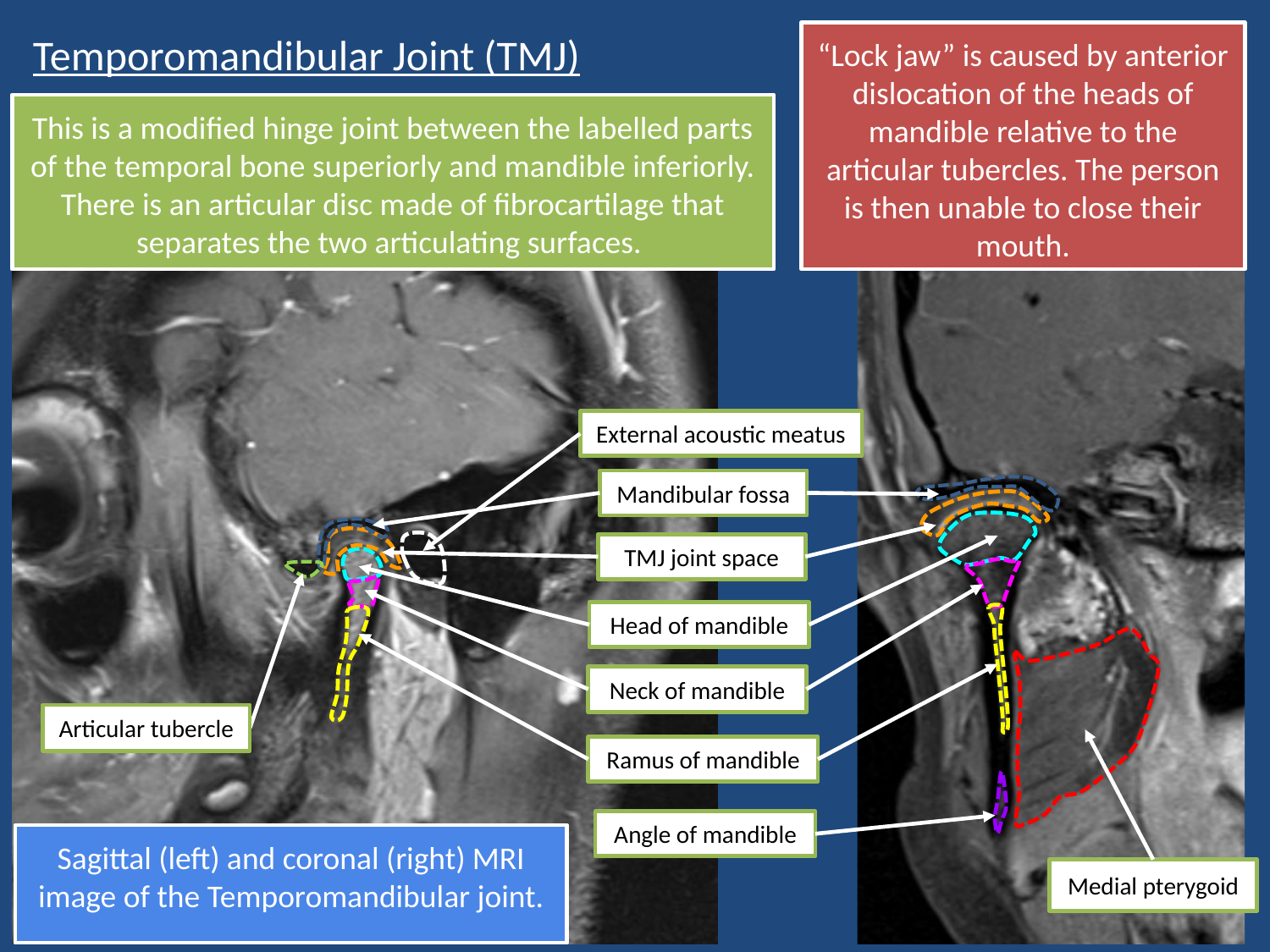

Temporomandibular Joint (TMJ)
“Lock jaw” is caused by anterior dislocation of the heads of mandible relative to the articular tubercles. The person is then unable to close their mouth.
This is a modified hinge joint between the labelled parts of the temporal bone superiorly and mandible inferiorly. There is an articular disc made of fibrocartilage that separates the two articulating surfaces.
External acoustic meatus
Mandibular fossa
TMJ joint space
Head of mandible
Articular tubercle
Neck of mandible
Ramus of mandible
Medial pterygoid
Angle of mandible
Sagittal (left) and coronal (right) MRI image of the Temporomandibular joint.

## Slide 22
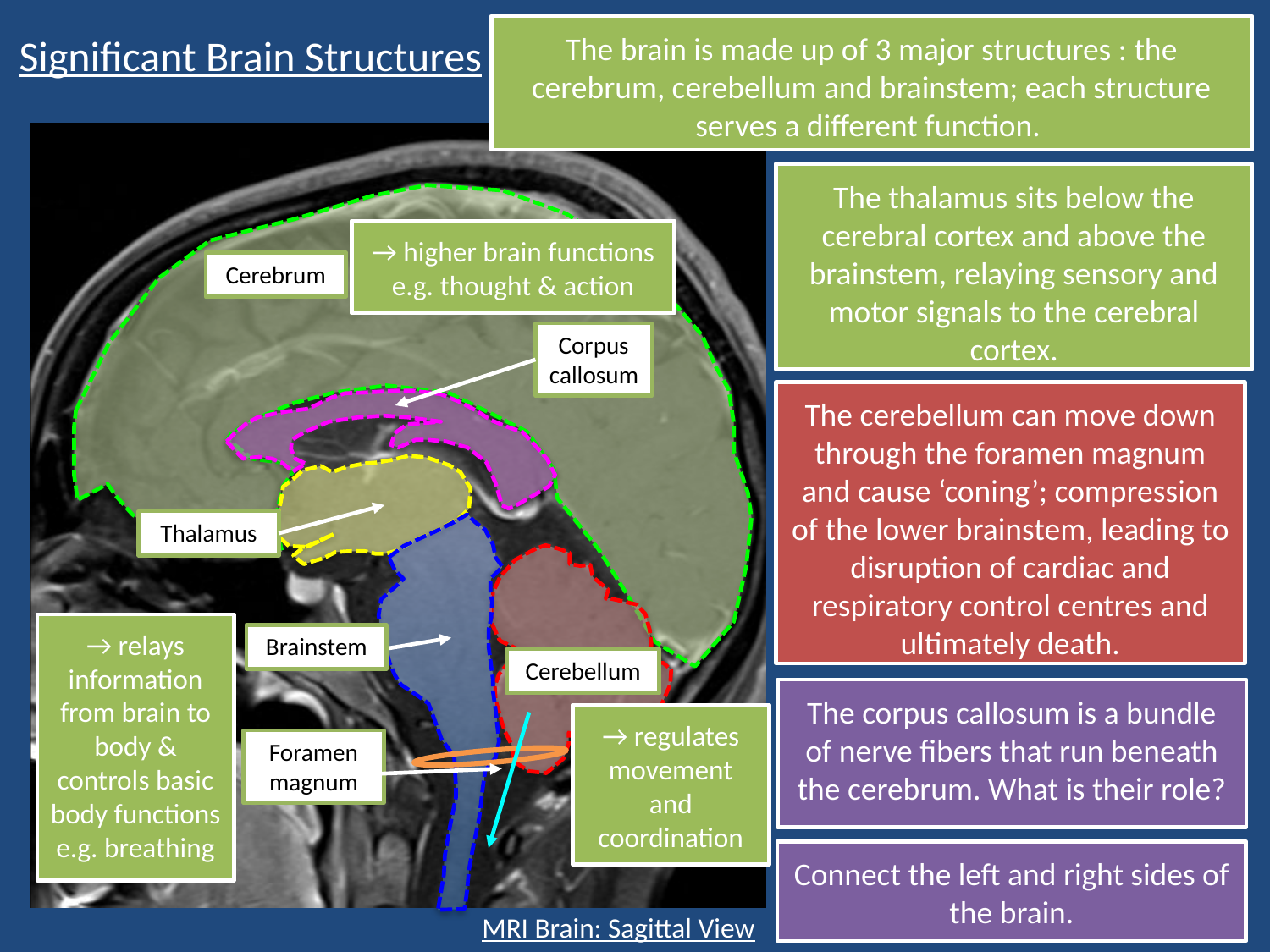

The brain is made up of 3 major structures : the cerebrum, cerebellum and brainstem; each structure serves a different function.
Significant Brain Structures
The thalamus sits below the cerebral cortex and above the brainstem, relaying sensory and motor signals to the cerebral cortex.
→ higher brain functions e.g. thought & action
Cerebrum
Corpus callosum
The cerebellum can move down through the foramen magnum and cause ‘coning’; compression of the lower brainstem, leading to disruption of cardiac and respiratory control centres and ultimately death.
Thalamus
→ relays information from brain to body & controls basic body functions e.g. breathing
Brainstem
Cerebellum
The corpus callosum is a bundle of nerve fibers that run beneath the cerebrum. What is their role?
→ regulates movement and coordination
Foramen magnum
Connect the left and right sides of the brain.
MRI Brain: Sagittal View

## Slide 23
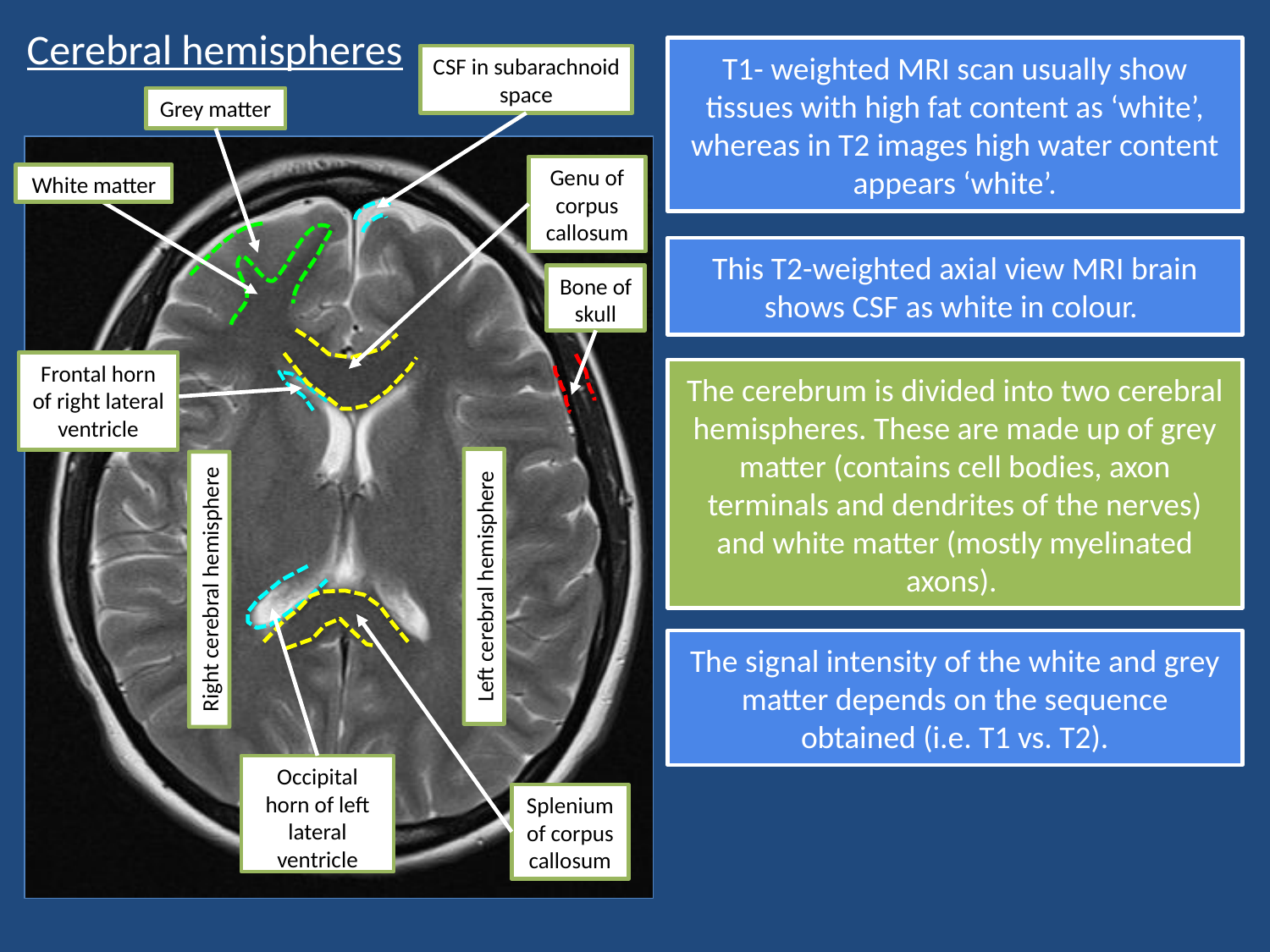

Cerebral hemispheres
T1- weighted MRI scan usually show tissues with high fat content as ‘white’, whereas in T2 images high water content appears ‘white’.
CSF in subarachnoid space
Grey matter
Genu of corpus callosum
White matter
This T2-weighted axial view MRI brain shows CSF as white in colour.
Bone of skull
Frontal horn of right lateral ventricle
The cerebrum is divided into two cerebral hemispheres. These are made up of grey matter (contains cell bodies, axon terminals and dendrites of the nerves) and white matter (mostly myelinated axons).
Left cerebral hemisphere
Right cerebral hemisphere
Occipital horn of left lateral ventricle
Splenium of corpus callosum
The signal intensity of the white and grey matter depends on the sequence obtained (i.e. T1 vs. T2).

## Slide 24
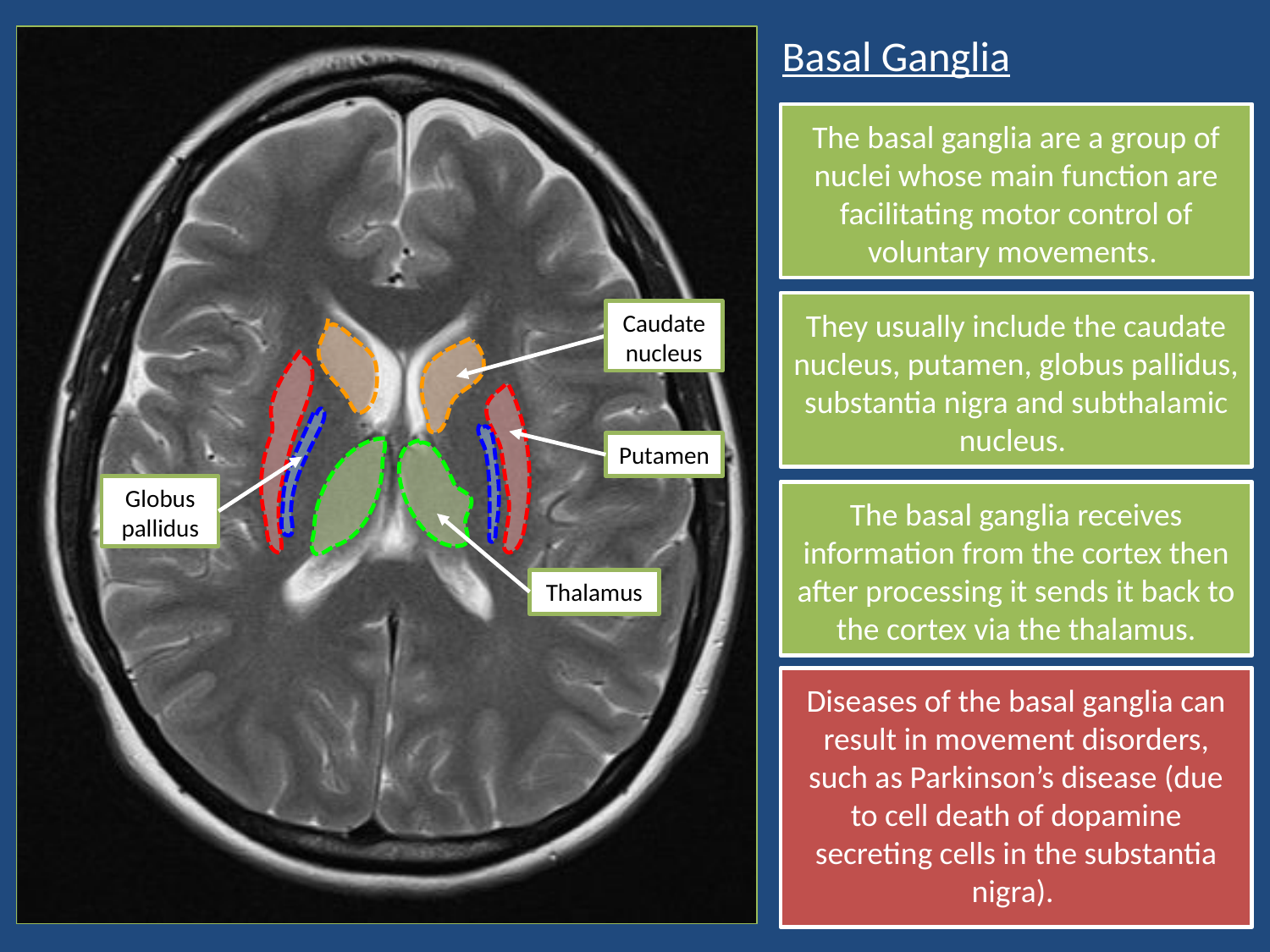

Basal Ganglia
The basal ganglia are a group of nuclei whose main function are facilitating motor control of voluntary movements.
They usually include the caudate nucleus, putamen, globus pallidus, substantia nigra and subthalamic nucleus.
Caudate nucleus
Putamen
Globus pallidus
The basal ganglia receives information from the cortex then after processing it sends it back to the cortex via the thalamus.
Thalamus
Diseases of the basal ganglia can result in movement disorders, such as Parkinson’s disease (due to cell death of dopamine secreting cells in the substantia nigra).

## Slide 25
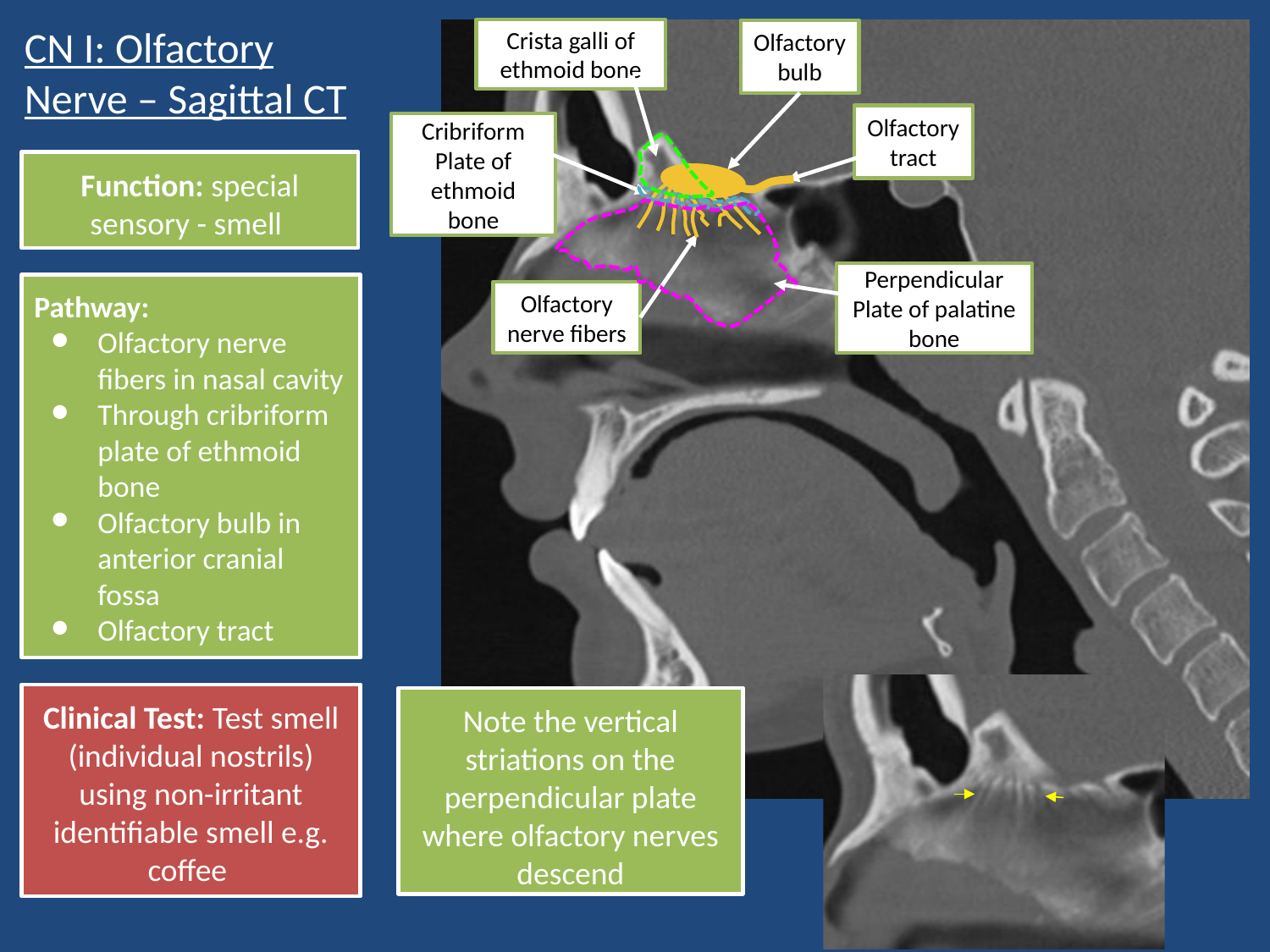

CN I: Olfactory Nerve – Sagittal CT
Crista galli of ethmoid bone
Olfactory bulb
Olfactory tract
Cribriform Plate of ethmoid bone
Function: special sensory - smell
Olfactory nerve fibers
Perpendicular Plate of palatine bone
Pathway:
Olfactory nerve fibers in nasal cavity
Through cribriform plate of ethmoid bone
Olfactory bulb in anterior cranial fossa
Olfactory tract
Clinical Test: Test smell (individual nostrils) using non-irritant identifiable smell e.g. coffee
Note the vertical striations on the perpendicular plate where olfactory nerves descend

## Slide 26
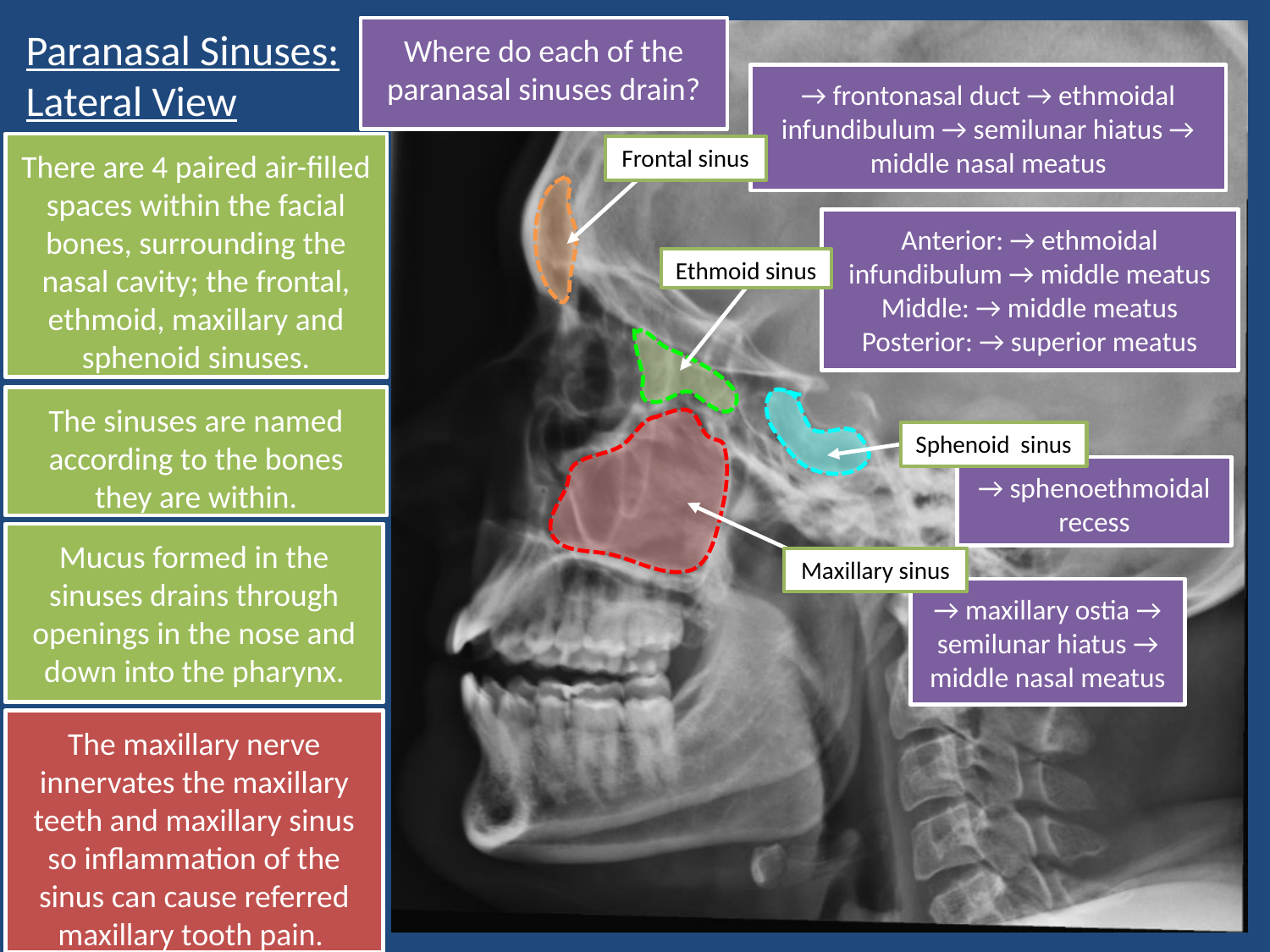

Paranasal Sinuses: Lateral View
Where do each of the paranasal sinuses drain?
→ frontonasal duct → ethmoidal infundibulum → semilunar hiatus → middle nasal meatus
There are 4 paired air-filled spaces within the facial bones, surrounding the nasal cavity; the frontal, ethmoid, maxillary and sphenoid sinuses.
Frontal sinus
Anterior: → ethmoidal infundibulum → middle meatus
Middle: → middle meatus
Posterior: → superior meatus
Ethmoid sinus
The sinuses are named according to the bones they are within.
Sphenoid sinus
→ sphenoethmoidal recess
Maxillary sinus
Mucus formed in the sinuses drains through openings in the nose and down into the pharynx.
→ maxillary ostia → semilunar hiatus → middle nasal meatus
The maxillary nerve innervates the maxillary teeth and maxillary sinus so inflammation of the sinus can cause referred maxillary tooth pain.

## Slide 27
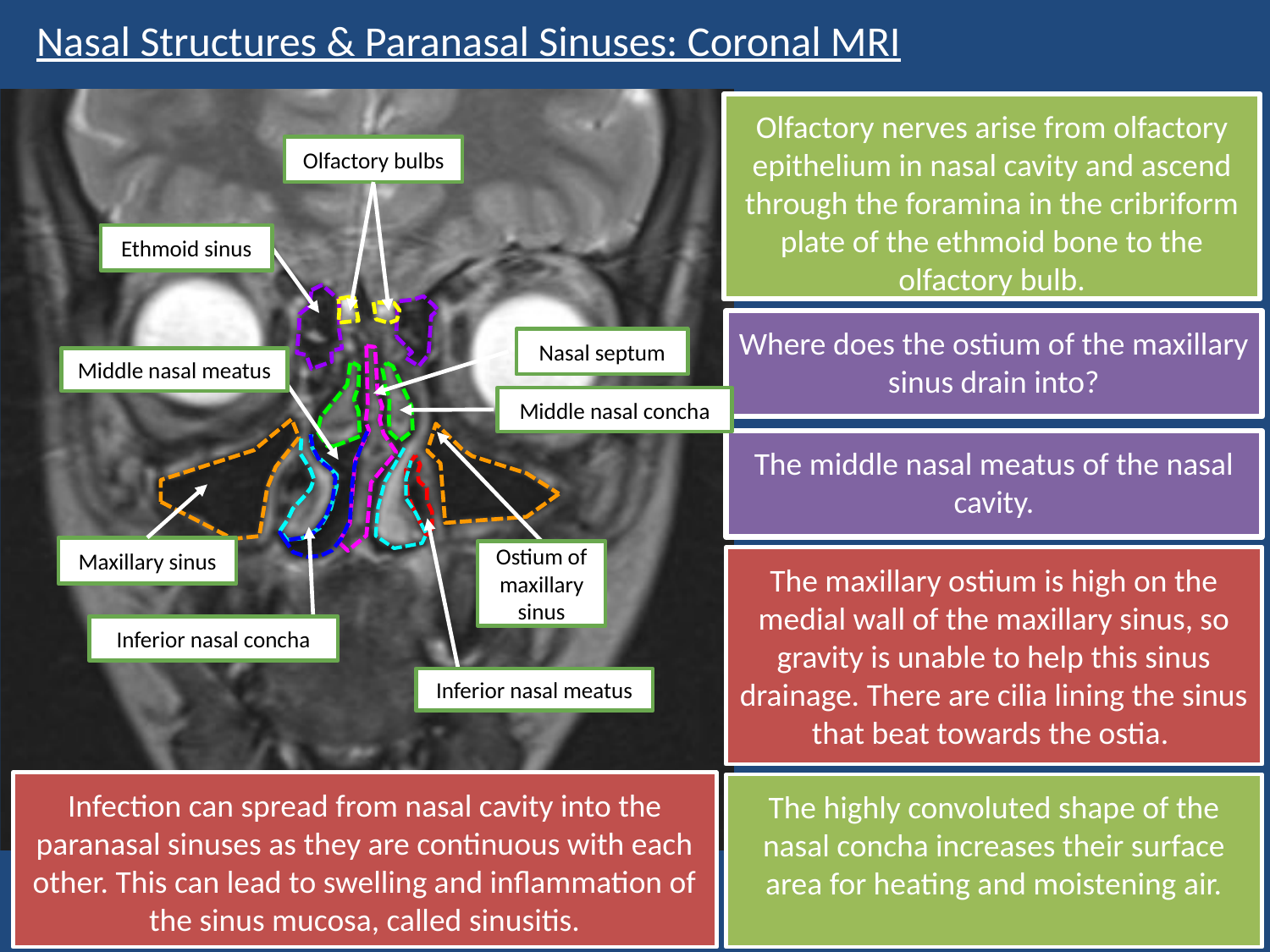

Nasal Structures & Paranasal Sinuses: Coronal MRI
Olfactory nerves arise from olfactory epithelium in nasal cavity and ascend through the foramina in the cribriform plate of the ethmoid bone to the olfactory bulb.
Olfactory bulbs
Ethmoid sinus
Where does the ostium of the maxillary sinus drain into?
Nasal septum
Middle nasal meatus
Middle nasal concha
The middle nasal meatus of the nasal cavity.
Ostium of maxillary sinus
Maxillary sinus
Inferior nasal meatus
Inferior nasal concha
The maxillary ostium is high on the medial wall of the maxillary sinus, so gravity is unable to help this sinus drainage. There are cilia lining the sinus that beat towards the ostia.
Infection can spread from nasal cavity into the paranasal sinuses as they are continuous with each other. This can lead to swelling and inflammation of the sinus mucosa, called sinusitis.
The highly convoluted shape of the nasal concha increases their surface area for heating and moistening air.

## Slide 28
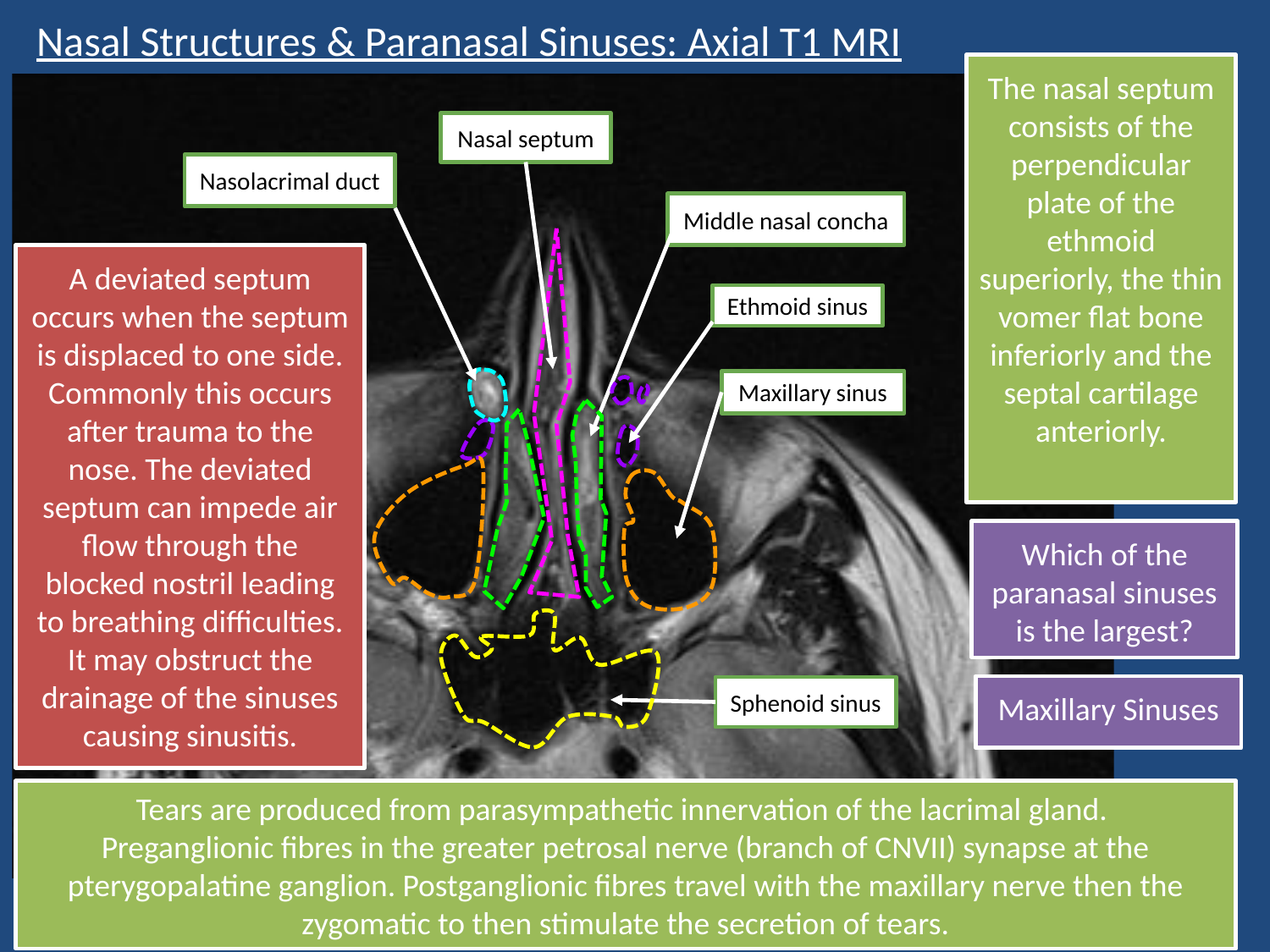

Nasal Structures & Paranasal Sinuses: Axial T1 MRI
The nasal septum consists of the perpendicular plate of the ethmoid superiorly, the thin vomer flat bone inferiorly and the septal cartilage anteriorly.
Nasal septum
Nasolacrimal duct
Middle nasal concha
A deviated septum occurs when the septum is displaced to one side. Commonly this occurs after trauma to the nose. The deviated septum can impede air flow through the blocked nostril leading to breathing difficulties. It may obstruct the drainage of the sinuses causing sinusitis.
Ethmoid sinus
Maxillary sinus
Which of the paranasal sinuses is the largest?
Maxillary Sinuses
Sphenoid sinus
Tears are produced from parasympathetic innervation of the lacrimal gland.
Preganglionic fibres in the greater petrosal nerve (branch of CNVII) synapse at the pterygopalatine ganglion. Postganglionic fibres travel with the maxillary nerve then the zygomatic to then stimulate the secretion of tears.

## Slide 29
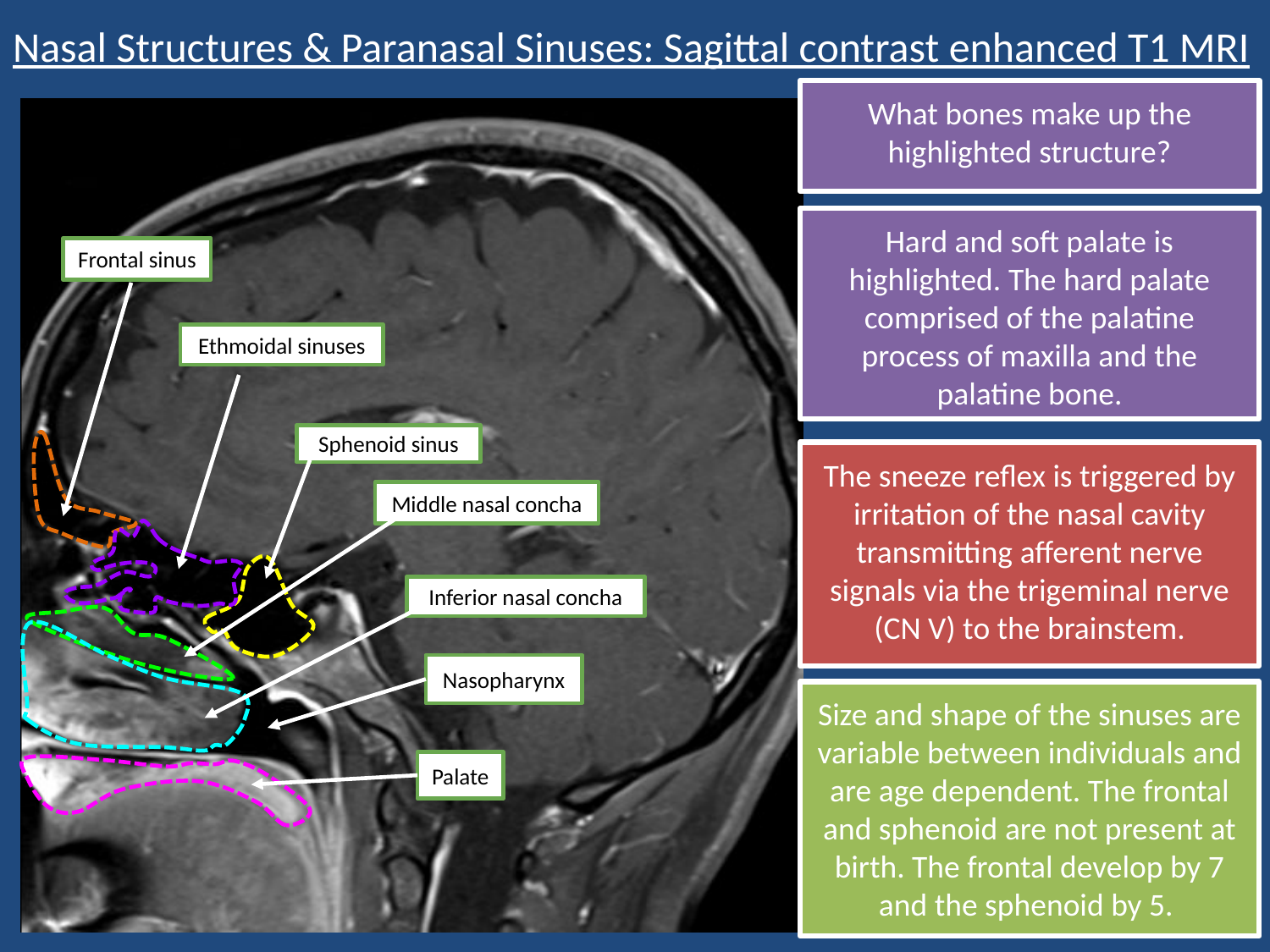

Nasal Structures & Paranasal Sinuses: Sagittal contrast enhanced T1 MRI
What bones make up the highlighted structure?
Hard and soft palate is highlighted. The hard palate comprised of the palatine process of maxilla and the palatine bone.
Frontal sinus
Ethmoidal sinuses
Sphenoid sinus
The sneeze reflex is triggered by irritation of the nasal cavity transmitting afferent nerve signals via the trigeminal nerve (CN V) to the brainstem.
Middle nasal concha
Inferior nasal concha
Nasopharynx
Size and shape of the sinuses are variable between individuals and are age dependent. The frontal and sphenoid are not present at birth. The frontal develop by 7 and the sphenoid by 5.
Palate

## Slide 30
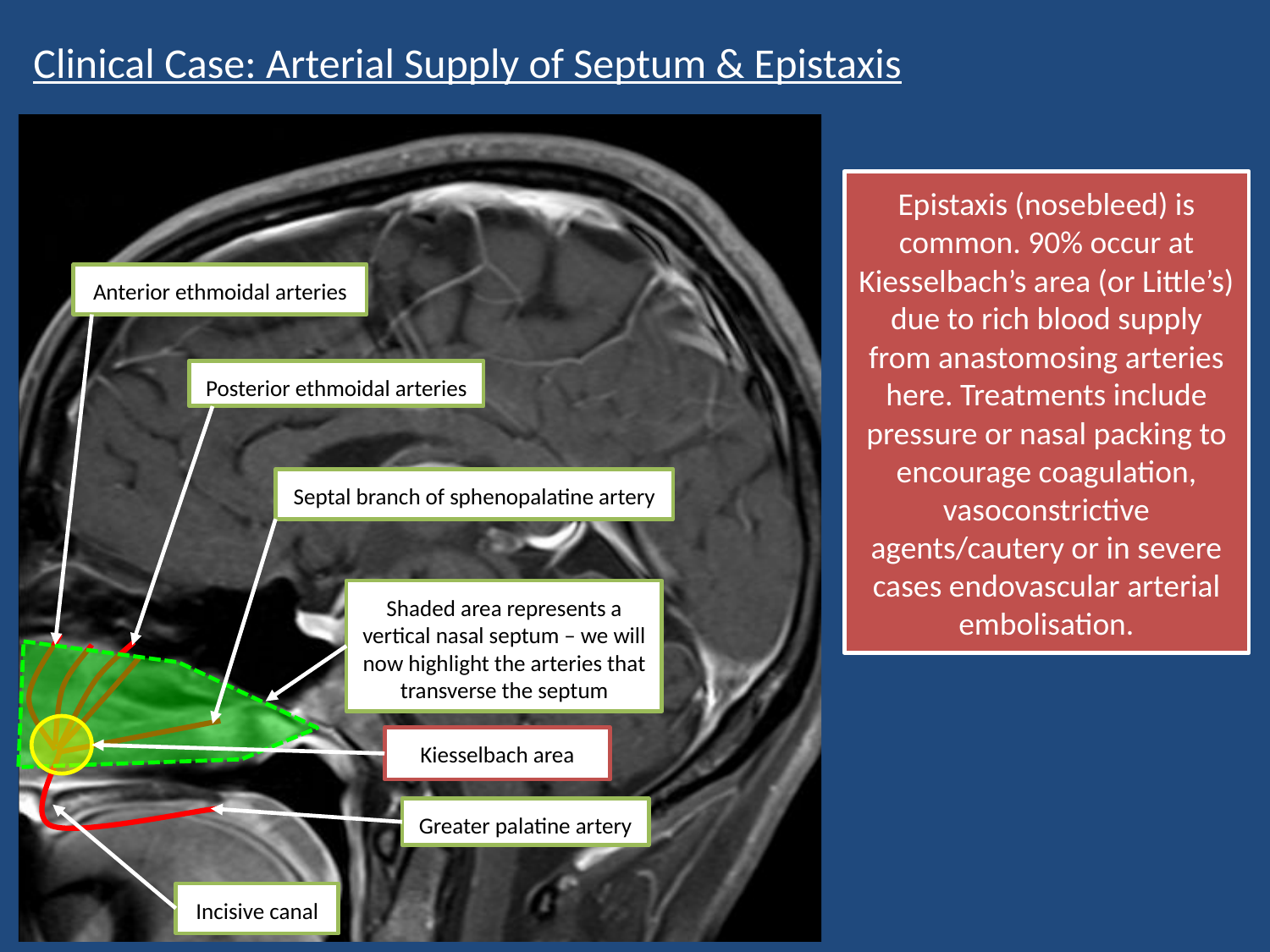

Clinical Case: Arterial Supply of Septum & Epistaxis
Epistaxis (nosebleed) is common. 90% occur at Kiesselbach’s area (or Little’s) due to rich blood supply from anastomosing arteries here. Treatments include pressure or nasal packing to encourage coagulation, vasoconstrictive agents/cautery or in severe cases endovascular arterial embolisation.
Anterior ethmoidal arteries
Posterior ethmoidal arteries
Septal branch of sphenopalatine artery
Shaded area represents a vertical nasal septum – we will now highlight the arteries that transverse the septum
Kiesselbach area
Greater palatine artery
Incisive canal

## Slide 31
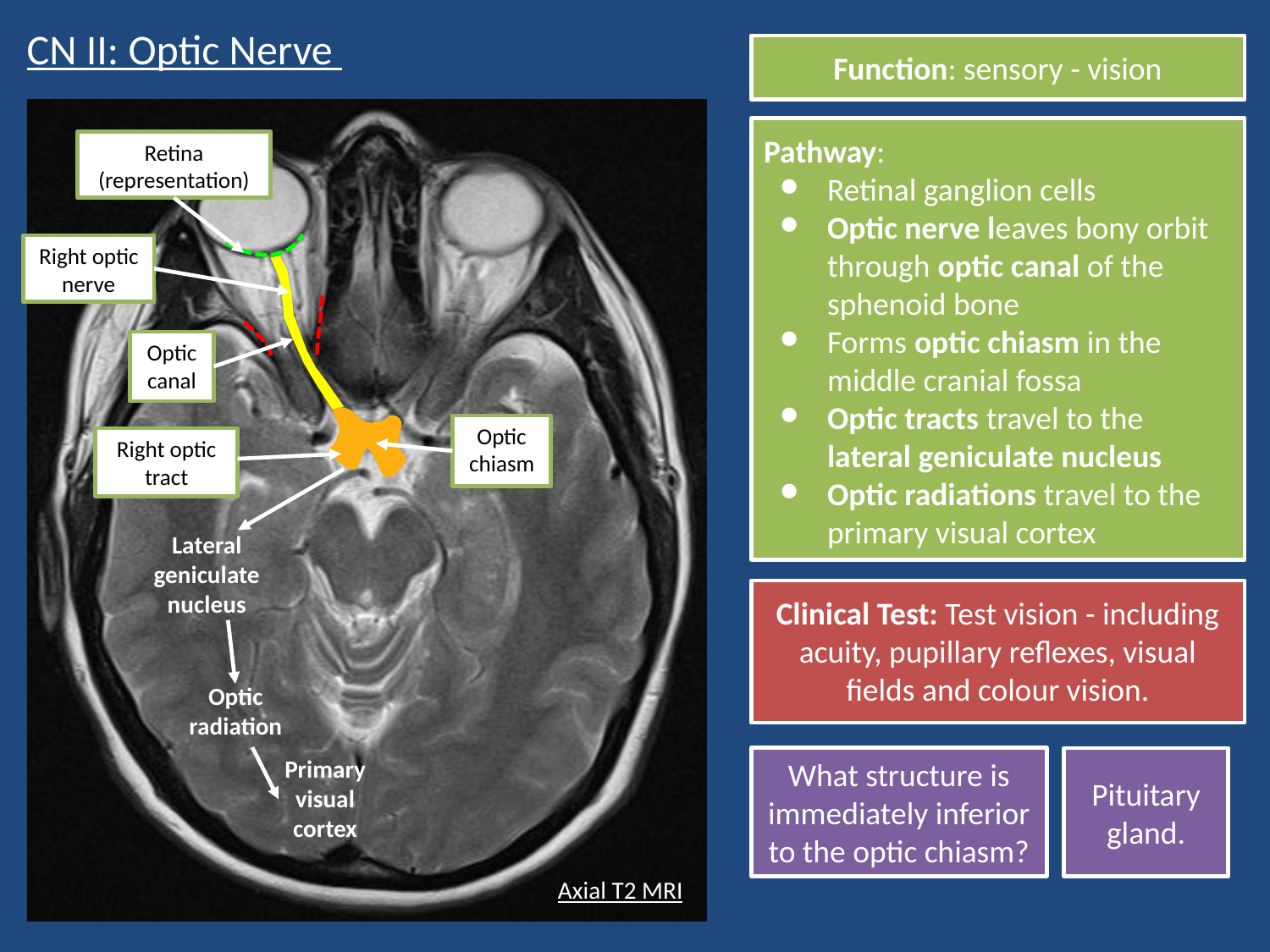

CN II: Optic Nerve
Function: sensory - vision
Pathway:
Retinal ganglion cells
Optic nerve leaves bony orbit through optic canal of the sphenoid bone
Forms optic chiasm in the middle cranial fossa
Optic tracts travel to the lateral geniculate nucleus
Optic radiations travel to the primary visual cortex
Retina (representation)
Right optic nerve
Optic canal
Optic chiasm
Right optic tract
Lateral geniculate nucleus
Clinical Test: Test vision - including acuity, pupillary reflexes, visual fields and colour vision.
Optic radiation
Primary visual cortex
What structure is immediately inferior to the optic chiasm?
Pituitary gland.
Axial T2 MRI

## Slide 32
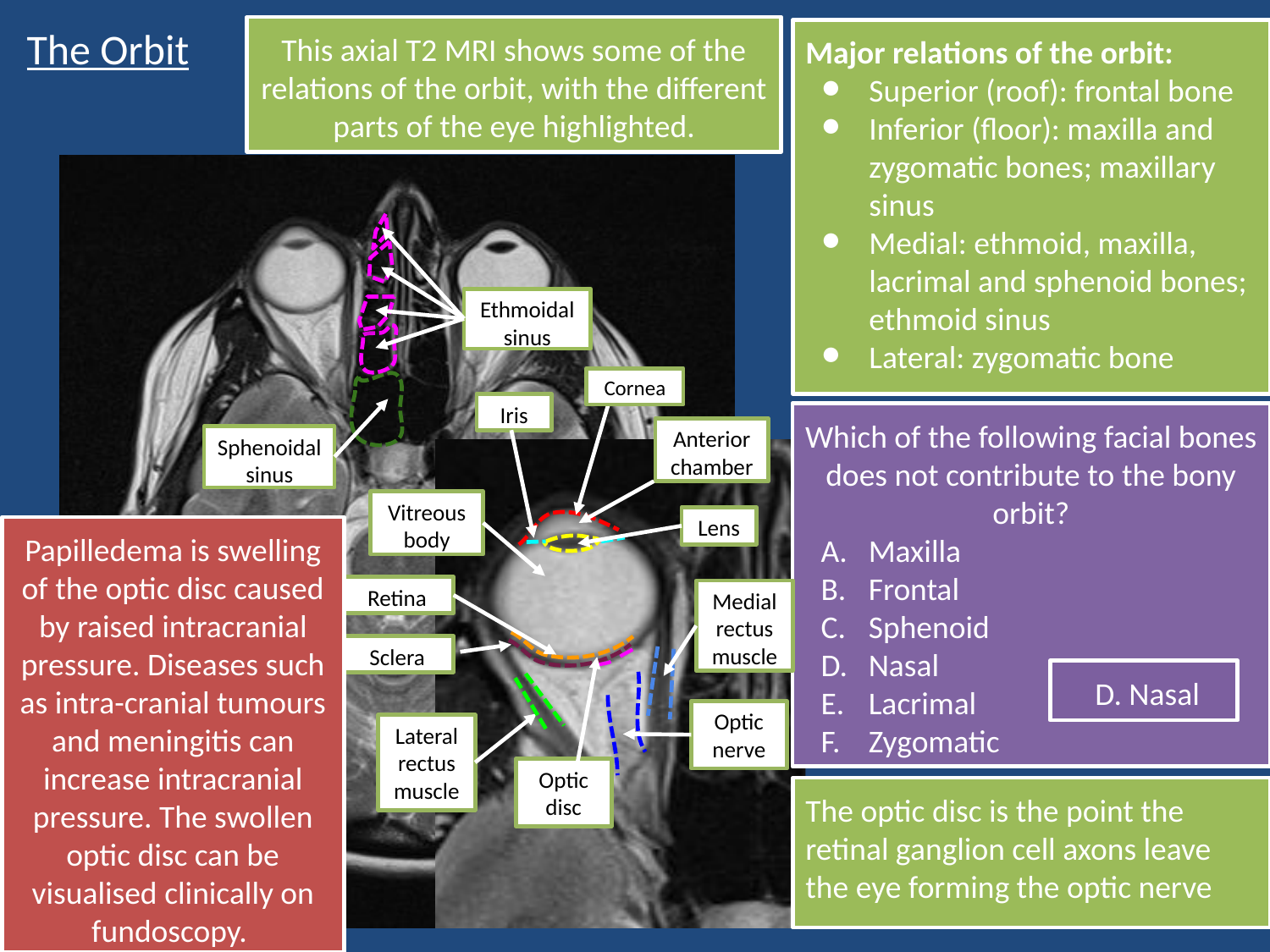

The Orbit
This axial T2 MRI shows some of the relations of the orbit, with the different parts of the eye highlighted.
Major relations of the orbit:
Superior (roof): frontal bone
Inferior (floor): maxilla and zygomatic bones; maxillary sinus
Medial: ethmoid, maxilla, lacrimal and sphenoid bones; ethmoid sinus
Lateral: zygomatic bone
Ethmoidal sinus
Cornea
Iris
Sphenoidal sinus
Which of the following facial bones does not contribute to the bony orbit?
Maxilla
Frontal
Sphenoid
Nasal
Lacrimal
Zygomatic
Anterior chamber
Vitreous body
Lens
Papilledema is swelling of the optic disc caused by raised intracranial pressure. Diseases such as intra-cranial tumours and meningitis can increase intracranial pressure. The swollen optic disc can be visualised clinically on fundoscopy.
Retina
Medial rectus muscle
Sclera
 D. Nasal
Optic disc
Optic nerve
Lateral rectus muscle
The optic disc is the point the retinal ganglion cell axons leave the eye forming the optic nerve

## Slide 33
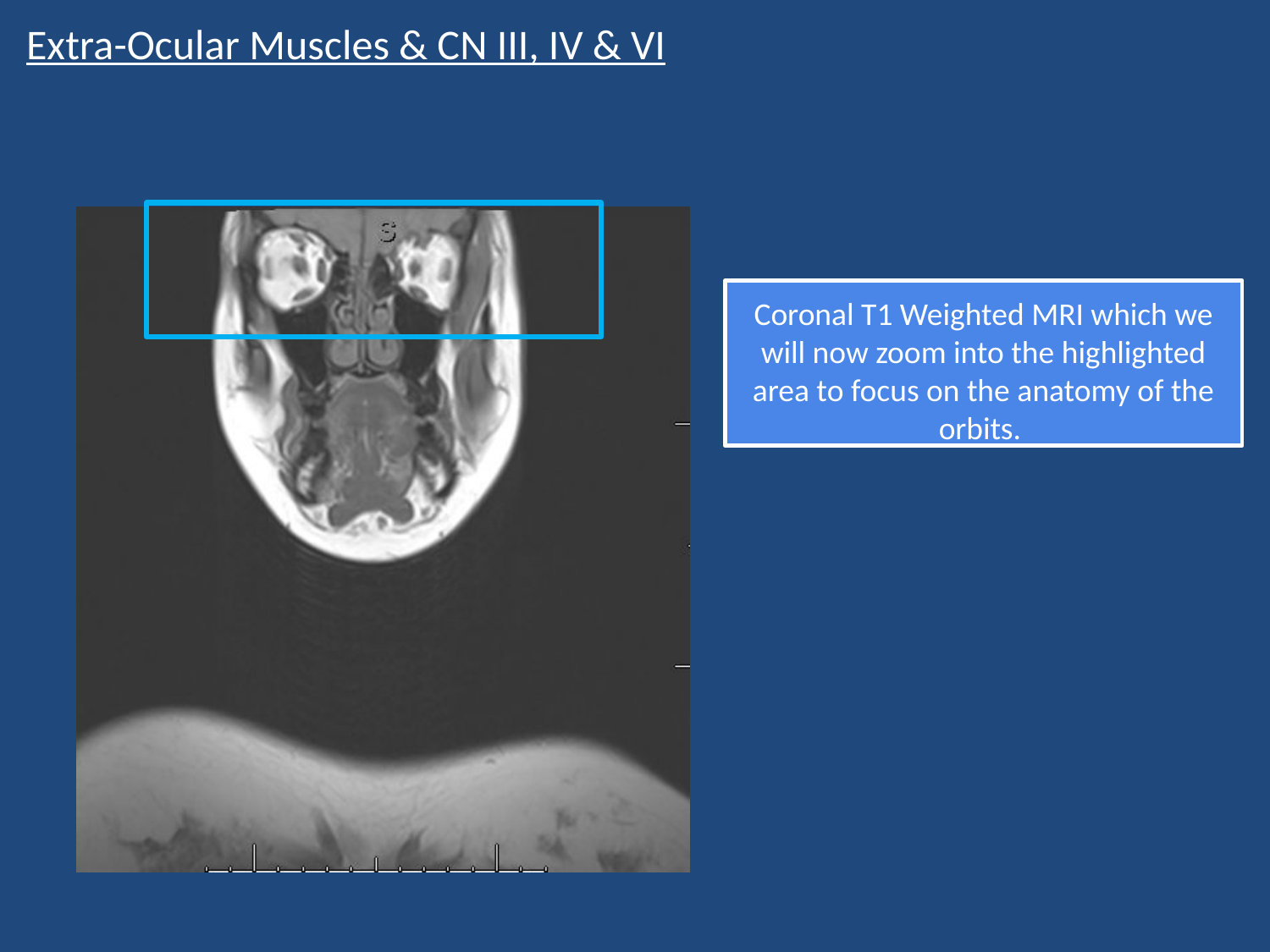

Extra-Ocular Muscles & CN III, IV & VI
Coronal T1 Weighted MRI which we will now zoom into the highlighted area to focus on the anatomy of the orbits.

## Slide 34
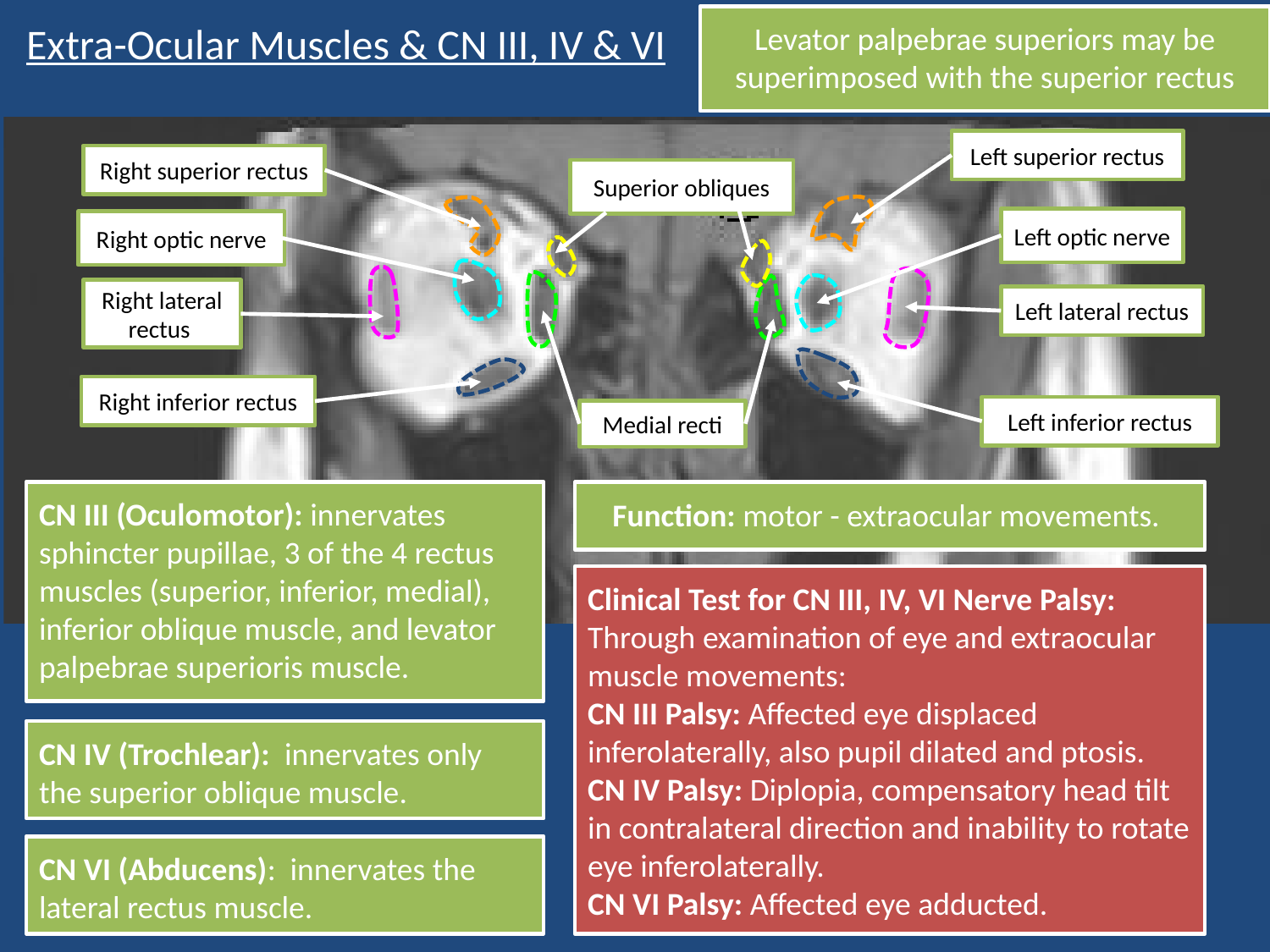

Extra-Ocular Muscles & CN III, IV & VI
Levator palpebrae superiors may be superimposed with the superior rectus
Left superior rectus
Right superior rectus
Superior obliques
Left optic nerve
Right optic nerve
Right lateral rectus
Left lateral rectus
Medial recti
Right inferior rectus
Left inferior rectus
CN III (Oculomotor): innervates sphincter pupillae, 3 of the 4 rectus muscles (superior, inferior, medial), inferior oblique muscle, and levator palpebrae superioris muscle.
Function: motor - extraocular movements.
Clinical Test for CN III, IV, VI Nerve Palsy:
Through examination of eye and extraocular muscle movements:
CN III Palsy: Affected eye displaced inferolaterally, also pupil dilated and ptosis.
CN IV Palsy: Diplopia, compensatory head tilt in contralateral direction and inability to rotate eye inferolaterally.
CN VI Palsy: Affected eye adducted.
CN IV (Trochlear): innervates only the superior oblique muscle.
CN VI (Abducens): innervates the lateral rectus muscle.

## Slide 35
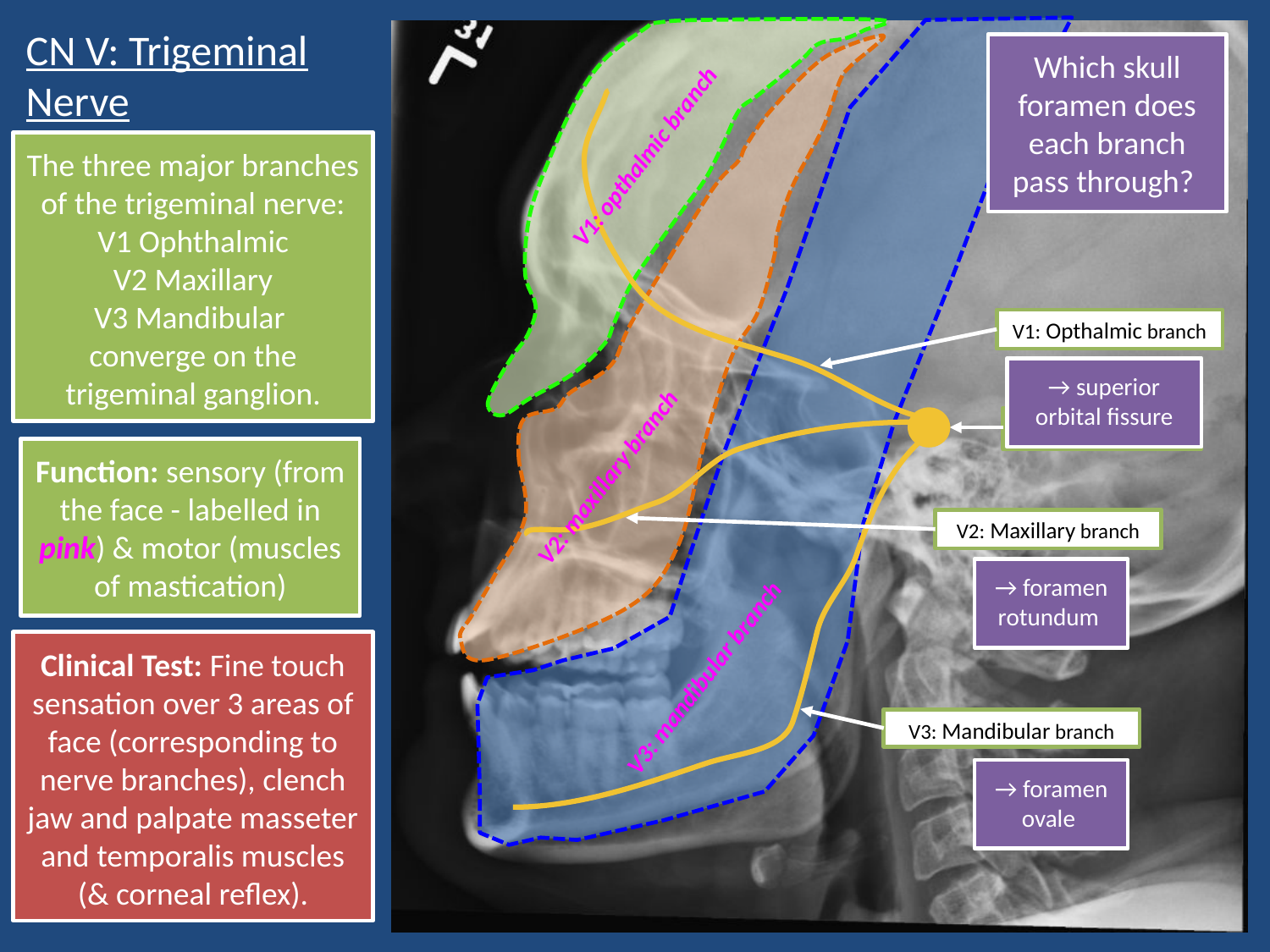

CN V: Trigeminal Nerve
Which skull foramen does each branch pass through?
V1: opthalmic branch
The three major branches of the trigeminal nerve: V1 Ophthalmic
V2 Maxillary
V3 Mandibular
converge on the trigeminal ganglion.
V1: Opthalmic branch
→ superior orbital fissure
V2: maxillary branch
Trigeminal ganglion
Function: sensory (from the face - labelled in pink) & motor (muscles of mastication)
V2: Maxillary branch
→ foramen rotundum
V3: mandibular branch
Clinical Test: Fine touch sensation over 3 areas of face (corresponding to nerve branches), clench jaw and palpate masseter and temporalis muscles (& corneal reflex).
V3: Mandibular branch
→ foramen ovale

## Slide 36
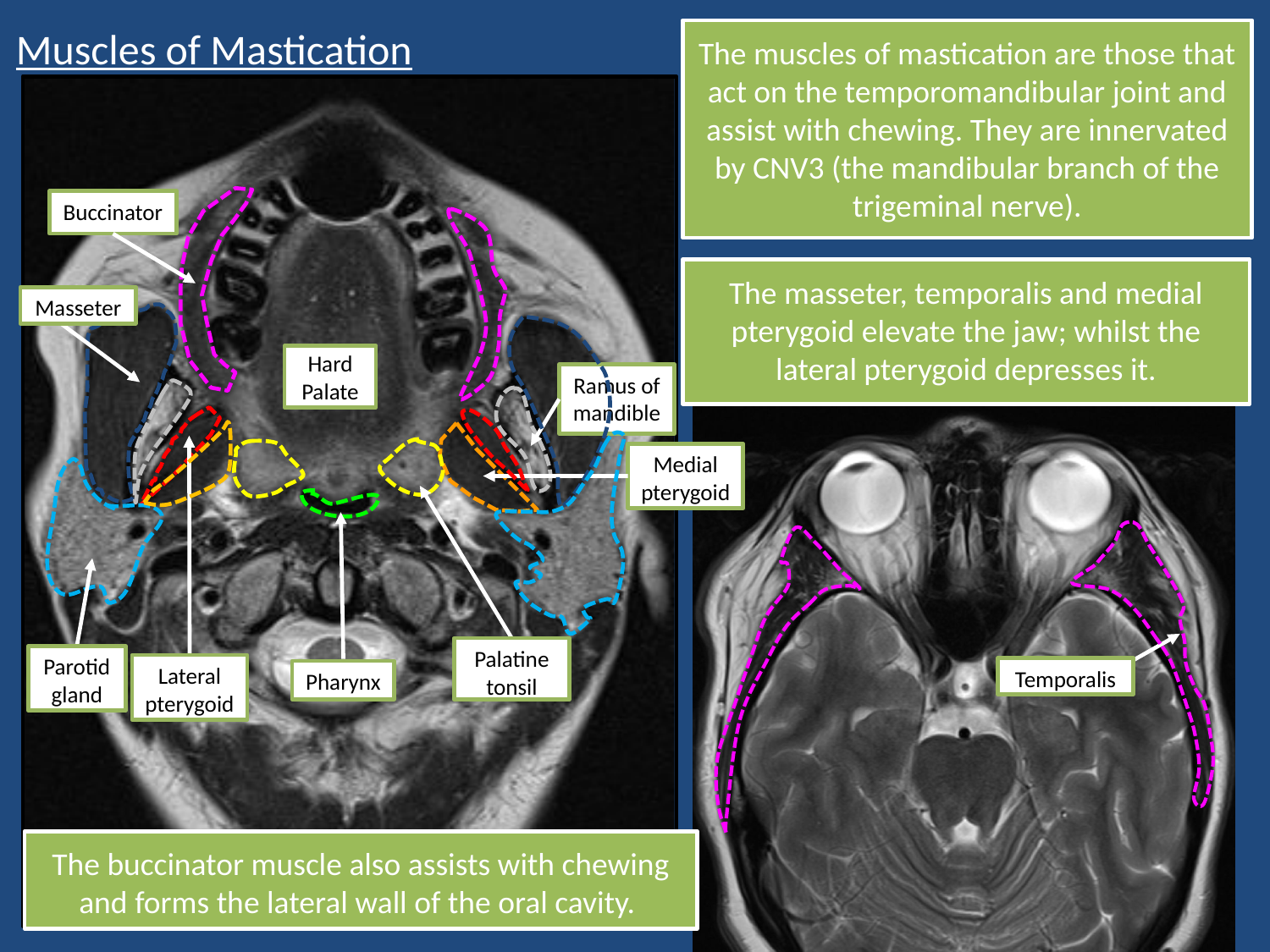

Muscles of Mastication
The muscles of mastication are those that act on the temporomandibular joint and assist with chewing. They are innervated by CNV3 (the mandibular branch of the trigeminal nerve).
Buccinator
The masseter, temporalis and medial pterygoid elevate the jaw; whilst the lateral pterygoid depresses it.
Masseter
Hard Palate
Ramus of mandible
Lateral pterygoid
Medial pterygoid
Palatine tonsil
Pharynx
Parotid gland
Temporalis
The buccinator muscle also assists with chewing and forms the lateral wall of the oral cavity.

## Slide 37
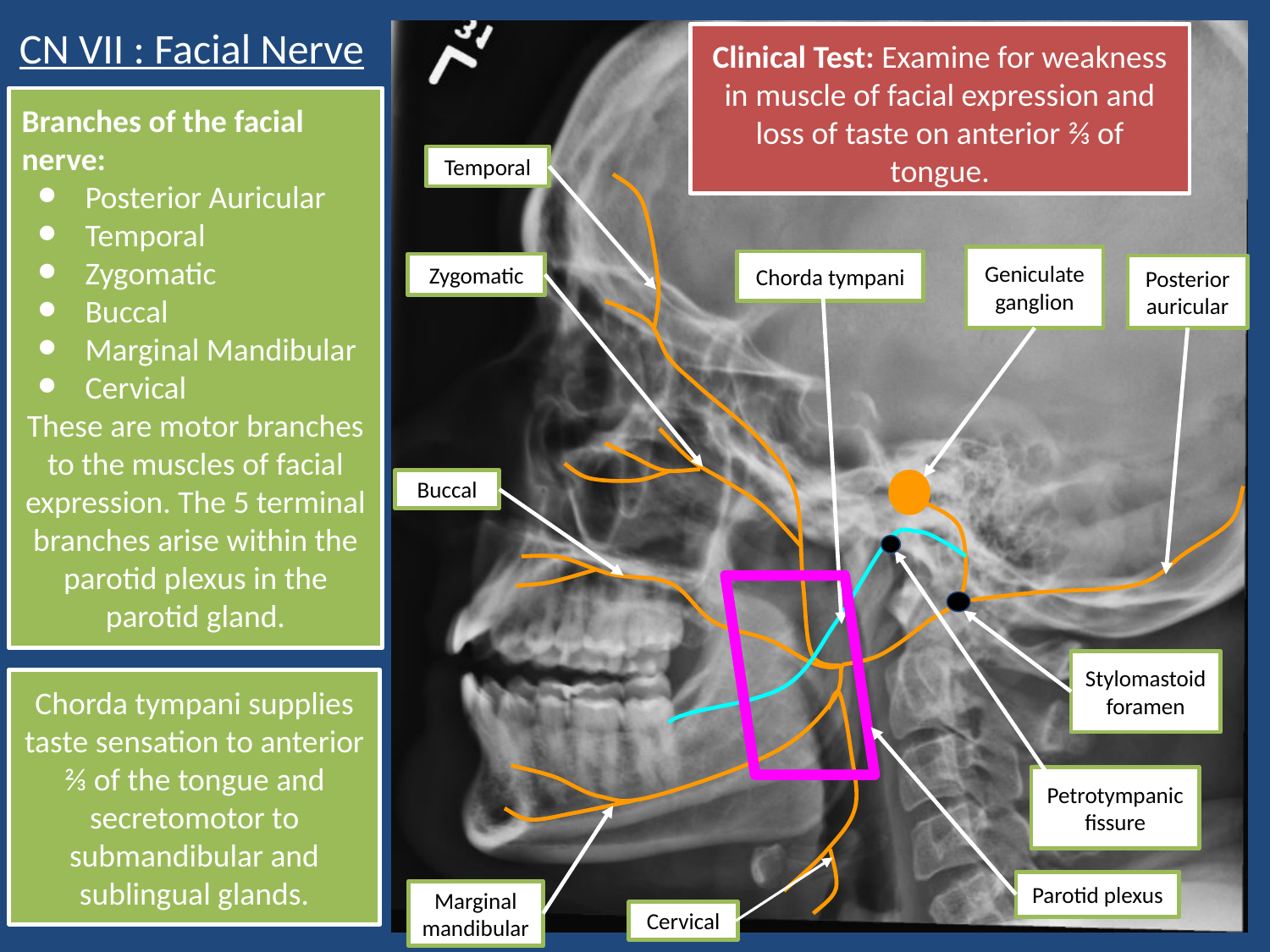

CN VII : Facial Nerve
Clinical Test: Examine for weakness in muscle of facial expression and loss of taste on anterior ⅔ of tongue.
The somatic motor branches of facial nerve exits the cranium via the stylomastoid foramen of the petrous temporal bone.
Branches of the facial nerve:
Posterior Auricular
Temporal
Zygomatic
Buccal
Marginal Mandibular
Cervical
These are motor branches to the muscles of facial expression. The 5 terminal branches arise within the parotid plexus in the parotid gland.
Temporal
Geniculate ganglion
Chorda tympani
Zygomatic
Posterior auricular
Buccal
Stylomastoid foramen
Chorda tympani supplies taste sensation to anterior ⅔ of the tongue and secretomotor to submandibular and sublingual glands.
Petrotympanic fissure
Parotid plexus
Marginal mandibular
Cervical

## Slide 38
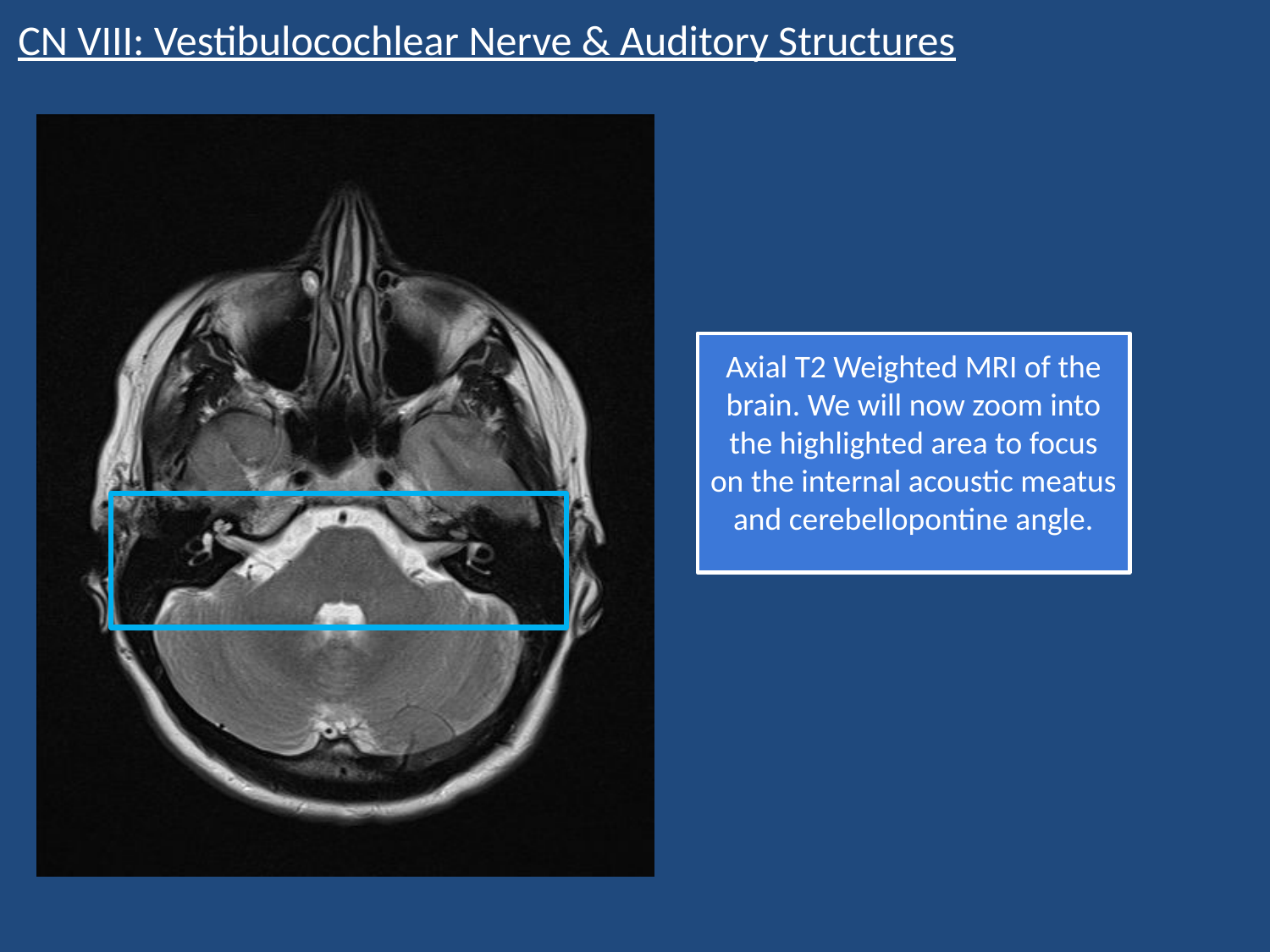

CN VIII: Vestibulocochlear Nerve & Auditory Structures
Axial T2 Weighted MRI of the brain. We will now zoom into the highlighted area to focus on the internal acoustic meatus and cerebellopontine angle.

## Slide 39
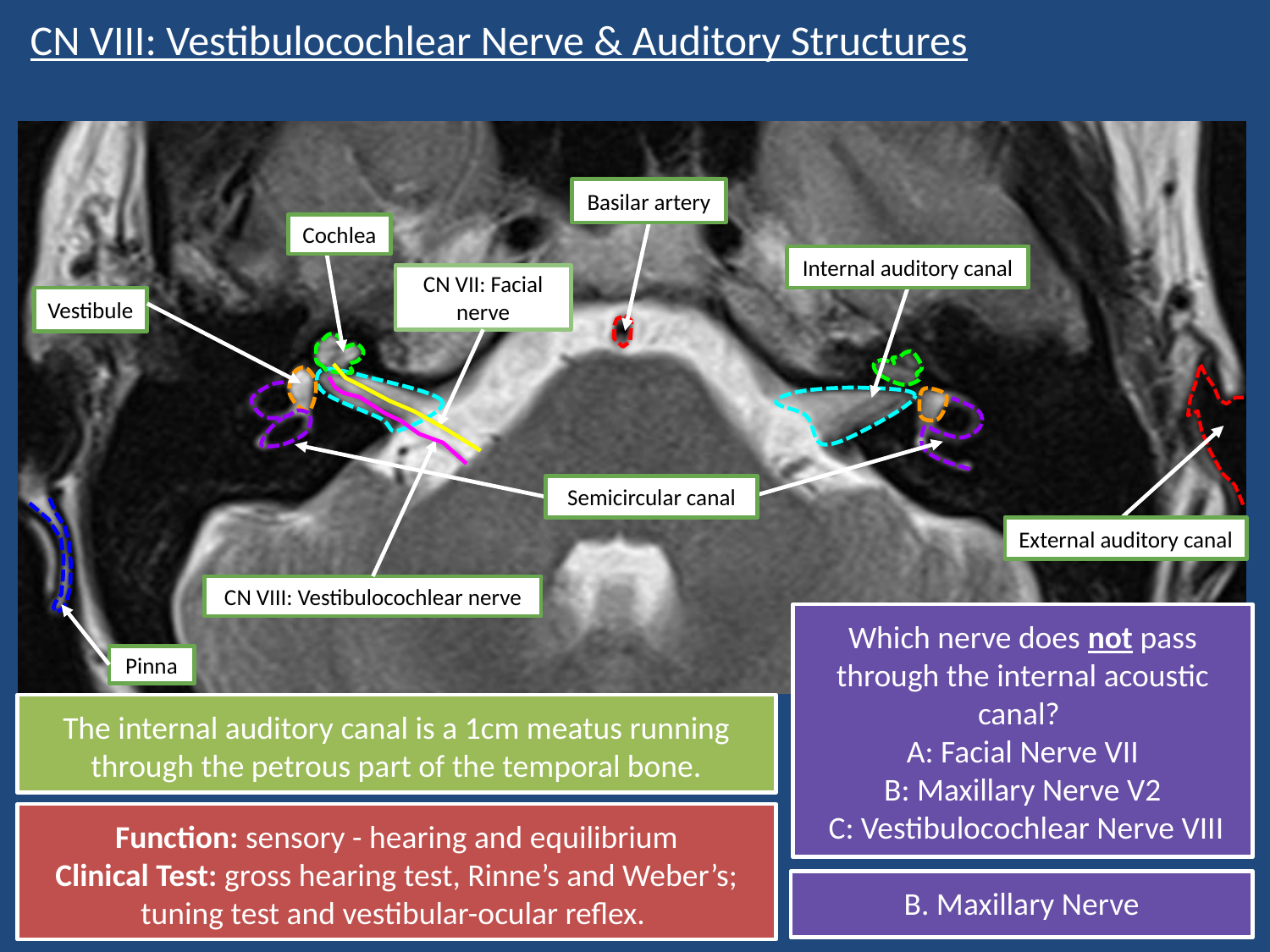

CN VIII: Vestibulocochlear Nerve & Auditory Structures
Basilar artery
Cochlea
Internal auditory canal
CN VII: Facial nerve
Vestibule
External auditory canal
CN VIII: Vestibulocochlear nerve
Semicircular canal
Which nerve does not pass through the internal acoustic canal?
A: Facial Nerve VII
B: Maxillary Nerve V2
 C: Vestibulocochlear Nerve VIII
Pinna
The internal auditory canal is a 1cm meatus running through the petrous part of the temporal bone.
Function: sensory - hearing and equilibrium
Clinical Test: gross hearing test, Rinne’s and Weber’s; tuning test and vestibular-ocular reflex.
B. Maxillary Nerve

## Slide 40
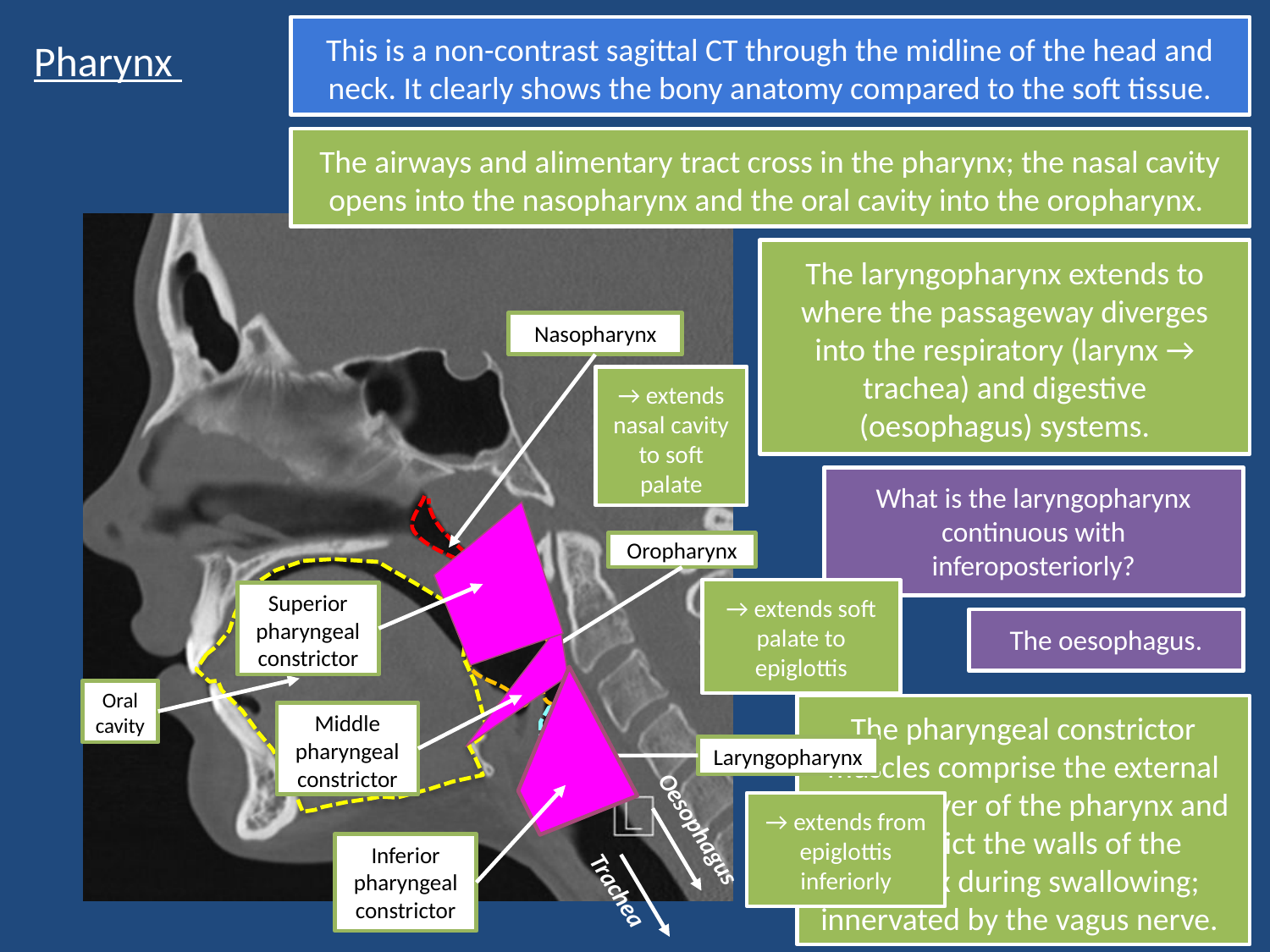

This is a non-contrast sagittal CT through the midline of the head and neck. It clearly shows the bony anatomy compared to the soft tissue.
Pharynx
The airways and alimentary tract cross in the pharynx; the nasal cavity opens into the nasopharynx and the oral cavity into the oropharynx.
The laryngopharynx extends to where the passageway diverges into the respiratory (larynx → trachea) and digestive (oesophagus) systems.
Nasopharynx
→ extends nasal cavity to soft palate
What is the laryngopharynx continuous with inferoposteriorly?
Oropharynx
→ extends soft palate to epiglottis
Superior pharyngeal constrictor
The oesophagus.
Oral cavity
Middle pharyngeal constrictor
The pharyngeal constrictor muscles comprise the external muscle layer of the pharynx and constrict the walls of the pharynx during swallowing; innervated by the vagus nerve.
Laryngopharynx
Oesophagus
Inferior pharyngeal constrictor
→ extends from epiglottis inferiorly
Trachea

## Slide 41
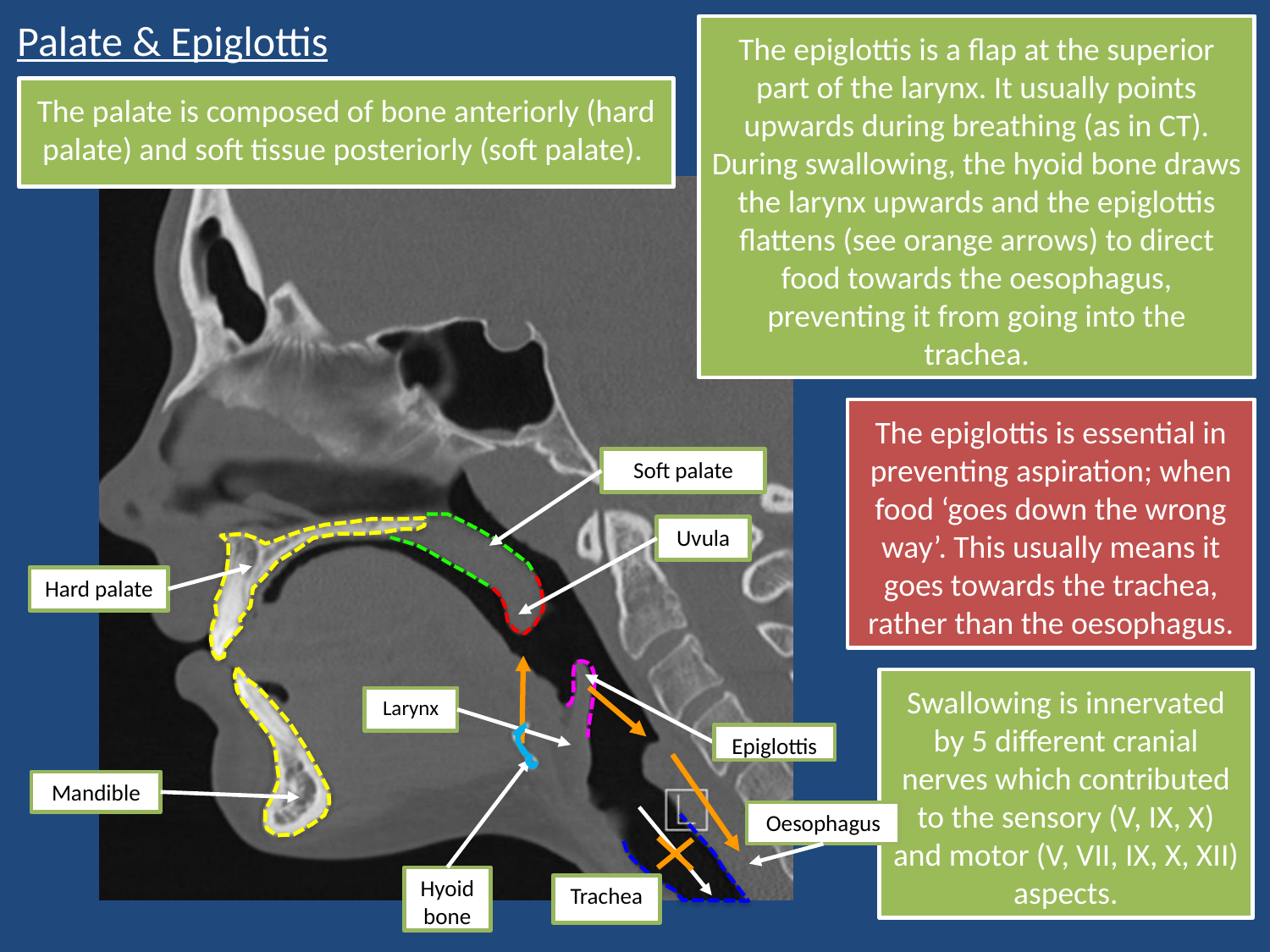

Palate & Epiglottis
The epiglottis is a flap at the superior part of the larynx. It usually points upwards during breathing (as in CT). During swallowing, the hyoid bone draws the larynx upwards and the epiglottis flattens (see orange arrows) to direct food towards the oesophagus, preventing it from going into the trachea.
The palate is composed of bone anteriorly (hard palate) and soft tissue posteriorly (soft palate).
The epiglottis is essential in preventing aspiration; when food ‘goes down the wrong way’. This usually means it goes towards the trachea, rather than the oesophagus.
Soft palate
Uvula
Hard palate
Swallowing is innervated by 5 different cranial nerves which contributed to the sensory (V, IX, X) and motor (V, VII, IX, X, XII) aspects.
Epiglottis
Larynx
Hyoid bone
Mandible
Oesophagus
Trachea

## Slide 42
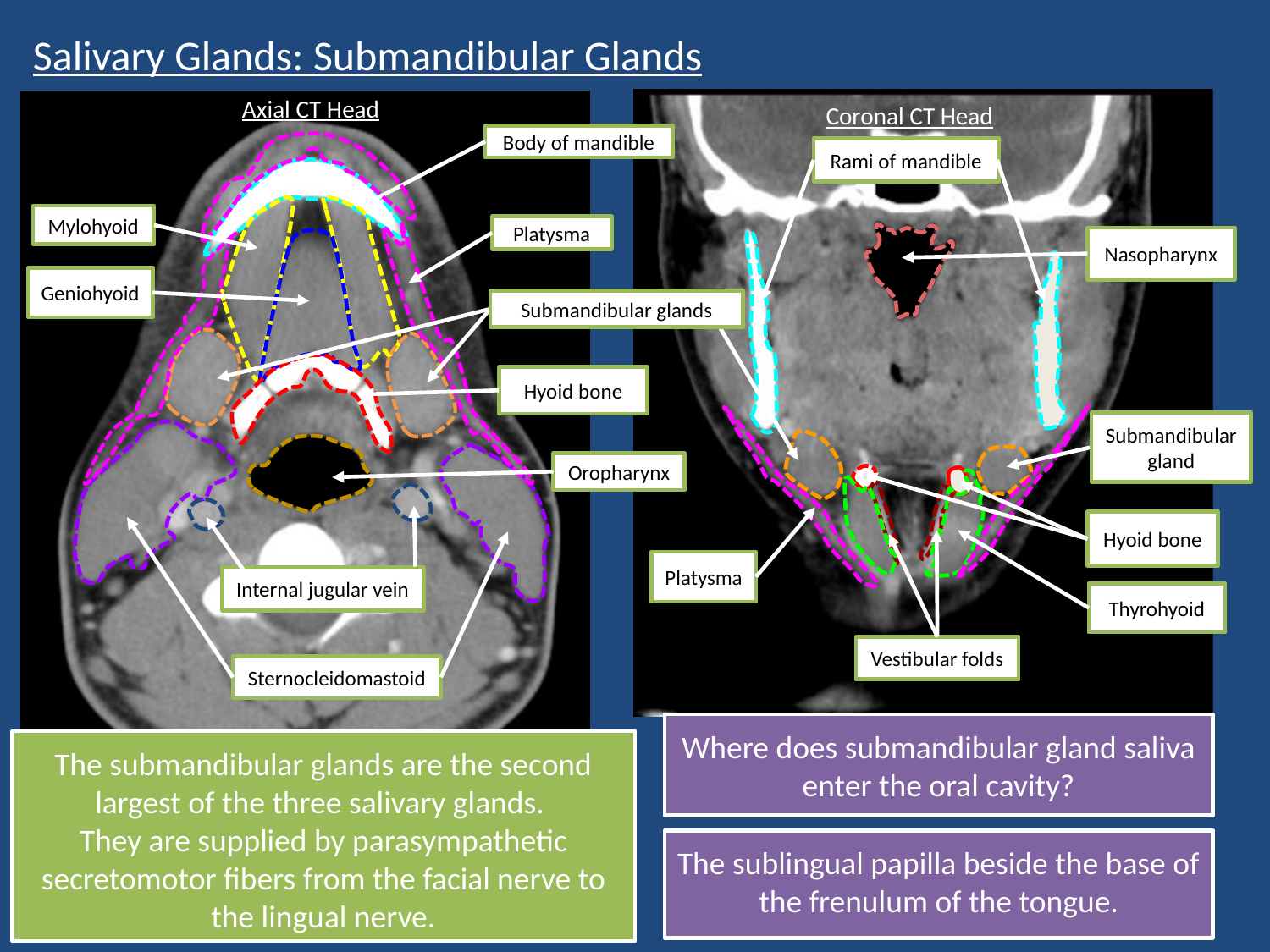

Salivary Glands: Submandibular Glands
Axial CT Head
Coronal CT Head
Body of mandible
Rami of mandible
Mylohyoid
Platysma
Platysma
Nasopharynx
Geniohyoid
Submandibular glands
Submandibular gland
Hyoid bone
Hyoid bone
Oropharynx
Internal jugular vein
Sternocleidomastoid
Thyrohyoid
Vestibular folds
Where does submandibular gland saliva enter the oral cavity?
The submandibular glands are the second largest of the three salivary glands.
They are supplied by parasympathetic secretomotor fibers from the facial nerve to the lingual nerve.
The sublingual papilla beside the base of the frenulum of the tongue.

## Slide 43
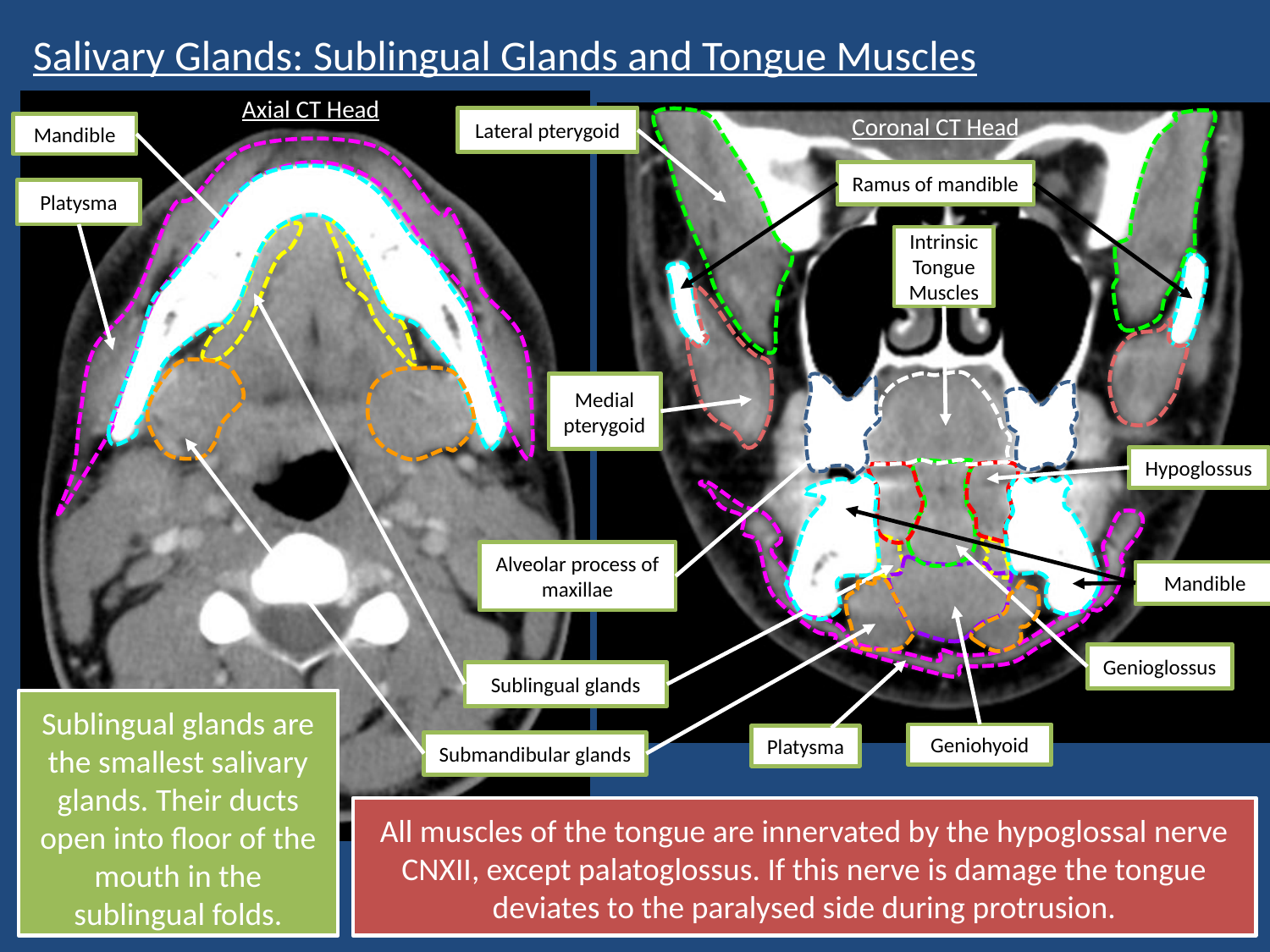

Salivary Glands: Sublingual Glands and Tongue Muscles
Axial CT Head
Coronal CT Head
Lateral pterygoid
Mandible
Mandible
Ramus of mandible
Platysma
Platysma
Intrinsic Tongue Muscles
Sublingual glands
Medial pterygoid
Submandibular glands
Alveolar process of maxillae
Hypoglossus
Genioglossus
Geniohyoid
Sublingual glands are the smallest salivary glands. Their ducts open into floor of the mouth in the sublingual folds.
All muscles of the tongue are innervated by the hypoglossal nerve CNXII, except palatoglossus. If this nerve is damage the tongue deviates to the paralysed side during protrusion.

## Slide 44
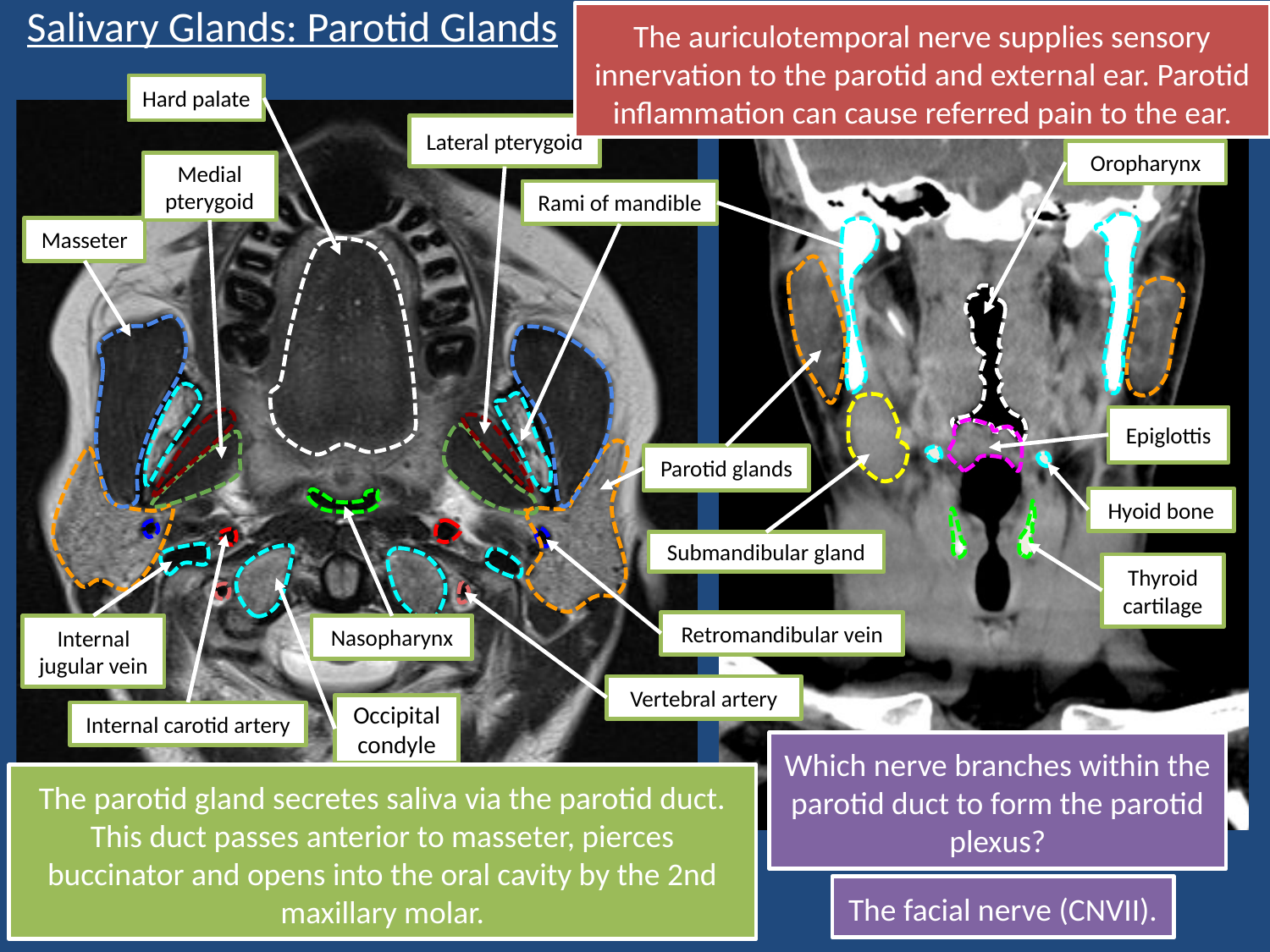

Salivary Glands: Parotid Glands
The auriculotemporal nerve supplies sensory innervation to the parotid and external ear. Parotid inflammation can cause referred pain to the ear.
Axial T1 Weighted MRI neck (left) and Coronal CT neck (right).
Hard palate
Lateral pterygoid
Oropharynx
Medial pterygoid
Rami of mandible
Masseter
Parotid glands
Epiglottis
Submandibular gland
Hyoid bone
Nasopharynx
Internal carotid artery
Retromandibular vein
Thyroid cartilage
Internal jugular vein
Occipital condyle
Vertebral artery
Which nerve branches within the parotid duct to form the parotid plexus?
The parotid gland secretes saliva via the parotid duct. This duct passes anterior to masseter, pierces buccinator and opens into the oral cavity by the 2nd maxillary molar.
The facial nerve (CNVII).

## Slide 45
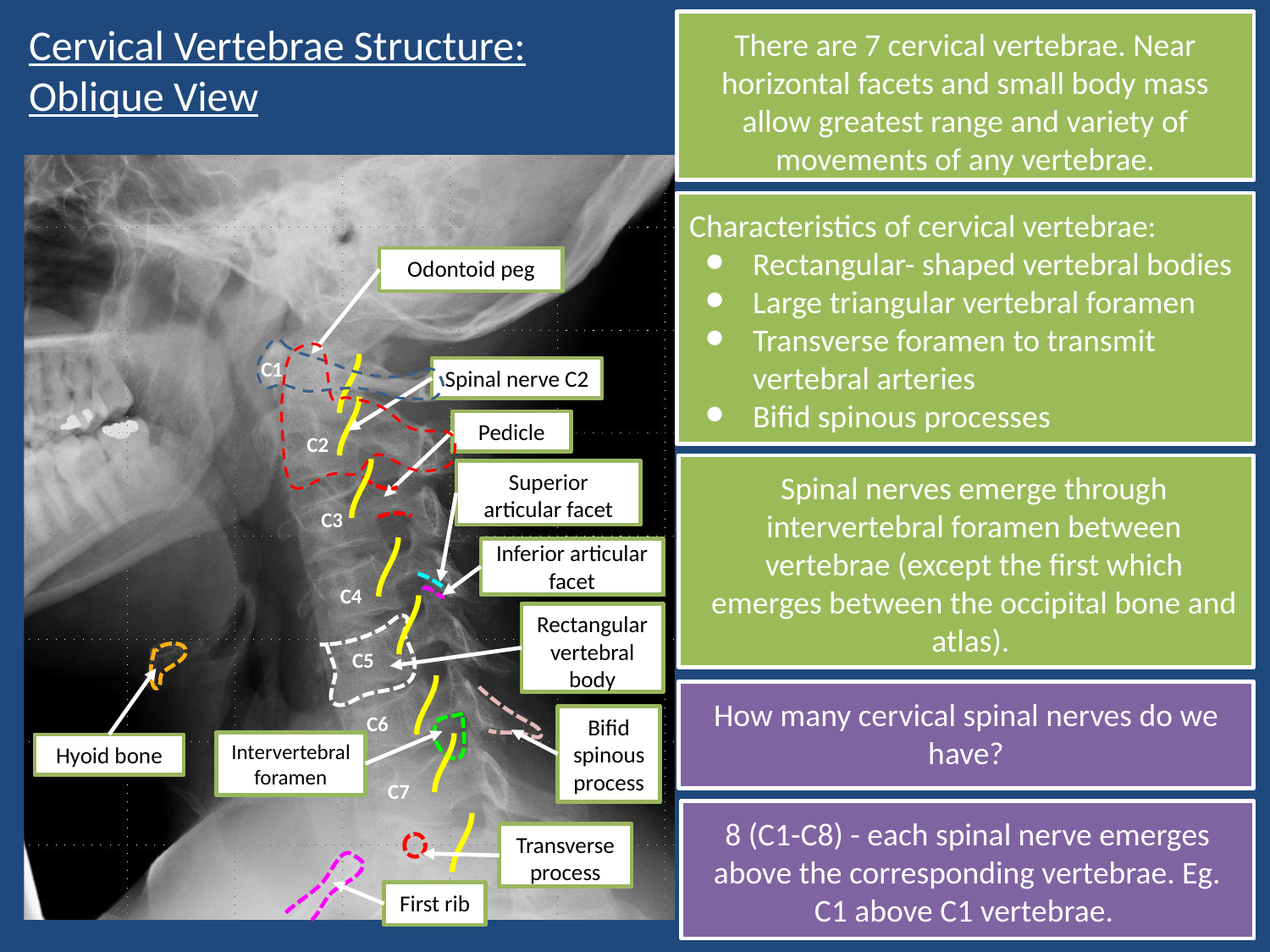

Cervical Vertebrae Structure: Oblique View
There are 7 cervical vertebrae. Near horizontal facets and small body mass allow greatest range and variety of movements of any vertebrae.
Characteristics of cervical vertebrae:
Rectangular- shaped vertebral bodies
Large triangular vertebral foramen
Transverse foramen to transmit vertebral arteries
Bifid spinous processes
Odontoid peg
C1
Spinal nerve C2
Pedicle
C2
Spinal nerves emerge through intervertebral foramen between vertebrae (except the first which emerges between the occipital bone and atlas).
Superior articular facet
C3
Inferior articular facet
C4
Rectangular vertebral body
C5
Hyoid bone
How many cervical spinal nerves do we have?
C6
Bifid spinous process
Intervertebral foramen
C7
8 (C1-C8) - each spinal nerve emerges above the corresponding vertebrae. Eg. C1 above C1 vertebrae.
Transverse process
First rib

## Slide 46
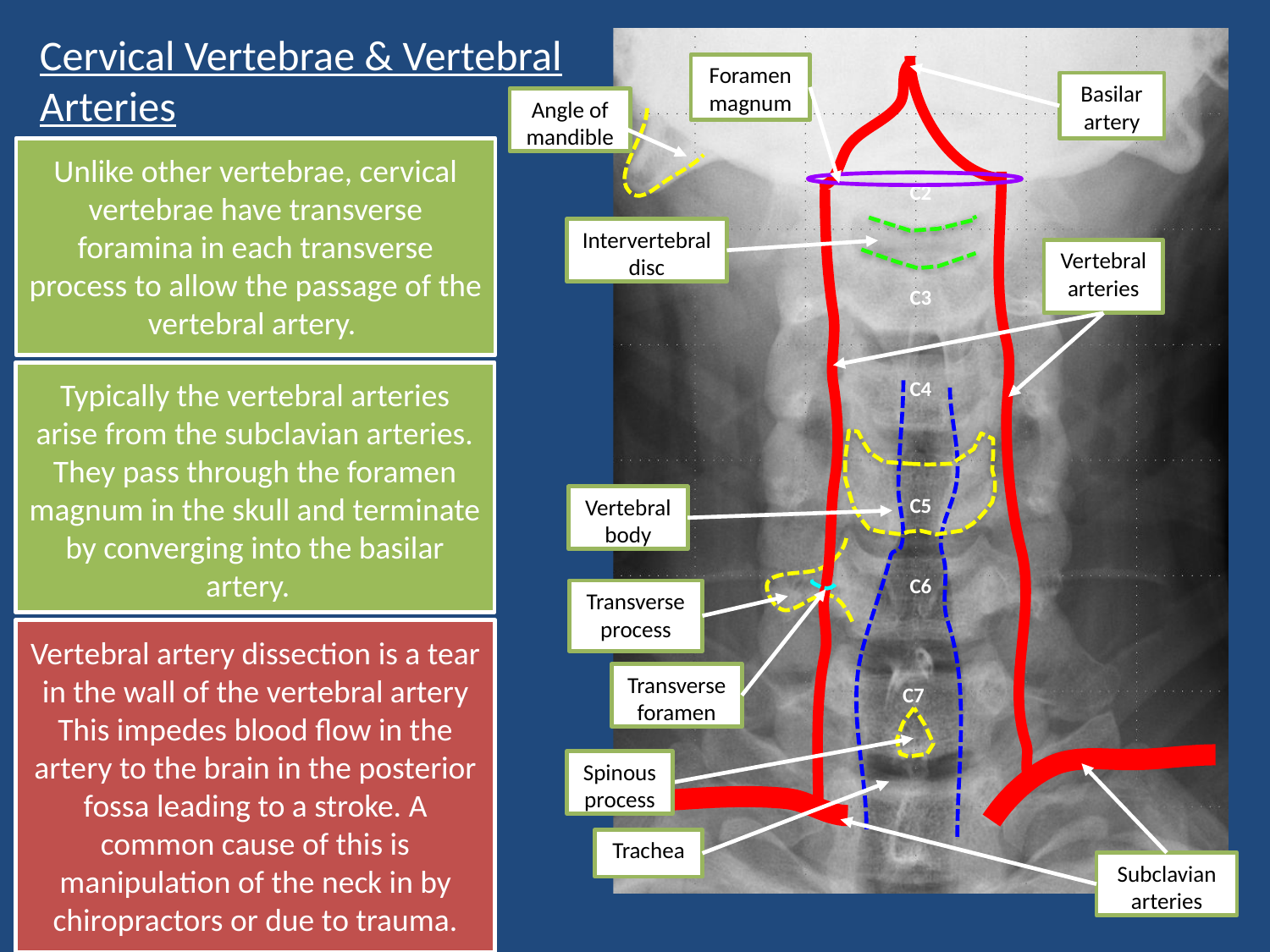

Cervical Vertebrae & Vertebral Arteries
Foramen magnum
Basilar artery
Angle of mandible
Unlike other vertebrae, cervical vertebrae have transverse foramina in each transverse process to allow the passage of the vertebral artery.
C2
Intervertebral disc
Vertebral arteries
C3
Typically the vertebral arteries arise from the subclavian arteries. They pass through the foramen magnum in the skull and terminate by converging into the basilar artery.
C4
C5
Vertebral body
C6
Transverse process
Transverse foramen
Vertebral artery dissection is a tear in the wall of the vertebral artery This impedes blood flow in the artery to the brain in the posterior fossa leading to a stroke. A common cause of this is manipulation of the neck in by chiropractors or due to trauma.
C7
Which branch of the subclavian arteries are the vertebral arteries?
Spinous process
Subclavian arteries
Trachea
The first branch

## Slide 47
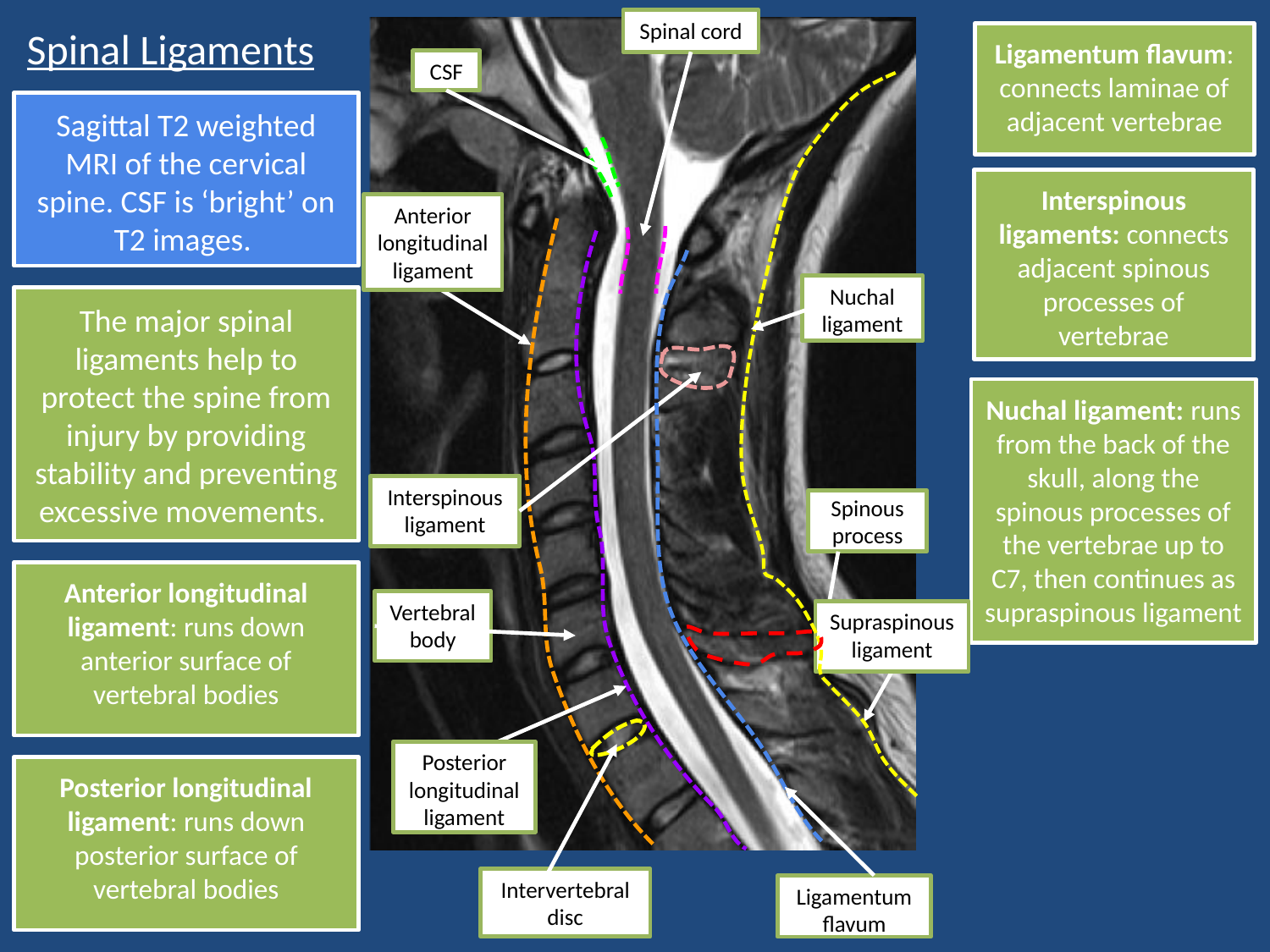

Spinal Ligaments
Spinal cord
Ligamentum flavum: connects laminae of adjacent vertebrae
CSF
Sagittal T2 weighted MRI of the cervical spine. CSF is ‘bright’ on T2 images.
Interspinous ligaments: connects adjacent spinous processes of vertebrae
Anterior longitudinal ligament
Nuchal ligament
The major spinal ligaments help to protect the spine from injury by providing stability and preventing excessive movements.
Interspinous ligament
Nuchal ligament: runs from the back of the skull, along the spinous processes of the vertebrae up to C7, then continues as supraspinous ligament
Spinous process
Anterior longitudinal ligament: runs down anterior surface of vertebral bodies
Vertebral body
Supraspinous ligament
Posterior longitudinal ligament
Intervertebral disc
Posterior longitudinal ligament: runs down posterior surface of vertebral bodies
Ligamentum flavum

## Slide 48
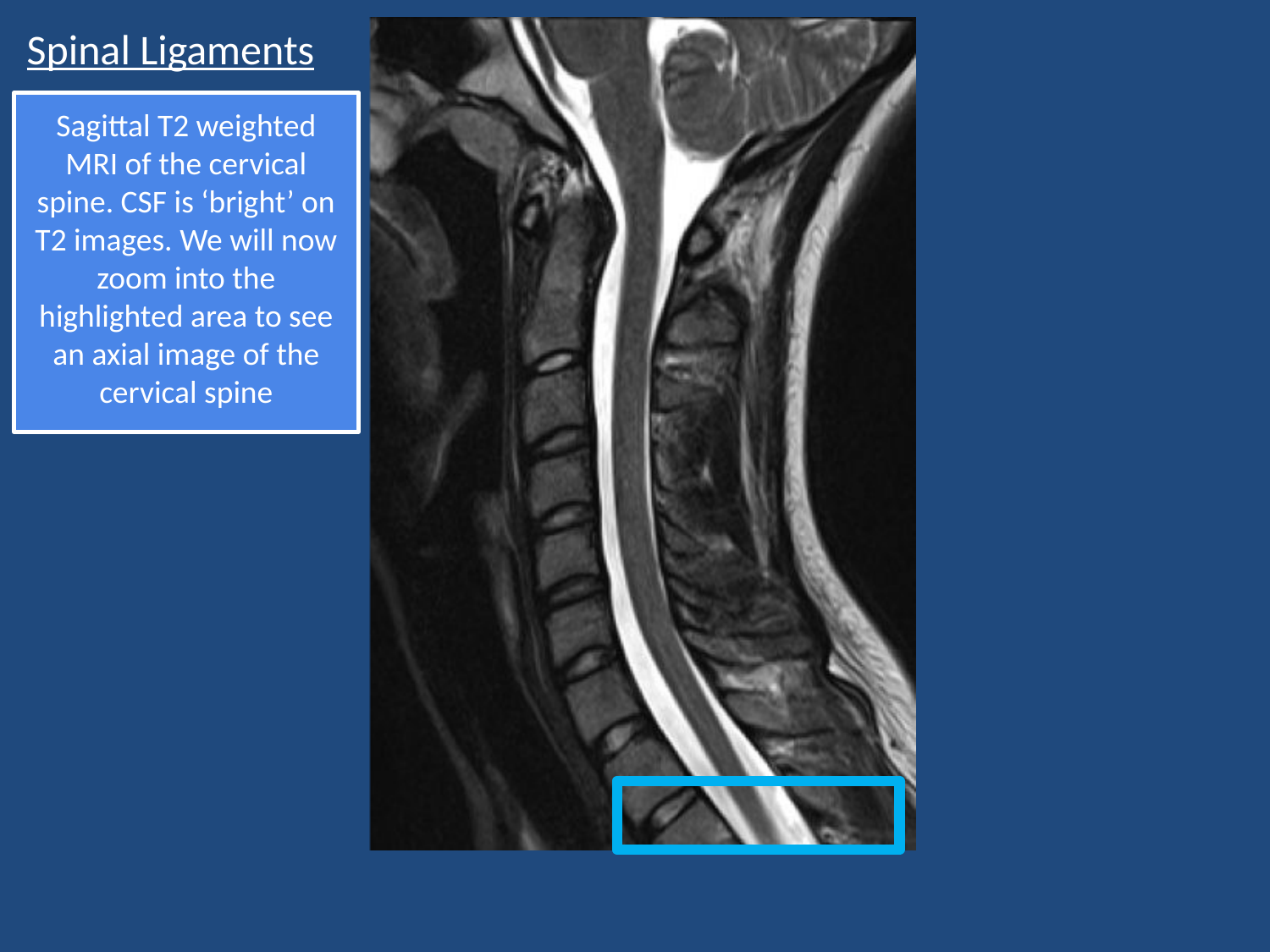

Spinal Ligaments
Sagittal T2 weighted MRI of the cervical spine. CSF is ‘bright’ on T2 images. We will now zoom into the highlighted area to see an axial image of the cervical spine

## Slide 49
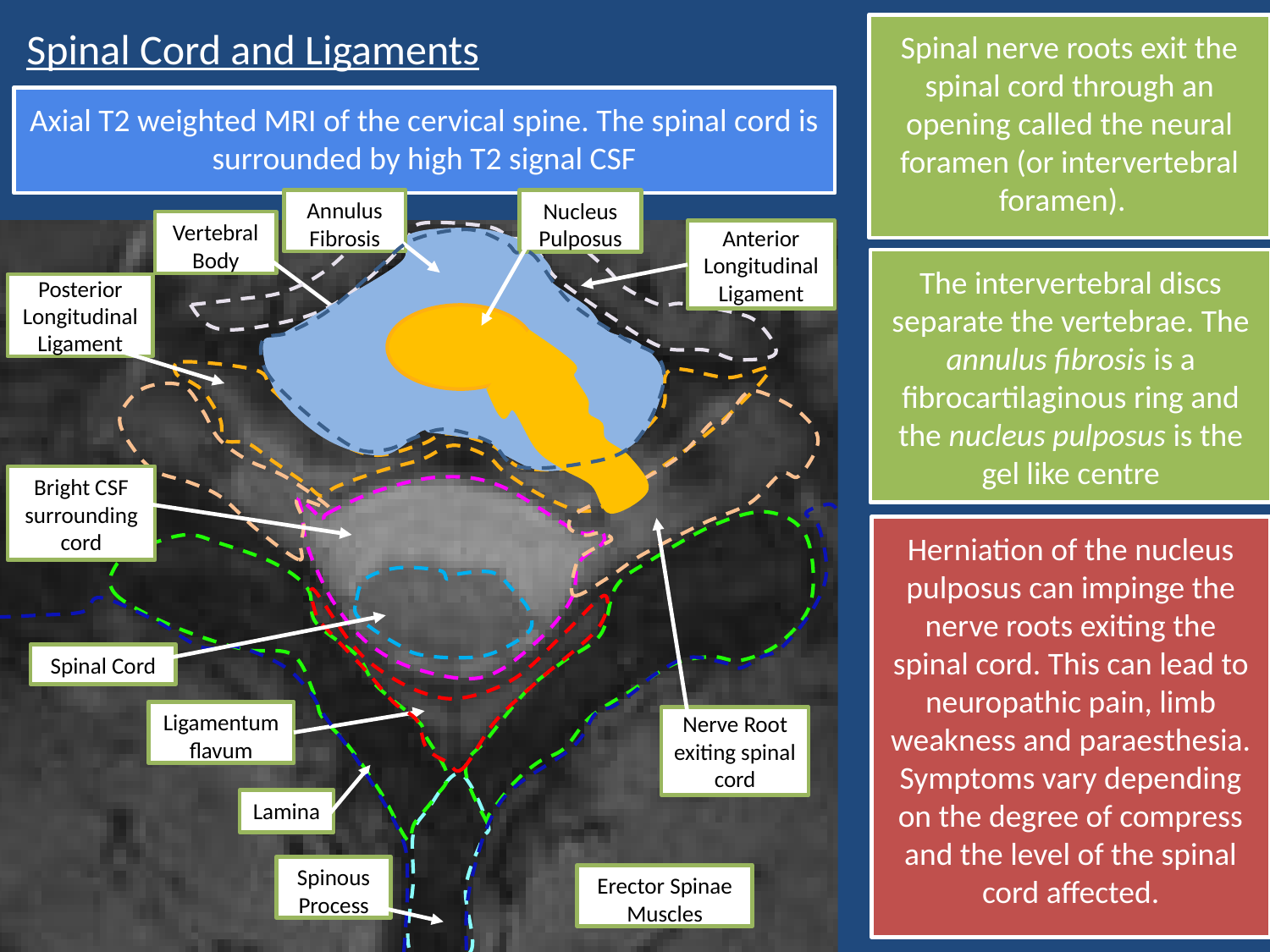

Spinal Cord and Ligaments
Spinal nerve roots exit the spinal cord through an opening called the neural foramen (or intervertebral foramen).
Axial T2 weighted MRI of the cervical spine. The spinal cord is surrounded by high T2 signal CSF
Annulus Fibrosis
Nucleus Pulposus
Vertebral Body
Anterior Longitudinal Ligament
The intervertebral discs separate the vertebrae. The annulus fibrosis is a fibrocartilaginous ring and the nucleus pulposus is the gel like centre
Posterior Longitudinal Ligament
Bright CSF surrounding cord
Herniation of the nucleus pulposus can impinge the nerve roots exiting the spinal cord. This can lead to neuropathic pain, limb weakness and paraesthesia. Symptoms vary depending on the degree of compress and the level of the spinal cord affected.
Spinal Cord
Ligamentum flavum
Nerve Root exiting spinal cord
Lamina
Spinous Process
Erector Spinae Muscles

## Slide 50
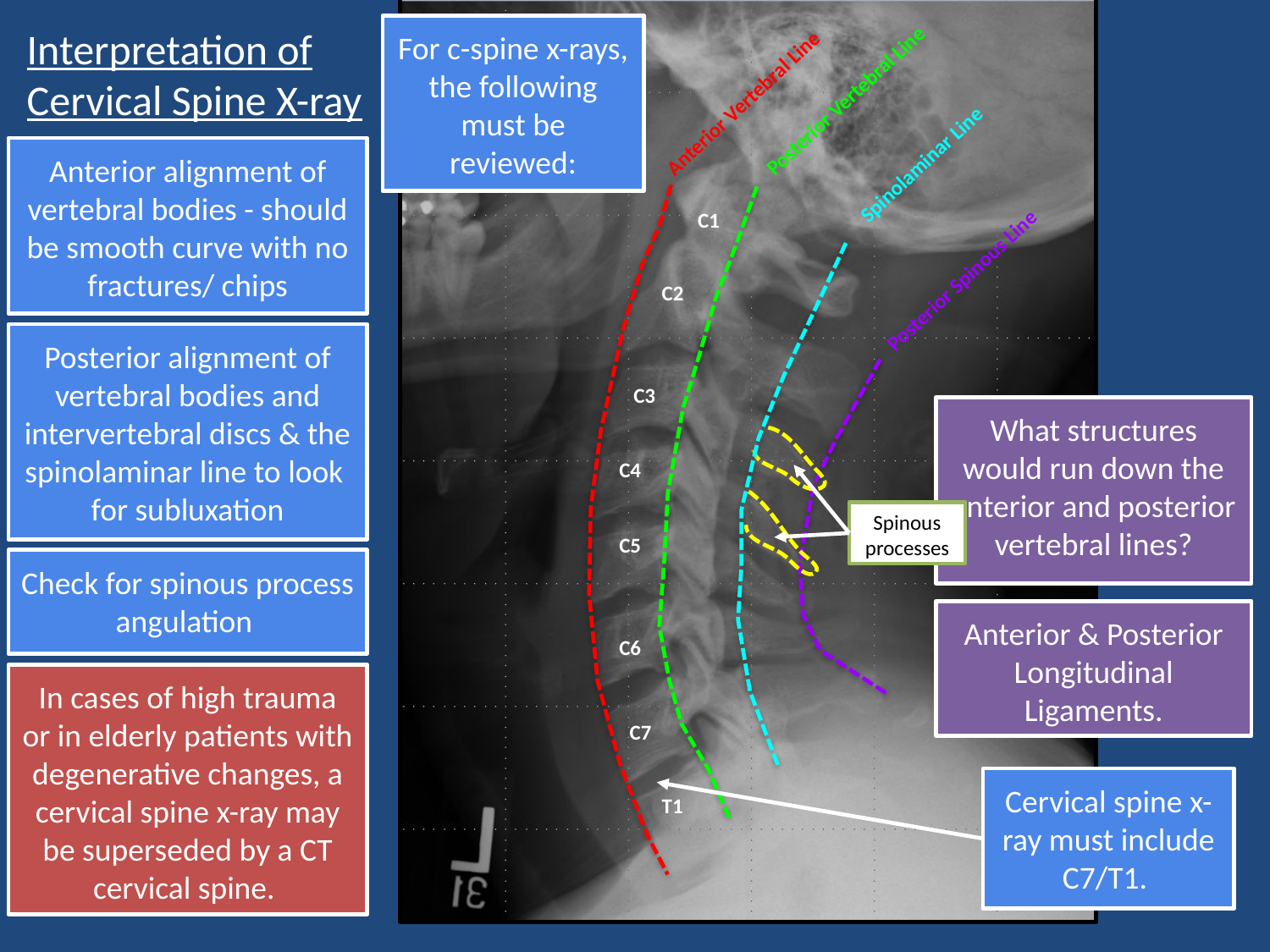

Posterior Vertebral Line
Interpretation of Cervical Spine X-ray
Anterior Vertebral Line
For c-spine x-rays, the following must be reviewed:
Spinolaminar Line
Anterior alignment of vertebral bodies - should be smooth curve with no fractures/ chips
Posterior Spinous Line
C1
C2
Posterior alignment of vertebral bodies and intervertebral discs & the spinolaminar line to look for subluxation
C3
What structures would run down the anterior and posterior vertebral lines?
C4
Spinous processes
C5
Check for spinous process angulation
Anterior & Posterior Longitudinal Ligaments.
C6
In cases of high trauma or in elderly patients with degenerative changes, a cervical spine x-ray may be superseded by a CT cervical spine.
C7
Cervical spine x-ray must include C7/T1.
T1

## Slide 51
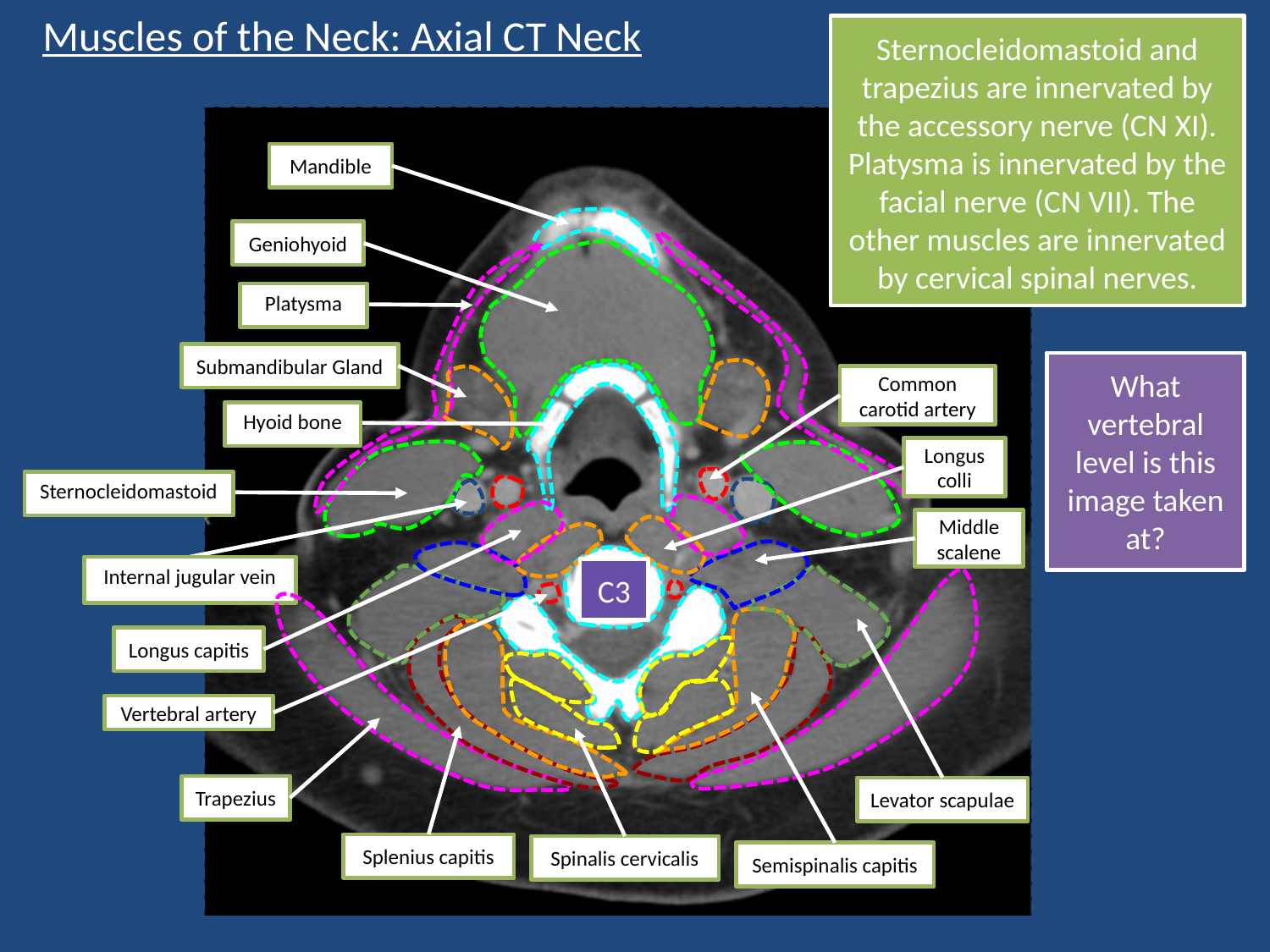

Muscles of the Neck: Axial CT Neck
Sternocleidomastoid and trapezius are innervated by the accessory nerve (CN XI). Platysma is innervated by the facial nerve (CN VII). The other muscles are innervated by cervical spinal nerves.
Mandible
Geniohyoid
Platysma
Submandibular Gland
What vertebral level is this image taken at?
Common carotid artery
Hyoid bone
Longus colli
Sternocleidomastoid
Internal jugular vein
Middle scalene
Longus capitis
C3
Vertebral artery
Levator scapulae
Semispinalis capitis
Trapezius
Splenius capitis
Spinalis cervicalis

## Slide 52
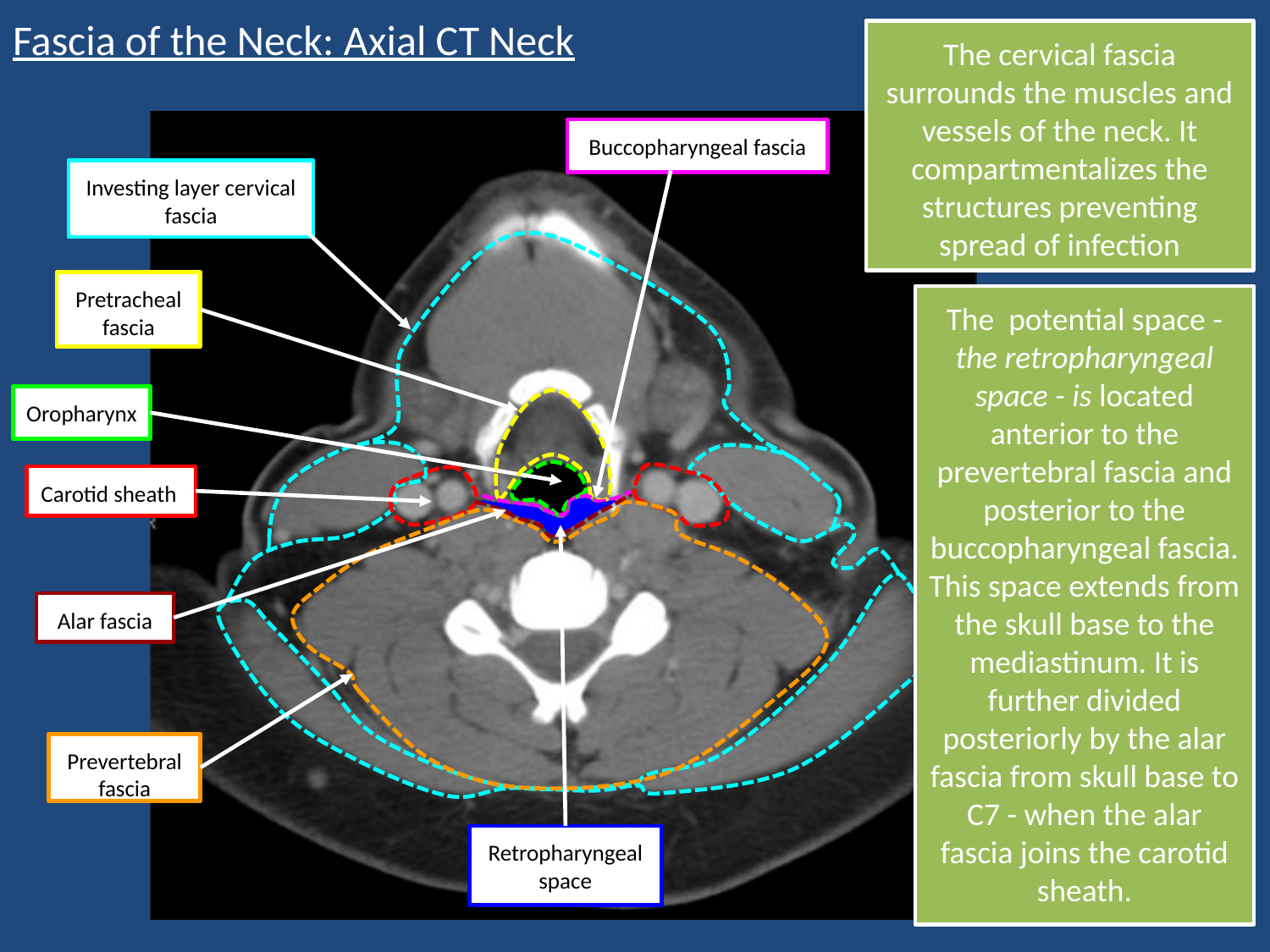

Fascia of the Neck: Axial CT Neck
The cervical fascia surrounds the muscles and vessels of the neck. It compartmentalizes the structures preventing spread of infection
Buccopharyngeal fascia
Investing layer cervical fascia
Pretracheal fascia
The potential space - the retropharyngeal space - is located anterior to the prevertebral fascia and posterior to the buccopharyngeal fascia. This space extends from the skull base to the mediastinum. It is further divided posteriorly by the alar fascia from skull base to C7 - when the alar fascia joins the carotid sheath.
Oropharynx
Carotid sheath
Alar fascia
Prevertebral fascia
Retropharyngeal space

## Slide 53
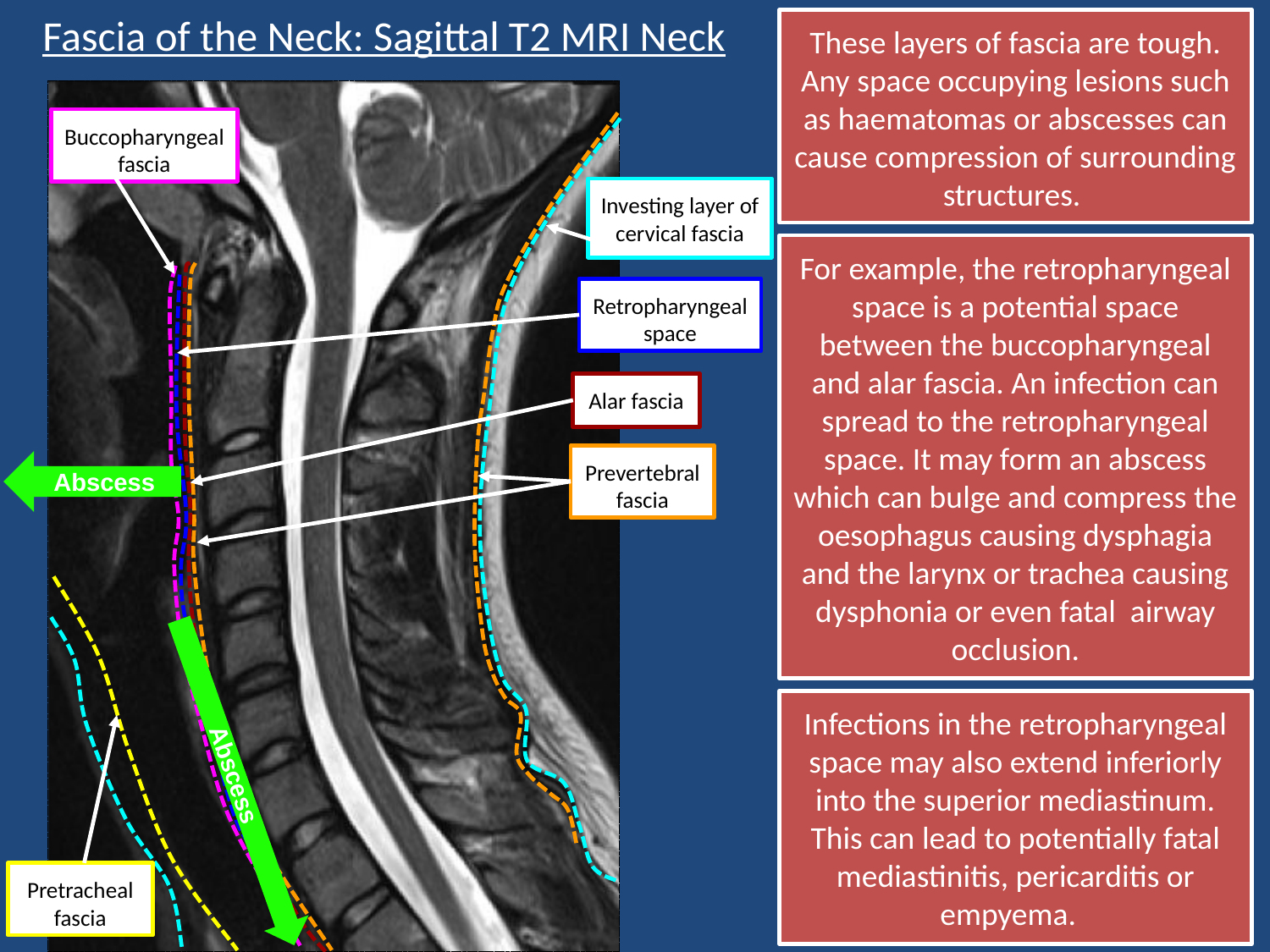

Fascia of the Neck: Sagittal T2 MRI Neck
These layers of fascia are tough. Any space occupying lesions such as haematomas or abscesses can cause compression of surrounding structures.
Buccopharyngeal fascia
Investing layer of cervical fascia
For example, the retropharyngeal space is a potential space between the buccopharyngeal and alar fascia. An infection can spread to the retropharyngeal space. It may form an abscess which can bulge and compress the oesophagus causing dysphagia and the larynx or trachea causing dysphonia or even fatal airway occlusion.
Retropharyngeal space
Alar fascia
Prevertebral fascia
Abscess
Infections in the retropharyngeal space may also extend inferiorly into the superior mediastinum. This can lead to potentially fatal mediastinitis, pericarditis or empyema.
Abscess
Pretracheal fascia

## Slide 54
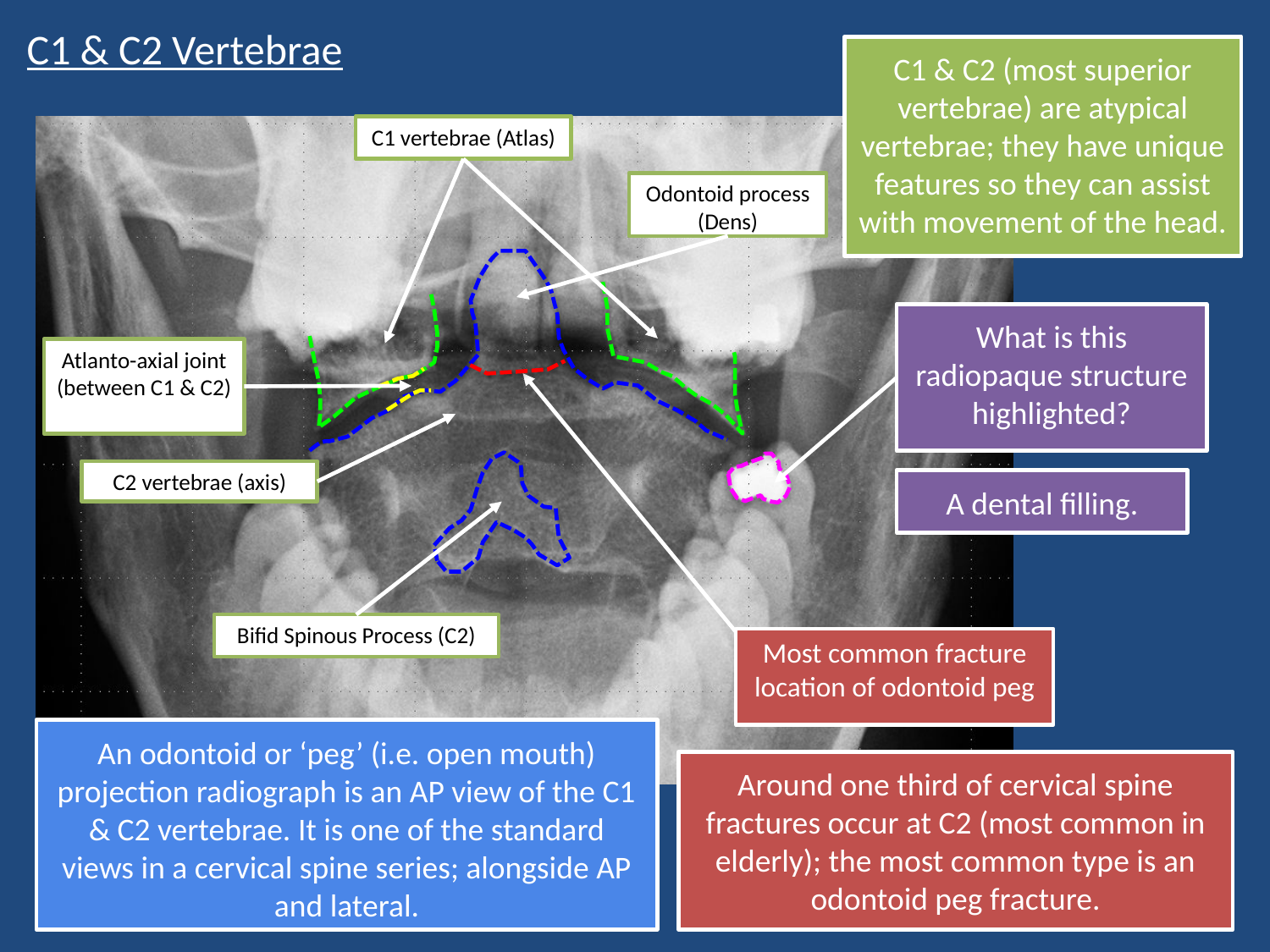

C1 & C2 Vertebrae
C1 & C2 (most superior vertebrae) are atypical vertebrae; they have unique features so they can assist with movement of the head.
C1 vertebrae (Atlas)
Odontoid process (Dens)
What is this radiopaque structure highlighted?
Atlanto-axial joint (between C1 & C2)
Most common fracture location of odontoid peg
C2 vertebrae (axis)
A dental filling.
Bifid Spinous Process (C2)
An odontoid or ‘peg’ (i.e. open mouth) projection radiograph is an AP view of the C1 & C2 vertebrae. It is one of the standard views in a cervical spine series; alongside AP and lateral.
Around one third of cervical spine fractures occur at C2 (most common in elderly); the most common type is an odontoid peg fracture.

## Slide 55
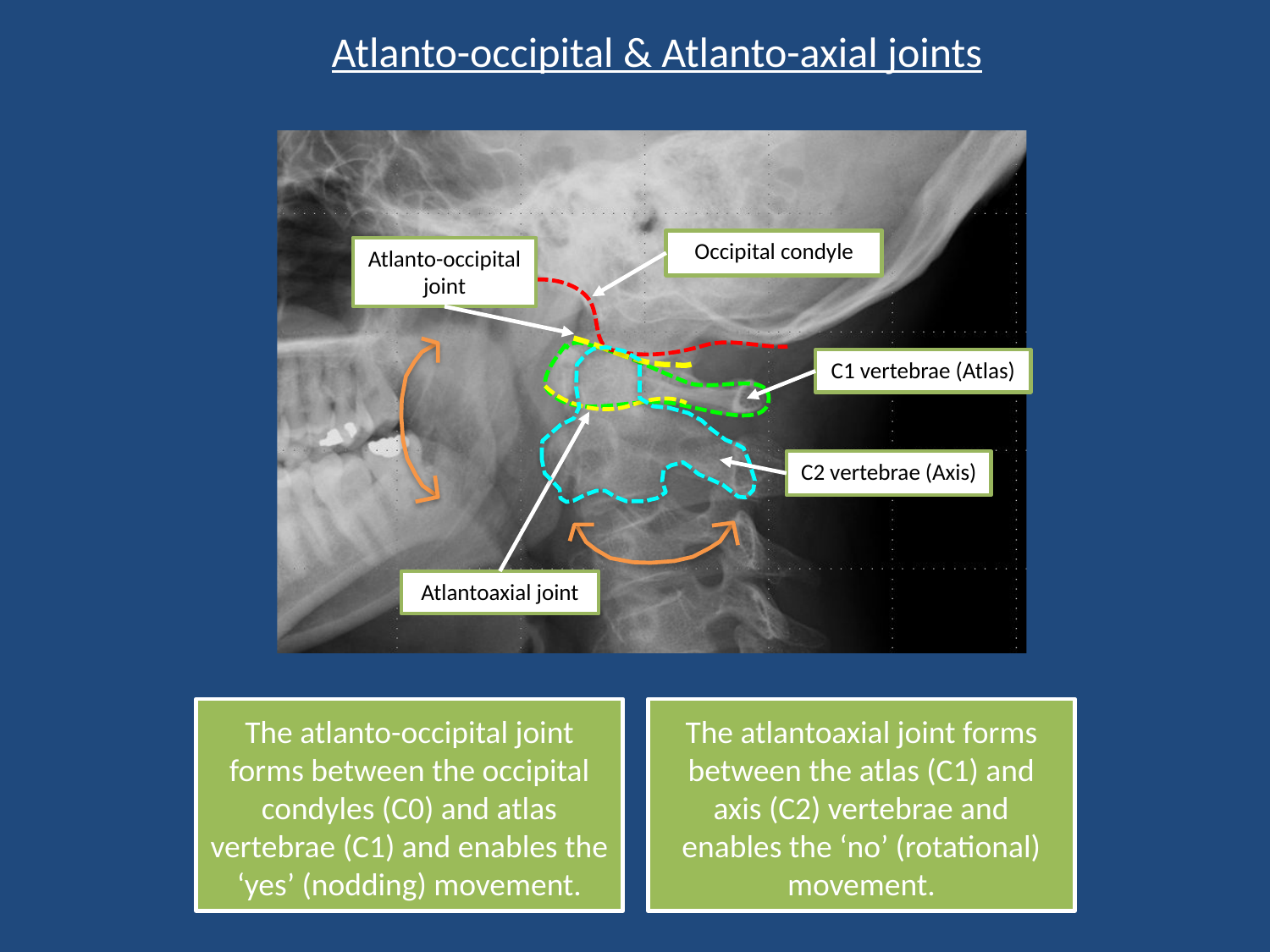

Atlanto-occipital & Atlanto-axial joints
Occipital condyle
Atlanto-occipital joint
C1 vertebrae (Atlas)
Atlantoaxial joint
C2 vertebrae (Axis)
The atlanto-occipital joint forms between the occipital condyles (C0) and atlas vertebrae (C1) and enables the ‘yes’ (nodding) movement.
The atlantoaxial joint forms between the atlas (C1) and axis (C2) vertebrae and enables the ‘no’ (rotational) movement.

## Slide 56
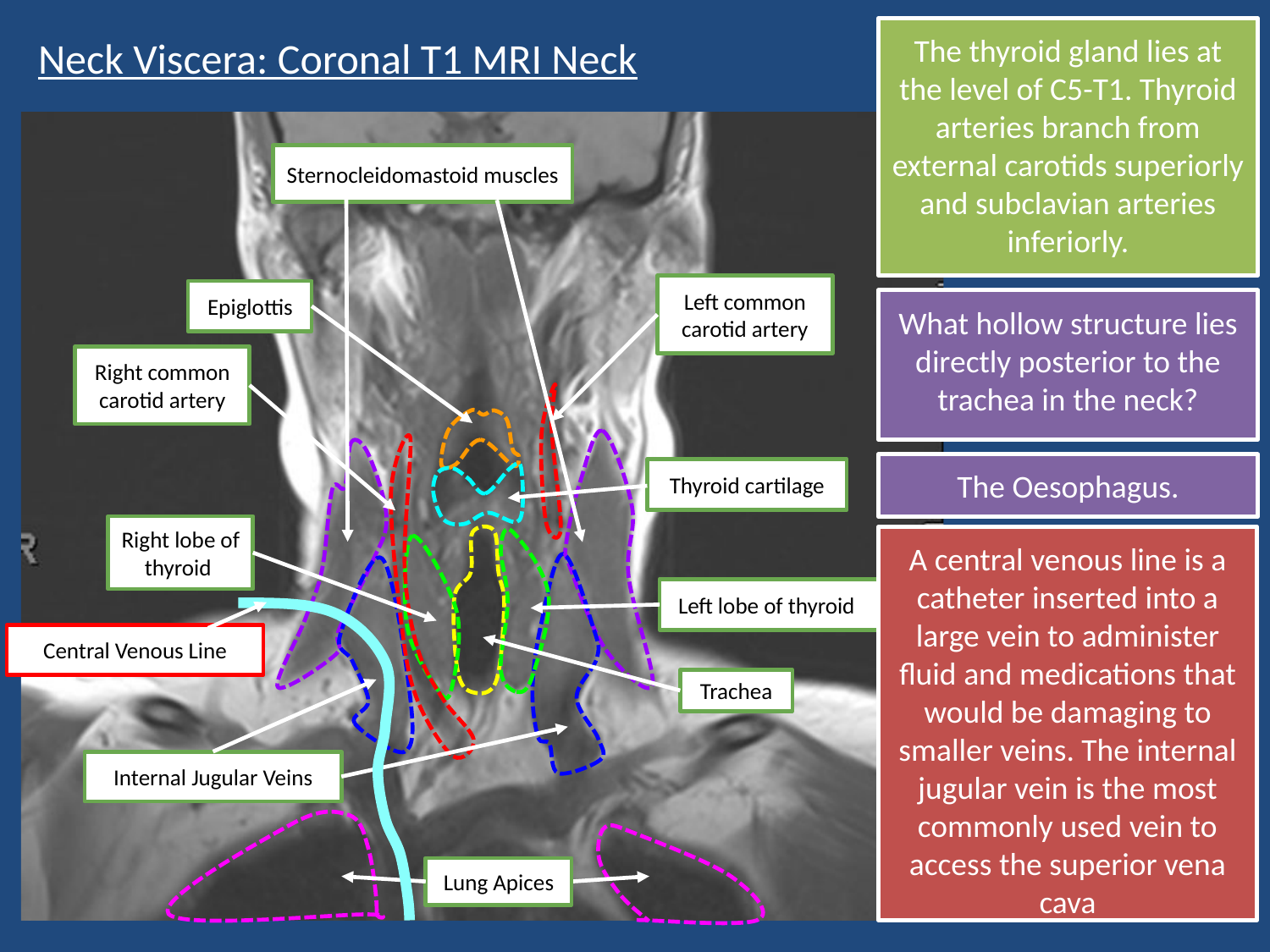

The thyroid gland lies at the level of C5-T1. Thyroid arteries branch from external carotids superiorly and subclavian arteries inferiorly.
Neck Viscera: Coronal T1 MRI Neck
Sternocleidomastoid muscles
Left common carotid artery
Right common carotid artery
Epiglottis
What hollow structure lies directly posterior to the trachea in the neck?
The Oesophagus.
Thyroid cartilage
Right lobe of thyroid
Left lobe of thyroid
A central venous line is a catheter inserted into a large vein to administer fluid and medications that would be damaging to smaller veins. The internal jugular vein is the most commonly used vein to access the superior vena cava
Central Venous Line
Trachea
Internal Jugular Veins
Lung Apices

## Slide 57
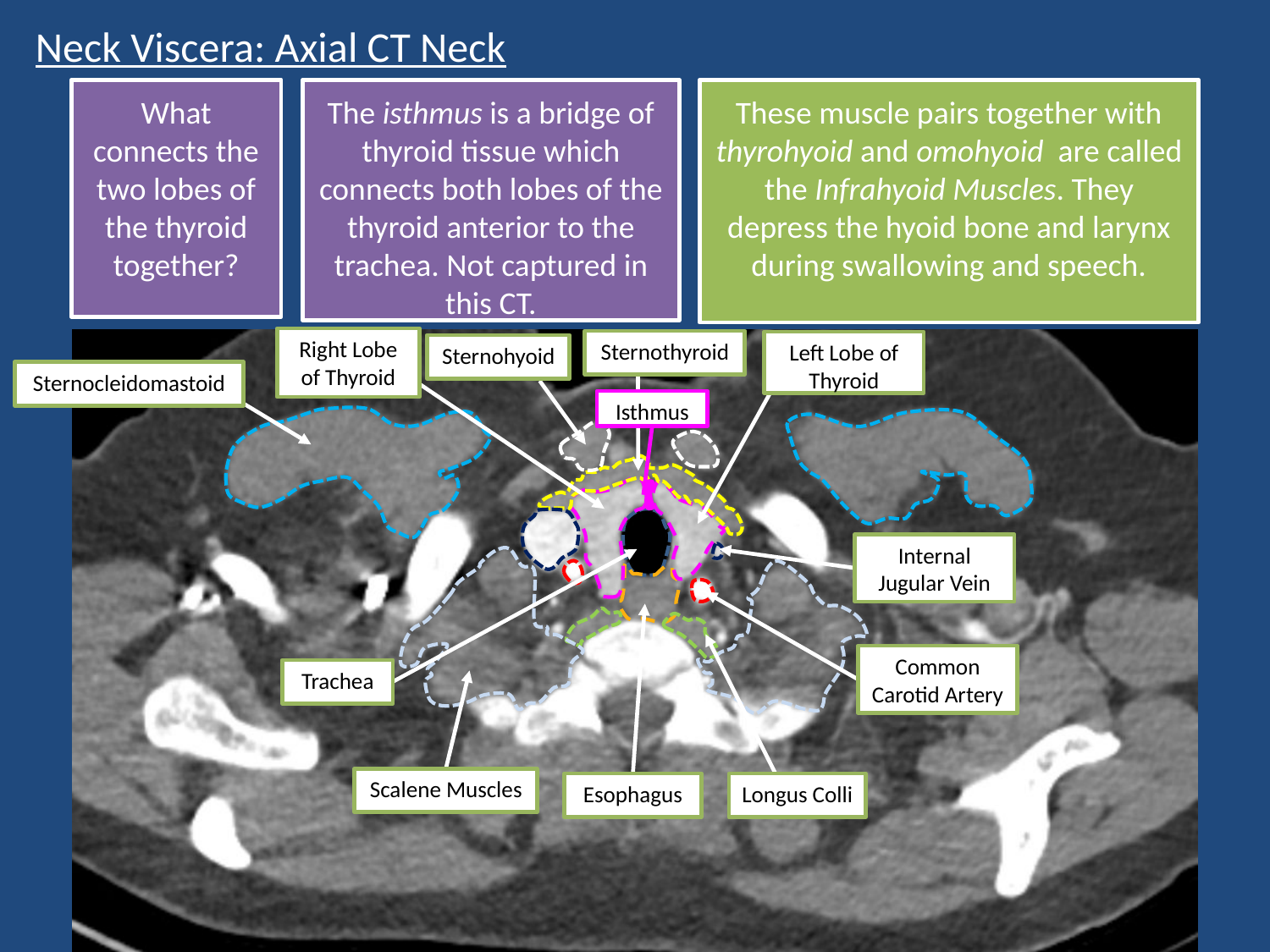

Neck Viscera: Axial CT Neck
The isthmus is a bridge of thyroid tissue which connects both lobes of the thyroid anterior to the trachea. Not captured in this CT.
What connects the two lobes of the thyroid together?
These muscle pairs together with thyrohyoid and omohyoid are called the Infrahyoid Muscles. They depress the hyoid bone and larynx during swallowing and speech.
Right Lobe of Thyroid
Sternothyroid
Left Lobe of Thyroid
Sternohyoid
Sternocleidomastoid
Isthmus
Internal Jugular Vein
Trachea
Common Carotid Artery
Esophagus
Longus Colli
Scalene Muscles

## Slide 58
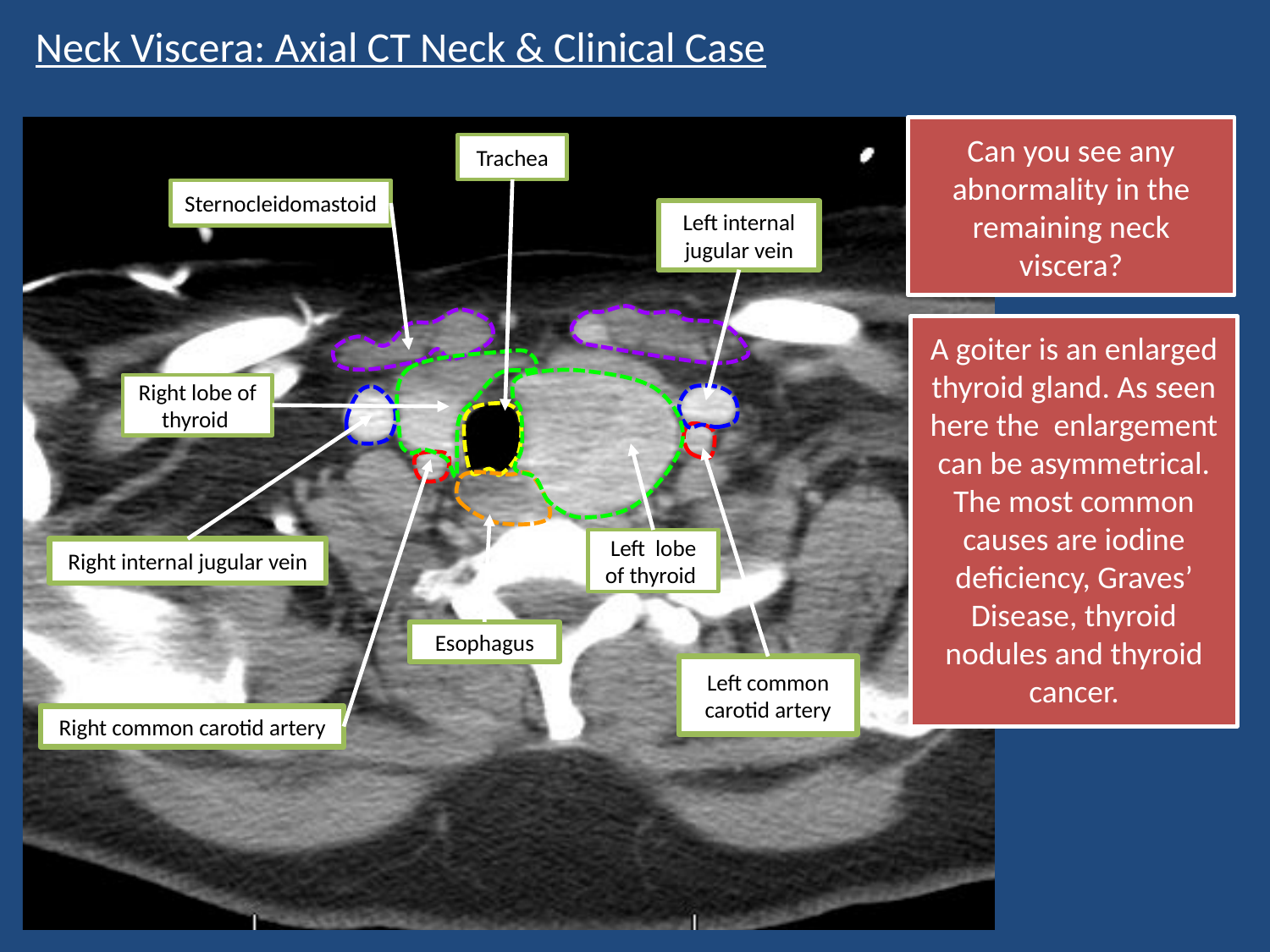

Neck Viscera: Axial CT Neck & Clinical Case
Can you see any abnormality in the remaining neck viscera?
Trachea
Sternocleidomastoid
Left internal jugular vein
A goiter is an enlarged thyroid gland. As seen here the enlargement can be asymmetrical. The most common causes are iodine deficiency, Graves’ Disease, thyroid nodules and thyroid cancer.
Right lobe of thyroid
Left lobe of thyroid
Right internal jugular vein
Left common carotid artery
Right common carotid artery
Esophagus

## Slide 59
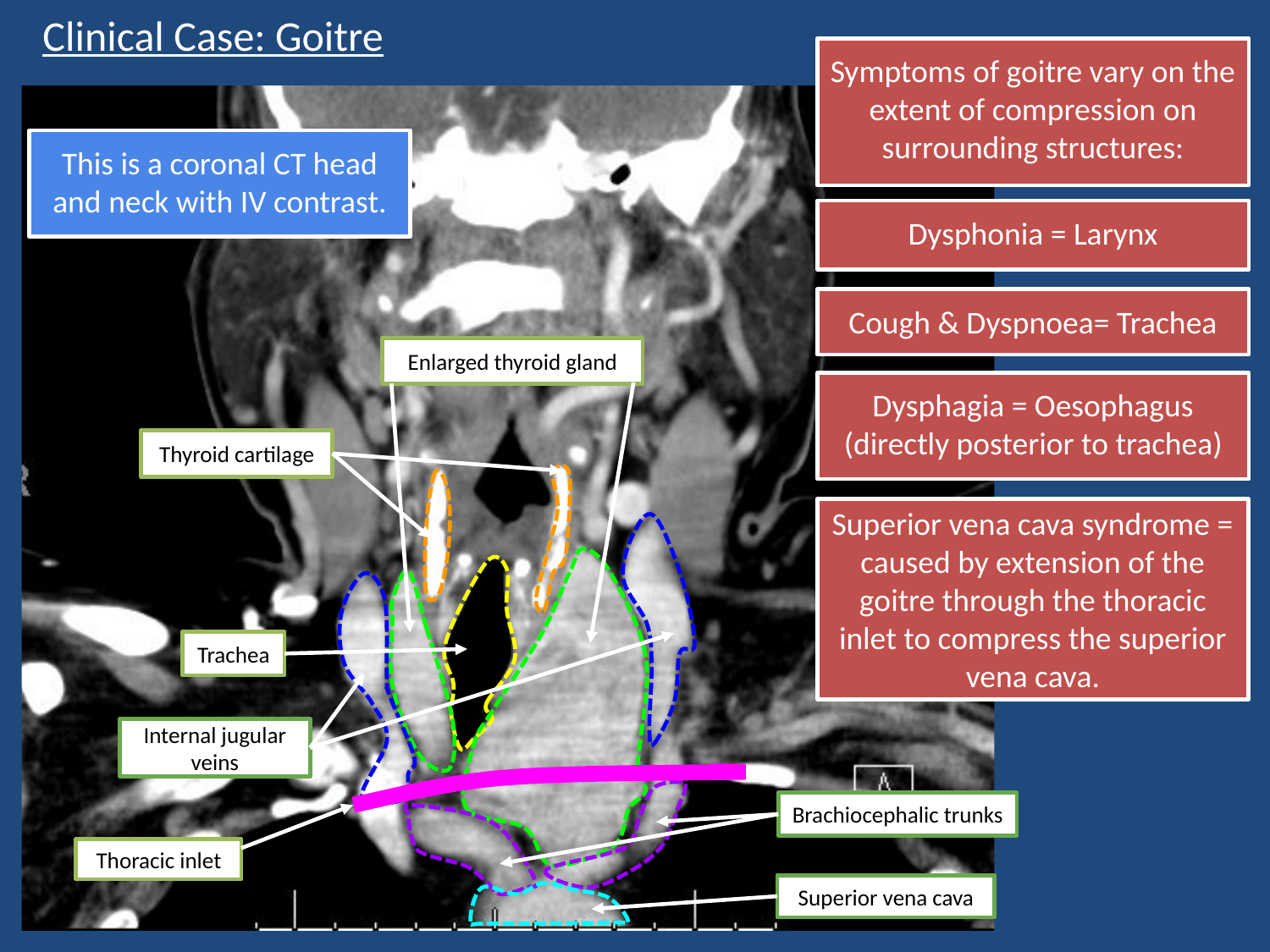

Clinical Case: Goitre
Symptoms of goitre vary on the extent of compression on surrounding structures:
This is a coronal CT head and neck with IV contrast.
Dysphonia = Larynx
Cough & Dyspnoea= Trachea
Enlarged thyroid gland
Dysphagia = Oesophagus (directly posterior to trachea)
Thyroid cartilage
Superior vena cava syndrome = caused by extension of the goitre through the thoracic inlet to compress the superior vena cava.
Trachea
Internal jugular veins
Brachiocephalic trunks
Thoracic inlet
Superior vena cava

## Slide 60
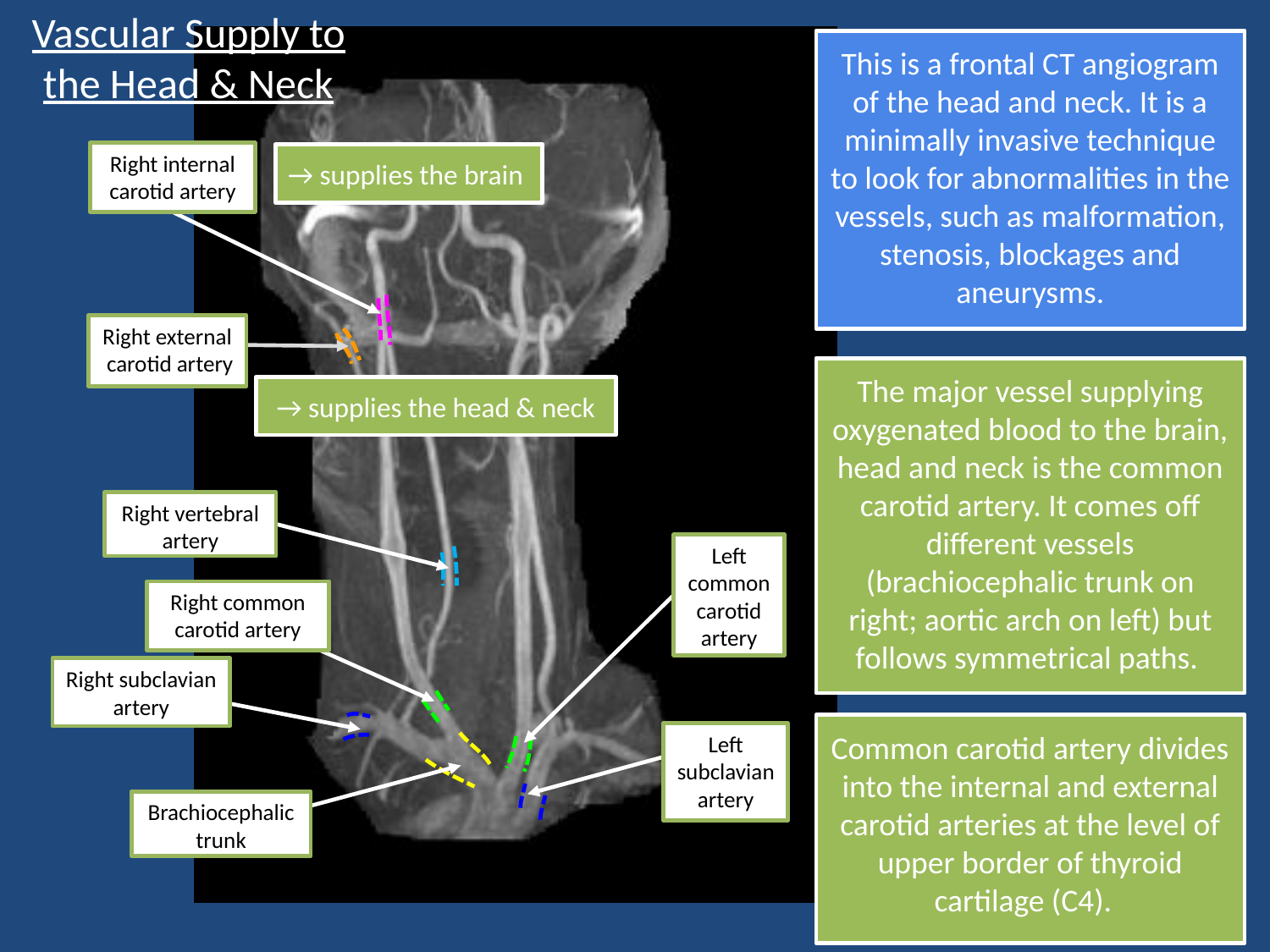

Vascular Supply to the Head & Neck
This is a frontal CT angiogram of the head and neck. It is a minimally invasive technique to look for abnormalities in the vessels, such as malformation, stenosis, blockages and aneurysms.
Right internal carotid artery
→ supplies the brain
Right external carotid artery
The major vessel supplying oxygenated blood to the brain, head and neck is the common carotid artery. It comes off different vessels (brachiocephalic trunk on right; aortic arch on left) but follows symmetrical paths.
→ supplies the head & neck
Right vertebral artery
Left common carotid artery
Right common carotid artery
Right subclavian artery
Common carotid artery divides into the internal and external carotid arteries at the level of upper border of thyroid cartilage (C4).
Left subclavian artery
Brachiocephalic trunk

## Slide 61
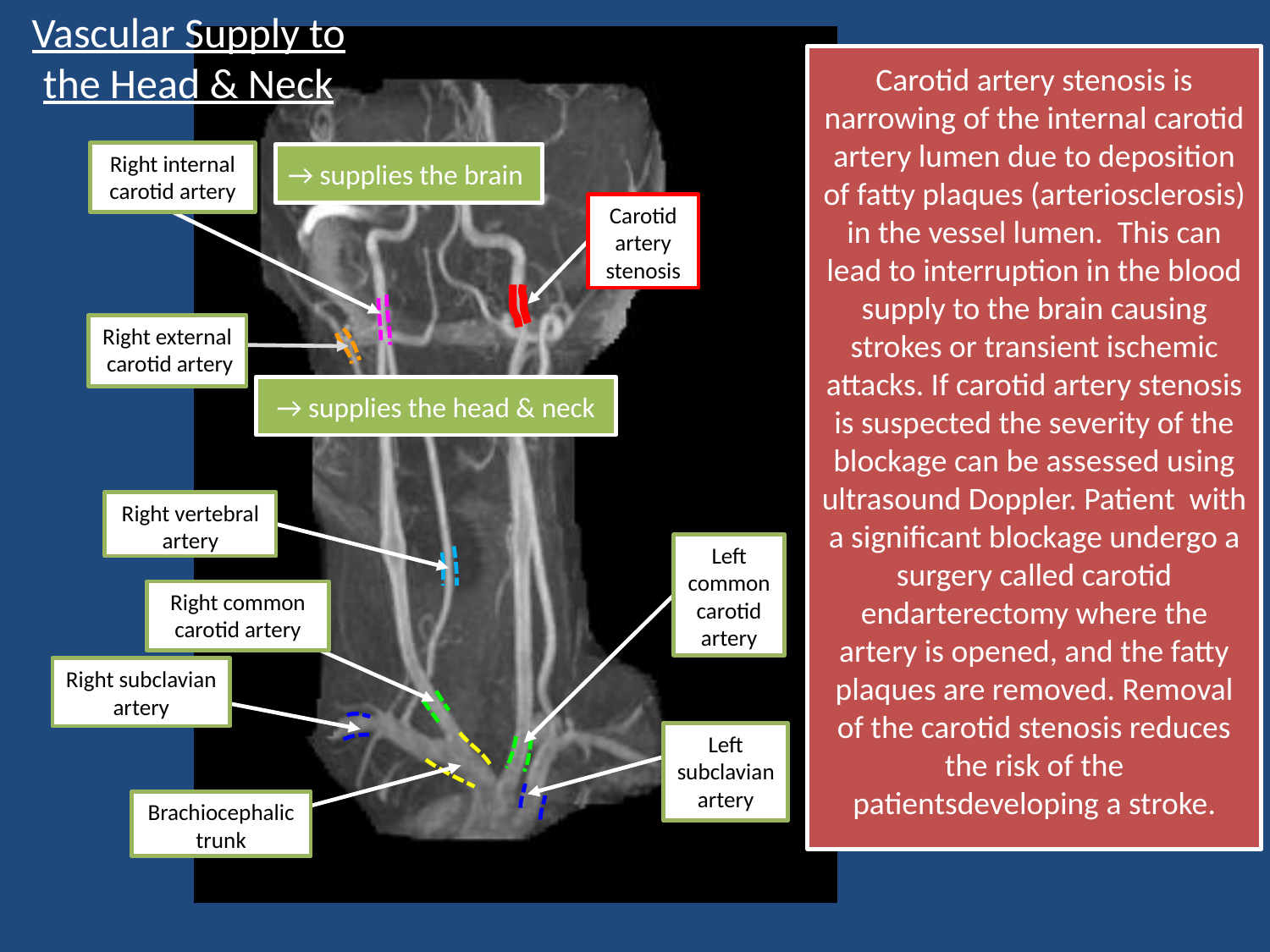

Vascular Supply to the Head & Neck
Carotid artery stenosis is narrowing of the internal carotid artery lumen due to deposition of fatty plaques (arteriosclerosis) in the vessel lumen. This can lead to interruption in the blood supply to the brain causing strokes or transient ischemic attacks. If carotid artery stenosis is suspected the severity of the blockage can be assessed using ultrasound Doppler. Patient with a significant blockage undergo a surgery called carotid endarterectomy where the artery is opened, and the fatty plaques are removed. Removal of the carotid stenosis reduces the risk of the patientsdeveloping a stroke.
Right internal carotid artery
→ supplies the brain
Carotid artery stenosis
Right external carotid artery
→ supplies the head & neck
Right vertebral artery
Left common carotid artery
Right common carotid artery
Right subclavian artery
Left subclavian artery
Brachiocephalic trunk

## Slide 62
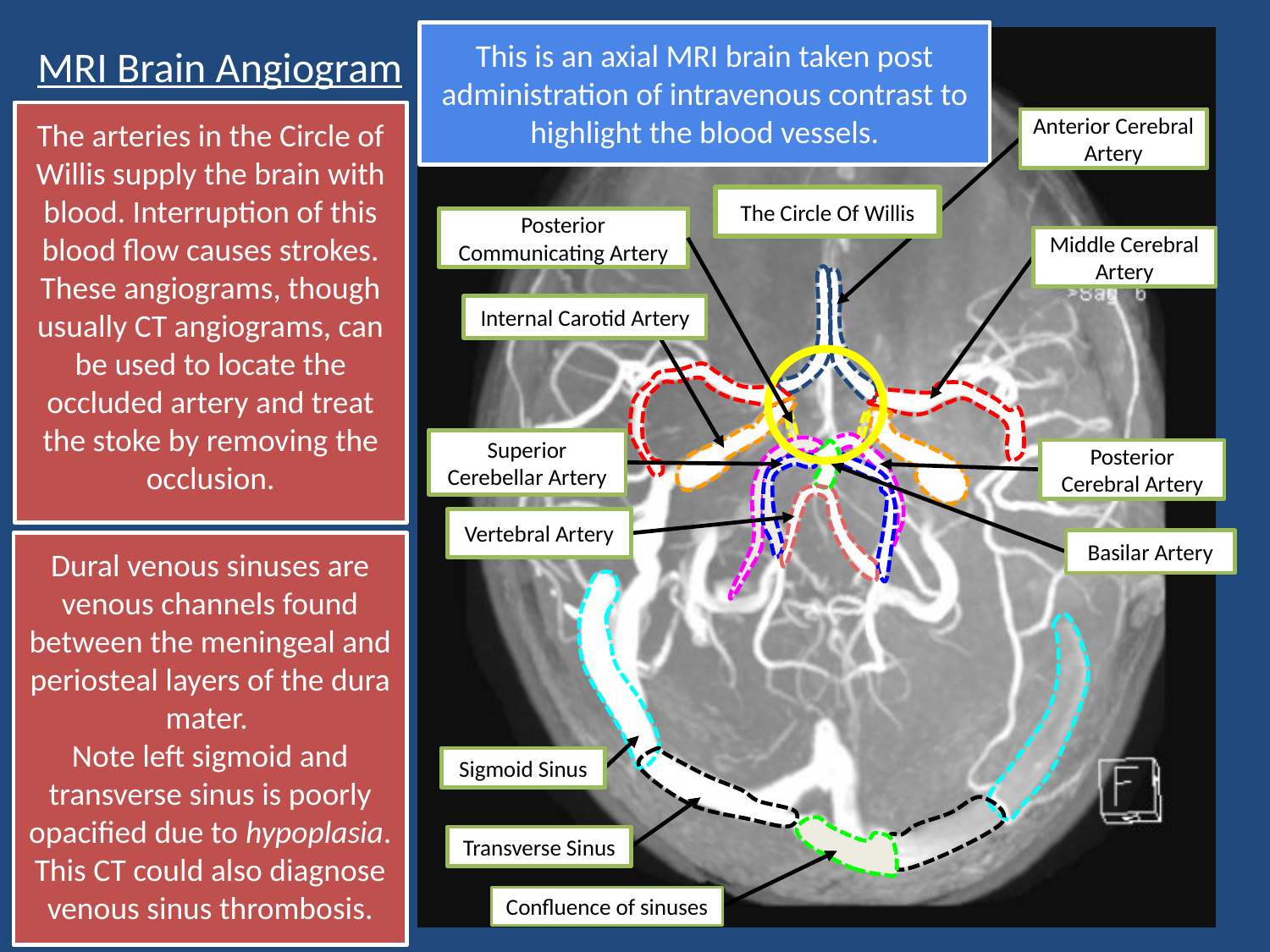

This is an axial MRI brain taken post administration of intravenous contrast to highlight the blood vessels.
MRI Brain Angiogram
The arteries in the Circle of Willis supply the brain with blood. Interruption of this blood flow causes strokes. These angiograms, though usually CT angiograms, can be used to locate the occluded artery and treat the stoke by removing the occlusion.
Anterior Cerebral Artery
The Circle Of Willis
Posterior Communicating Artery
Middle Cerebral Artery
Internal Carotid Artery
Superior Cerebellar Artery
Posterior Cerebral Artery
Basilar Artery
Vertebral Artery
Dural venous sinuses are venous channels found between the meningeal and periosteal layers of the dura mater.
Note left sigmoid and transverse sinus is poorly opacified due to hypoplasia. This CT could also diagnose venous sinus thrombosis.
Sigmoid Sinus
Transverse Sinus
Confluence of sinuses

## Slide 63
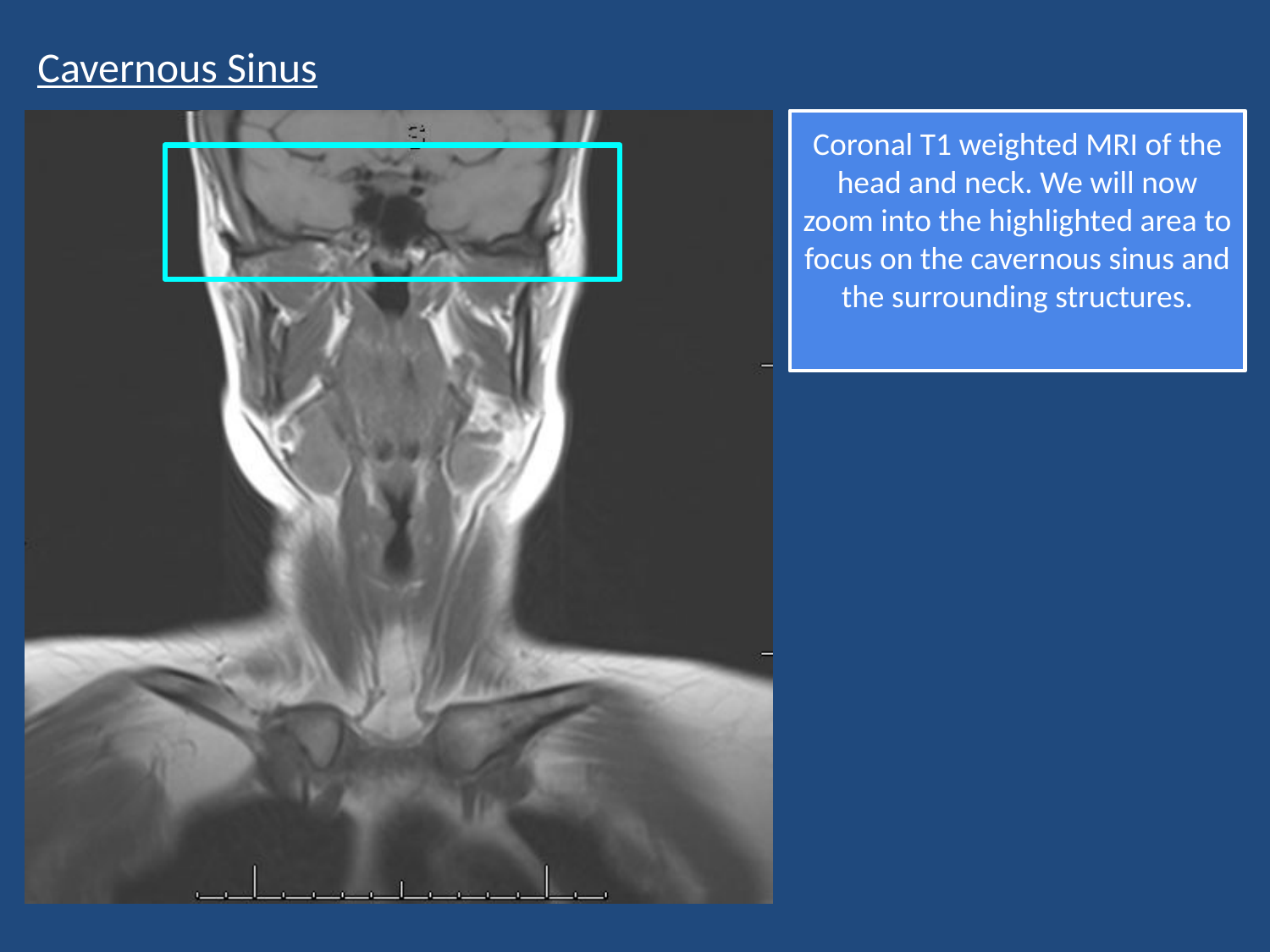

Cavernous Sinus
Coronal T1 weighted MRI of the head and neck. We will now zoom into the highlighted area to focus on the cavernous sinus and the surrounding structures.

## Slide 64
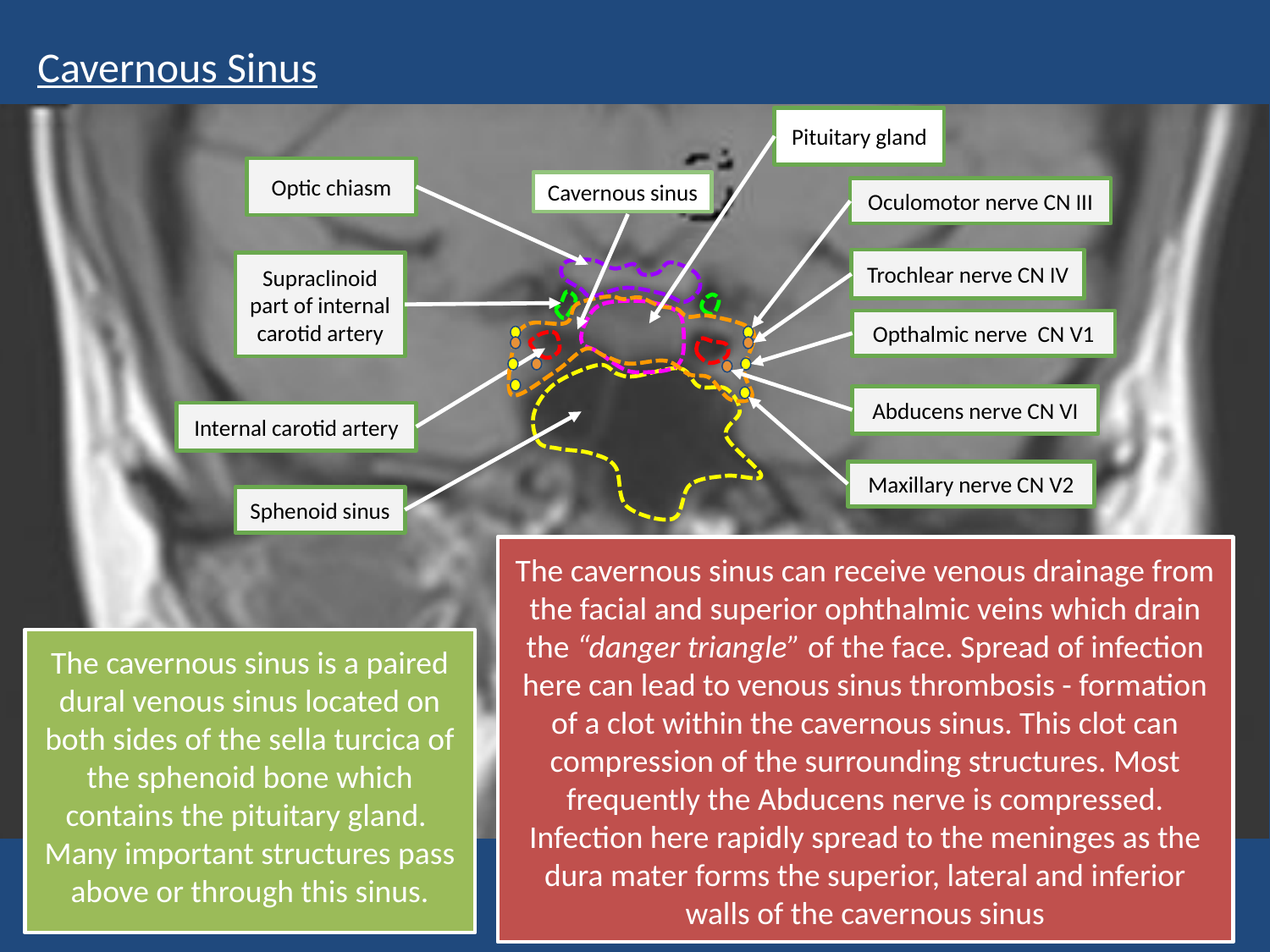

Cavernous Sinus
Pituitary gland
Optic chiasm
Cavernous sinus
Oculomotor nerve CN III
Trochlear nerve CN IV
Supraclinoid part of internal carotid artery
Opthalmic nerve CN V1
Internal carotid artery
Abducens nerve CN VI
Maxillary nerve CN V2
Sphenoid sinus
The cavernous sinus can receive venous drainage from the facial and superior ophthalmic veins which drain the “danger triangle” of the face. Spread of infection here can lead to venous sinus thrombosis - formation of a clot within the cavernous sinus. This clot can compression of the surrounding structures. Most frequently the Abducens nerve is compressed. Infection here rapidly spread to the meninges as the dura mater forms the superior, lateral and inferior walls of the cavernous sinus
The cavernous sinus is a paired dural venous sinus located on both sides of the sella turcica of the sphenoid bone which contains the pituitary gland. Many important structures pass above or through this sinus.

## Slide 65
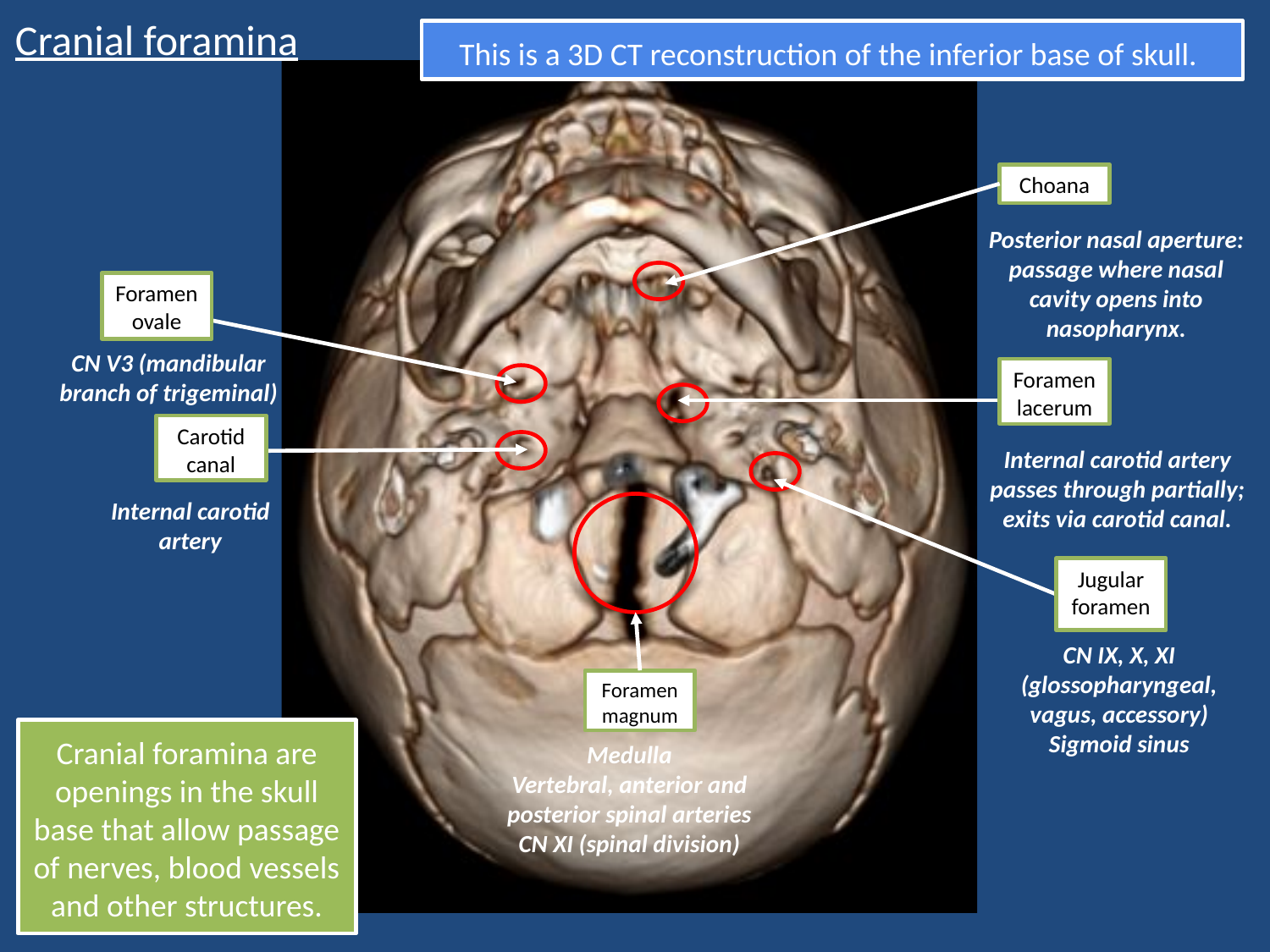

Cranial foramina
This is a 3D CT reconstruction of the inferior base of skull.
Choana
Posterior nasal aperture: passage where nasal cavity opens into nasopharynx.
Foramen ovale
CN V3 (mandibular branch of trigeminal)
Foramen lacerum
Carotid canal
Internal carotid artery passes through partially; exits via carotid canal.
Jugular foramen
Internal carotid artery
Foramen magnum
CN IX, X, XI (glossopharyngeal, vagus, accessory)
Sigmoid sinus
Cranial foramina are openings in the skull base that allow passage of nerves, blood vessels and other structures.
Medulla
Vertebral, anterior and posterior spinal arteries
CN XI (spinal division)

## Slide 66
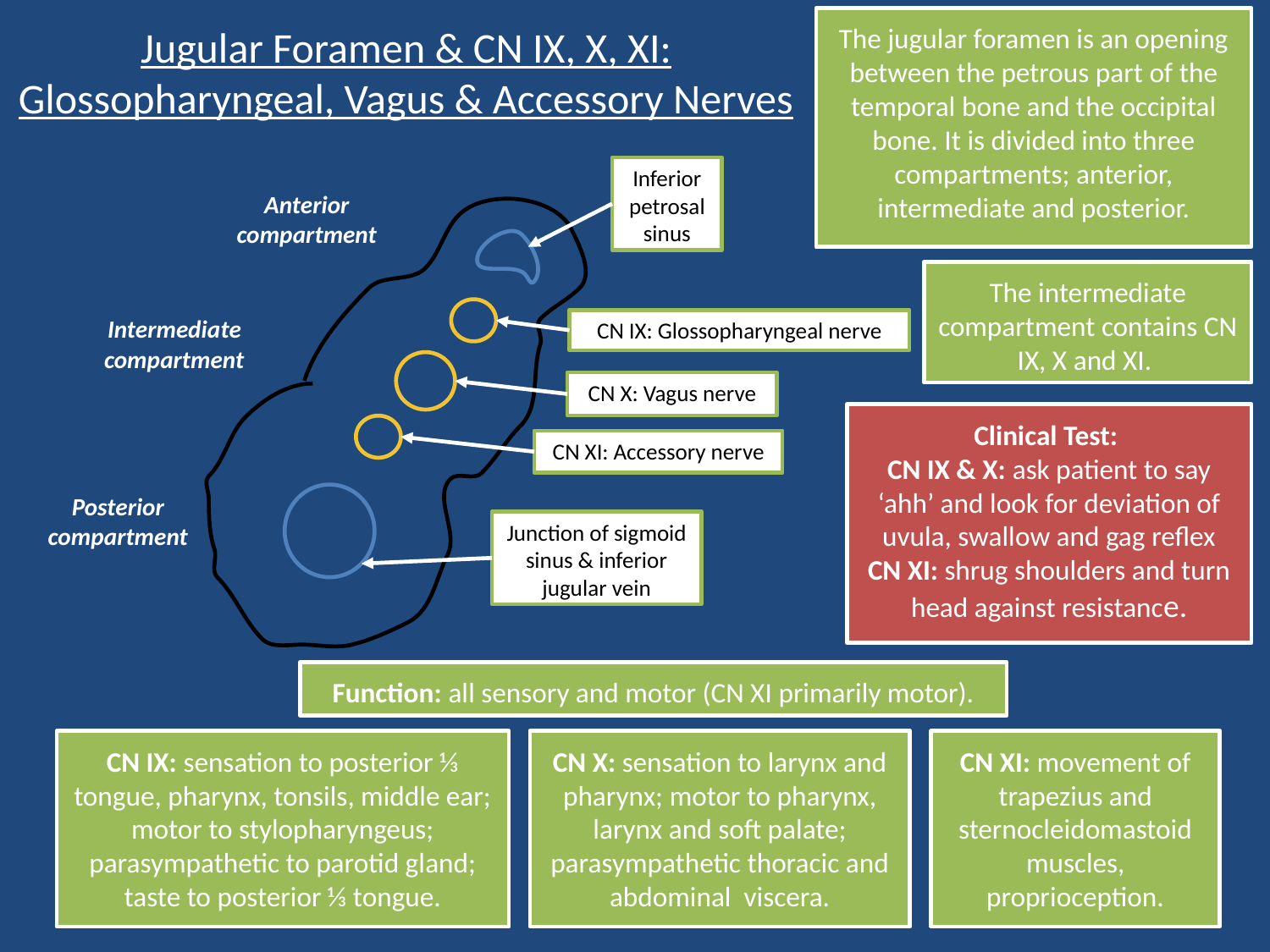

Jugular Foramen & CN IX, X, XI: Glossopharyngeal, Vagus & Accessory Nerves
The jugular foramen is an opening between the petrous part of the temporal bone and the occipital bone. It is divided into three compartments; anterior, intermediate and posterior.
Inferior petrosal sinus
Anterior compartment
The intermediate compartment contains CN IX, X and XI.
Intermediate compartment
CN IX: Glossopharyngeal nerve
CN X: Vagus nerve
Clinical Test:
CN IX & X: ask patient to say ‘ahh’ and look for deviation of uvula, swallow and gag reflex
CN XI: shrug shoulders and turn head against resistance.
CN XI: Accessory nerve
Posterior compartment
Junction of sigmoid sinus & inferior jugular vein
Function: all sensory and motor (CN XI primarily motor).
CN IX: sensation to posterior ⅓ tongue, pharynx, tonsils, middle ear; motor to stylopharyngeus; parasympathetic to parotid gland; taste to posterior ⅓ tongue.
CN X: sensation to larynx and pharynx; motor to pharynx, larynx and soft palate; parasympathetic thoracic and abdominal viscera.
CN XI: movement of trapezius and sternocleidomastoid muscles, proprioception.

## Slide 67
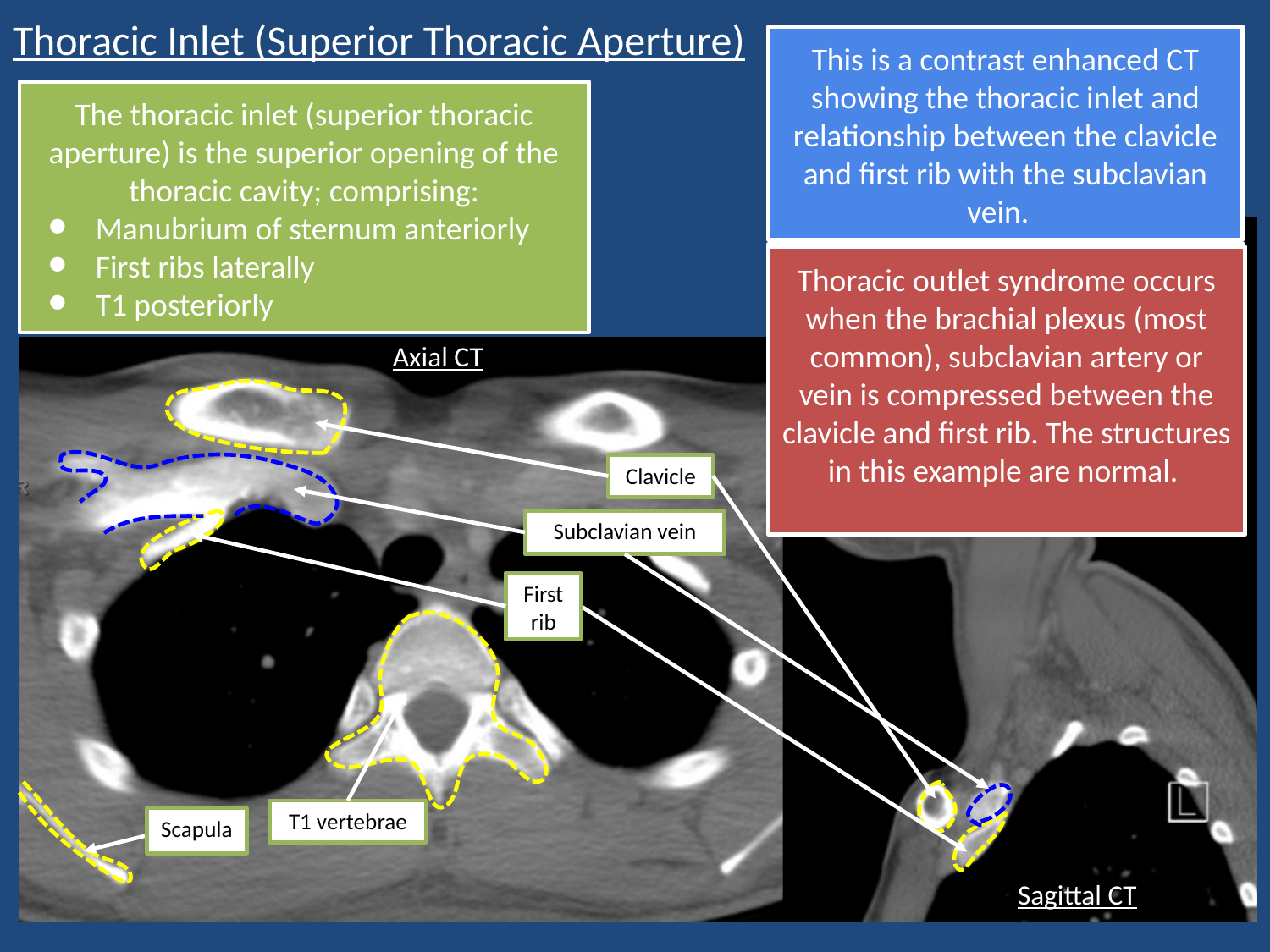

Thoracic Inlet (Superior Thoracic Aperture)
This is a contrast enhanced CT showing the thoracic inlet and relationship between the clavicle and first rib with the subclavian vein.
The thoracic inlet (superior thoracic aperture) is the superior opening of the thoracic cavity; comprising:
Manubrium of sternum anteriorly
First ribs laterally
T1 posteriorly
Multiple viscera, vessels, nerves and muscles pass through the thoracic inlet.
Thoracic outlet syndrome occurs when the brachial plexus (most common), subclavian artery or vein is compressed between the clavicle and first rib. The structures in this example are normal.
Axial CT
Clavicle
Subclavian vein
First rib
T1 vertebrae
Scapula
Sagittal CT

## Slide 68
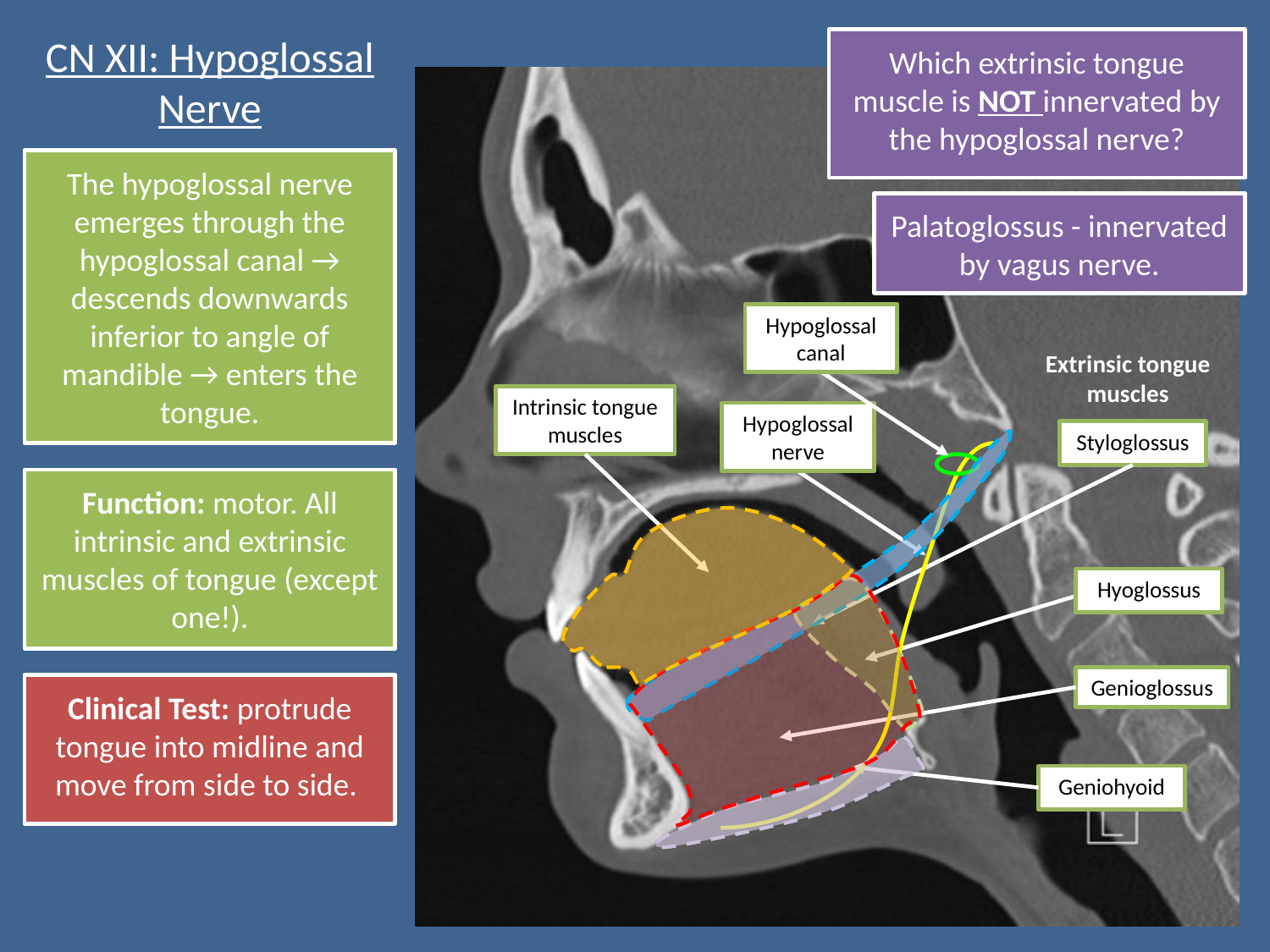

CN XII: Hypoglossal Nerve
Which extrinsic tongue muscle is NOT innervated by the hypoglossal nerve?
The hypoglossal nerve emerges through the hypoglossal canal → descends downwards inferior to angle of mandible → enters the tongue.
Palatoglossus - innervated by vagus nerve.
Hypoglossal canal
Extrinsic tongue muscles
Intrinsic tongue muscles
Hypoglossal nerve
Styloglossus
Function: motor. All intrinsic and extrinsic muscles of tongue (except one!).
Hyoglossus
Genioglossus
Clinical Test: protrude tongue into midline and move from side to side.
Geniohyoid
