# Supplementary material for: Learning Head and Neck Anatomy Through a Radiological Imaging Platform
Source: MedEdPORTAL. 2022 Mar 10;18:11230. doi: 10.15766/mep_2374-8265.11230 (PMC8907321; doi:10.15766/mep_2374-8265.11230)
Supplement: Supplementary file 1 — Head and Neck Imaging Tutorial.pptxPretest.docxPosttest.docxPretest Answers.docxPosttest Answers.docxHead and Neck Tutorial Survey.docx [file mep_2374-8265.11230-s001.zip › B. Pretest.docx]

All images are from the Penn State Teaching Collection, used with permission.

1. Which bone of the skull contains the sella turcica?
2. Ethmoid bone
3. Temporal bone
4. Palatine bone
5. Maxilla bone
6. Sphenoid bone
7. What is the anatomical point bregma defined as?
8. Area between parietal and occipital bones
9. Intersection between coronal and sagittal sutures
10. Intersection between sagittal and lambdoid sutures
11. Area between parietal and temporal bone
12. Posterior fontanelle in an infant
13. Which cranial nerve travels through and branches in the parotid gland?
14. Temporal nerve
15. Facial nerve
16. Vestibulocochlear nerve
17. Glossopharyngeal nerve
18. Vagus nerve
19. Which muscle of mastication opens the jaw?
20. Lateral pterygoid
21. Medial pterygoid
22. Buccinator
23. Masseter
24. Temporalis

5. Travelling anterior to posterior, structures are arranged anatomically?

1. Thyroid -> platysma -> trachea -> oesophagus -> cervical vertebrae
2. Platysma -> thyroid -> trachea -> oesophagus -> cervical vertebrae
3. Platysma -> thyroid -> oesophagus -> trachea -> cervical vertebrae
4. Thyroid -> trachea -> oesophagus -> cervical vertebrae -> platysma
5. Trachea -> platysma -> thyroid -> oesophagus -> cervical vertebrae

6. The nuchal ligament becomes continuous with which spinal ligament around C7?

1. Anterior spinal ligament
2. Supraspinous ligament
3. Posterior spinal ligament
4. Interspinal ligament
5. Ligamentum flavum

7. A nasal bone fracture is most commonly associated with fracture of which paranasal sinus?

1. Maxillary sinus
2. Ethmoidal sinus
3. Frontal sinus
4. Sphenoidal sinus
5. All sinuses equally at risk

8. Which of the following is **incorrect**  regarding the pharynx and swallowing?

1. Hyoid bone draws the larynx upwards and the epiglottis flattens to allow food into the oesophagus
2. Swallowing is innervated by 5 different cranial nerves
3. The oropharynx extends from soft palate to epiglottis
4. The inferior pharyngeal constrictor is at the posterior aspect of the oral cavity
5. The laryngopharynx continues with the oesophagus inferoposteriorly

9. Which spinal nerve emerges inferior to cervical vertebrae 7?

1. C6
2. C7
3. C8
4. T1
5. No spinal nerve emerges at this level

10. Which nerve branch is responsible for the corneal reflex?

A: Facial nerve

B: Oculomotor nerve

C: Maxillary nerve

D: Ophthalmic nerve

E: Abducens nerve

11: The Olfactory bulbs are found superior to which skull bone?

A: Sphenoid bone

B: Ethmoid Bone

C: Nasal Bone

D: Frontal Bone

E: Lacrimal Bone

12: What is the lateral border of the Thoracic Inlet?

A: Clavicle

B: Manubrium

C: 1st Rib

D: 1st Thoracic Vertebrae

E: Humerus

13: Which nerve would an aneurysm of the internal carotid artery within the cavernous sinus likely compress first?

A: Abducens Nerve

B: Optic Chiasm

C: Ophthalmic Nerve

D: Maxillary Nerve

E: Trochlear Nerve

14: A 32 year old non-smoker male presents with a 2 days history of mouth pain. Examination reveals a 1cm tender round swelling at the base of frenulum of his tongue. What is the most likely diagnosis?

A: Sialolithiasis of the parotid duct

B: Tongue cancer

C: Sialolithiasis of the submandibular duct

D: Injury to frenulum

E: Sublingual gland cancer

15: A 56 year old female with multiple sclerosis presents unable to fully abduct her left eye. Damage to what structure explains this?

A: Right Trochlear Nerve

B: Right Abducens Nerve

C: Right Oculomotor Nerve

D: Left Trochlear Nerve

E: Left Abducens Nerve

16: A 20 year old female footie player presents after a blow to the jaw during a game. You suspect a mandible fracture. Examination reveals paresthesia of her lower lip chin. Damage to what structure explains this finding?

A: Lingual Nerve

B: Chorda tympani

C: Mandibular Nerve

D: Mental Nerve

E: Inferior Alveolar Nerve

17: A 68 year old male presents with right sided facial weakness. He also notes 6 month history of headache and nausea. On examination you note complete right side facial weakness, loss of taste to the anterior ⅔ of the right side of the tongue and sensorineural hearing loss in the right ear. A tumour compressing which of these structure would explain these findings?

A: External acoustic meatus

B: Stylomastoid foramen

C: Petrotympanic fissure

D: Geniculate ganglion

E: Internal acoustic meatus

18: Which part of the vertebrae do the vertebral arteries run through?

A: Transverse process

B: Spinous process

C: Vertebral body

D: Pedicle

E: Lamina

19-22: Name the labelled bony structures in the lateral skull X-ray below.


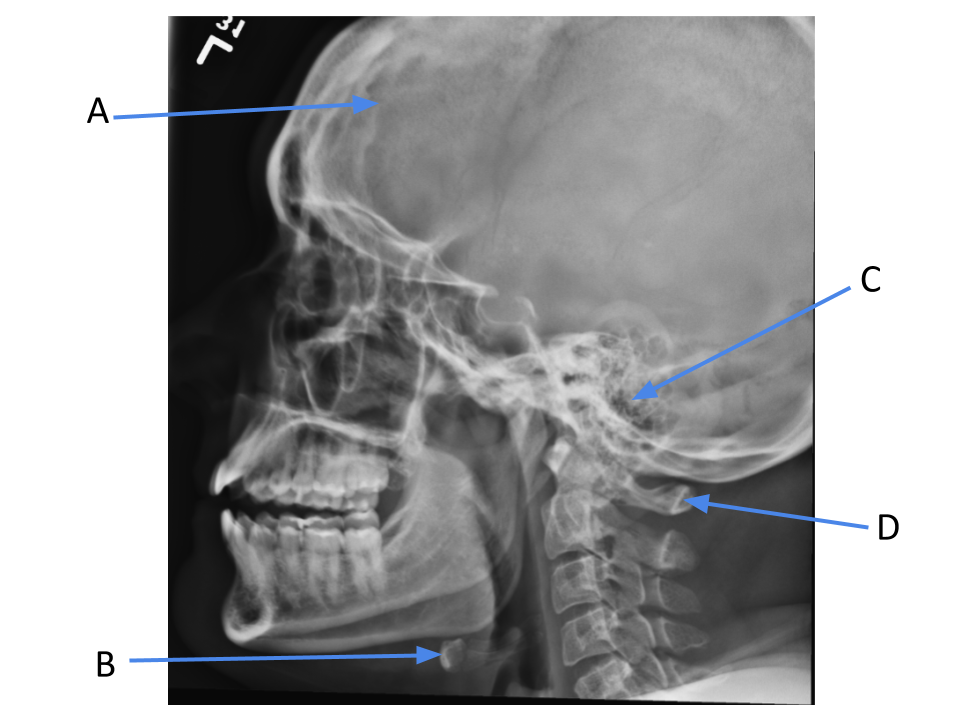


A:_________________________________________________

B:_________________________________________________

C:_________________________________________________

D:_________________________________________________

23-25: Name the labelled structures in the axial MRI Head below


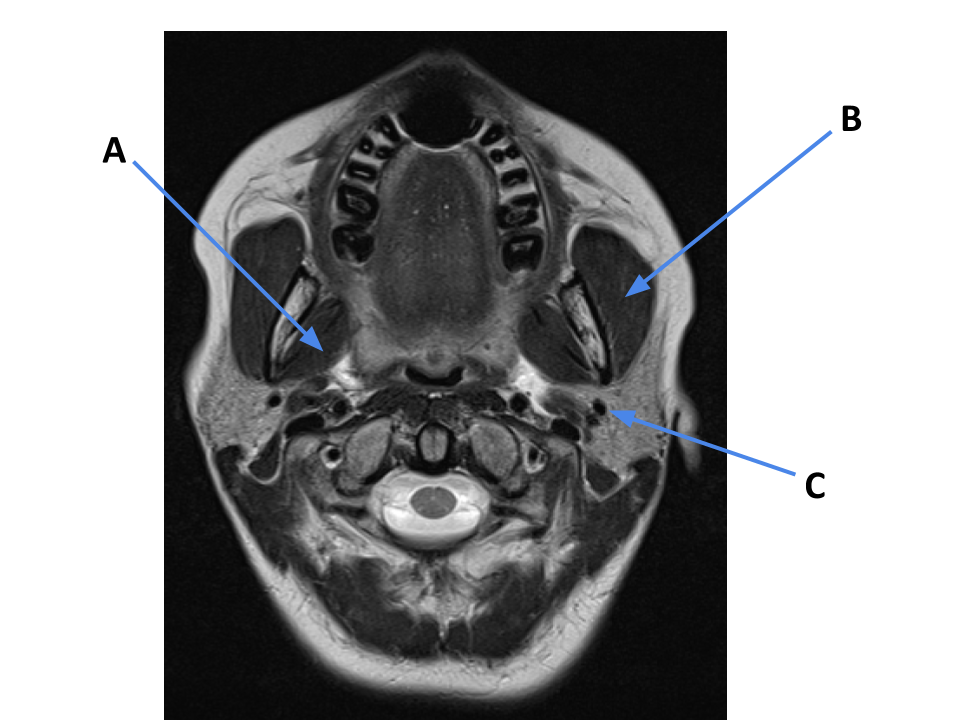


A:_________________________________________________

B:_________________________________________________

C:_________________________________________________

Use the axial MRI below to label the structures described in 26,27 and 28.

26: Label A: The muscle innervated by the abducens nerve

27: Label B: The main visual cortex of the brain

28: Label C: The sinus which drains superior to the superior nasal conchae


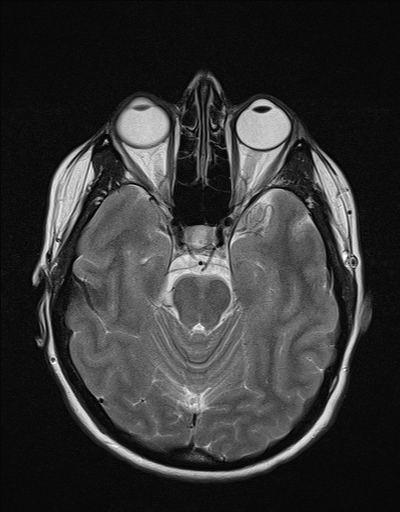


29: In a T1 weighted MRI fluid will appear _____________________________________

30: The structures that pass through the foramen ovale include ______________________

_________________________________________________________________________
