# Supplementary material for: Learning Head and Neck Anatomy Through a Radiological Imaging Platform
Source: MedEdPORTAL. 2022 Mar 10;18:11230. doi: 10.15766/mep_2374-8265.11230 (PMC8907321; doi:10.15766/mep_2374-8265.11230)
Supplement: Supplementary file 1 — Head and Neck Imaging Tutorial.pptxPretest.docxPosttest.docxPretest Answers.docxPosttest Answers.docxHead and Neck Tutorial Survey.docx [file mep_2374-8265.11230-s001.zip › C. Posttest.docx]

All images are from the Penn State Teaching Collection, used with permission.

1: Which bone of the skull contains the cribriform plate?

1. Ethmoid bone
2. Temporal bone
3. Palatine bone
4. Maxilla bone
5. Sphenoid bone

2: What is the anatomical point lambda defined as?

1. Area between parietal and occipital bones
2. Intersection between coronal and sagittal sutures
3. Intersection between sagittal and lambdoid sutures
4. Area between parietal and temporal bone
5. Posterior fontanelle in an infant

3: Spinal branches which cranial nerve travels through the foramen magnum?

1. Hypoglossal nerve
2. Facial nerve
3. Accessory nerve
4. Glossopharyngeal nerve
5. Vagus nerve

4: Which muscle of mastication **does not** close the jaw?

1. Lateral pterygoid
2. Medial pterygoid
3. Buccinator
4. Masseter
5. Temporalis

5: Travelling anterior to posterior, structures are arranged anatomically?

1. Sublingual gland -> symphysis of mandible -> submandibular gland -> parotid gland
2. Symphysis of mandible -> sublingual gland -> parotid gland -> submandibular gland
3. Symphysis of mandible -> sublingual gland -> submandibular gland -> parotid gland
4. Sublingual gland -> symphysis of mandible -> parotid gland -> submandibular gland
5. Submandibular gland -> symphysis of mandible -> sublingual gland -> parotid gland

6: Which ligament runs directly posterior to the spinal cord?

1. Supraspinous ligament
2. Posterior longitudinal ligament
3. Anterior longitudinal ligament
4. Ligamentum flavum
5. Interspinous ligament

7: An inferior orbital floor fracture is likely to lead to blood into which sinus?

1. Ethmoidal sinus
2. Maxillary sinus
3. Frontal sinus
4. Sphenoidal sinus
5. None of the above

8: Which of the following is **incorrect**  regarding the pharynx and swallowing?

1. Sensory and motor aspects of the cranial nerves contribute to swallowing
2. The laryngopharynx is where the passageway diverges into respiratory and digestive systems
3. The nasopharynx extends from the nasal cavity to epiglottis
4. The epiglottis prevents aspiration by covering the airway
5. The superior pharyngeal constrictor is at the posterior aspect of the oral cavity

9: Which spinal nerve emerges superior to cervical vertebrae 1?

1. C1
2. C2
3. C3
4. T1
5. No spinal nerve emerges at this level

10: Damage to which one of these nerves would lead to disequilibrium ?

A: Accessory nerve

B: Vestibulocochlear nerve

C: Maxillary nerve

D: Ophthalmic nerve

E: Facial nerve

11:The Optic nerve travels through which skull bone?

A: Sphenoid bone

B: Ethmoid Bone

C: Nasal Bone

D: Frontal Bone

E: Lacrimal Bone

12: What is the posterior border of the Thoracic Inlet?

A: C7 Vertebrae

B: Trachea

C: 1st Rib

D: 1st Thoracic Vertebrae

E: Mediastinum

13: What structure is found superior to the cavernous sinus?

A: Abducens Nerve

B: Optic Chiasm

C: Ophthalmic Nerve

D: Maxillary Nerve

E: Trochlear Nerve

14: A 32 year old non-smoker male presents with a 2 days history of mouth pain. Examination reveals a 1cm tender round swelling in the cheek by the 2nd maxillary molar. What is the most likely diagnosis?

A: Sialolithiasis of the parotid duct

B: Ranula

C: Sialolithiasis of the submandibular duct

D: Parotid tumour

E: Sublingual gland cancer

15: A 56 year old female with multiple sclerosis presents with difficulty descending stairs. She is unable to rotate her right eye inferomedially. Damage to what structure explains this?

A: Right Trochlear Nerve

B: Right Abducens Nerve

C: Right Oculomotor Nerve

D: Left Trochlear Nerve

E: Left Abducens Nerve

16: A 20 year old male rugby player presents after receiving a blow the jaw during a game. You suspect a mandible fracture. Examination reveals paresthesia of the floor of his mouth, his lower lip and chin. Damage to what structure explains this finding?

A: Lingual Nerve

B: Chorda tympani

C: Mandibular Nerve

D: Mental Nerve

E: Inferior Alveolar Nerve

17: A 68 year old male presents with right sided facial weakness. He also notes 6 month history of headache and nausea. On examination you note complete right side facial weakness but with taste to the anterior ⅔ of the tongue preserved and a normal hearing in both ears . A tumour compressing which of these structure would explain these findings?

A: External acoustic meatus

B: Stylomastoid foramen

C: Petrotympanic fissure

D: Geniculate ganglion

E: Internal acoustic meatus

18: What part of the vertebrae do the spinal nerves emerge from?

A: Intervertebral foramen

B: Vertebral foramen

C: Transverse foramen

D: Anterior to vertebral body

E: Lateral to the spinous process

19-22: Name the labelled structures in the lateral skull X-ray below.


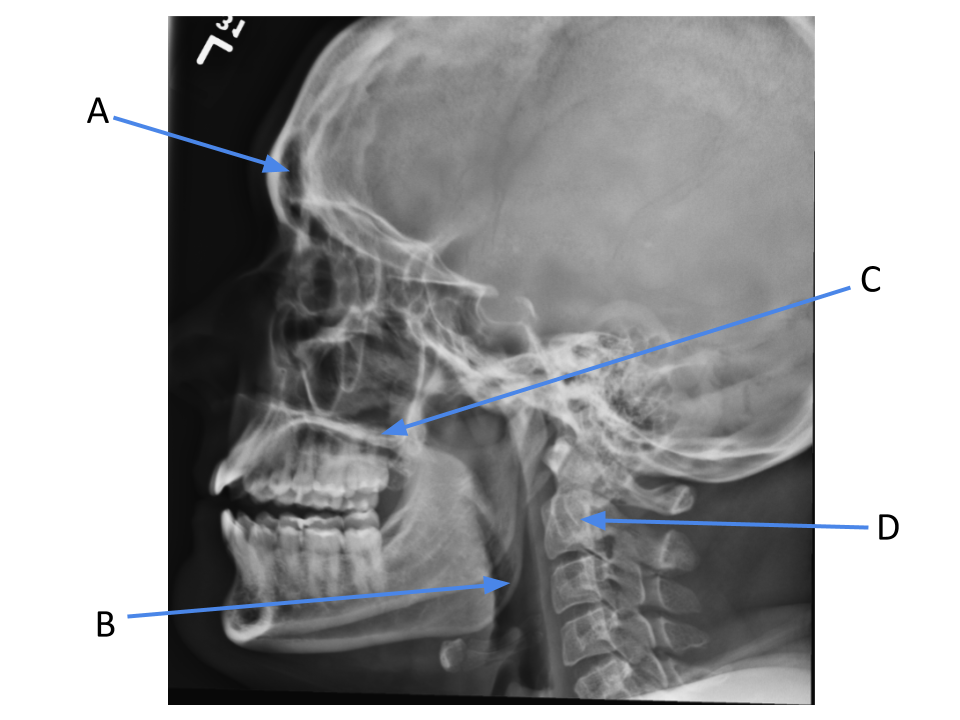


A:_________________________________________________

B:_________________________________________________

C:_________________________________________________

D:_________________________________________________

23-25: Name the labelled structures in the axial MRI Head below


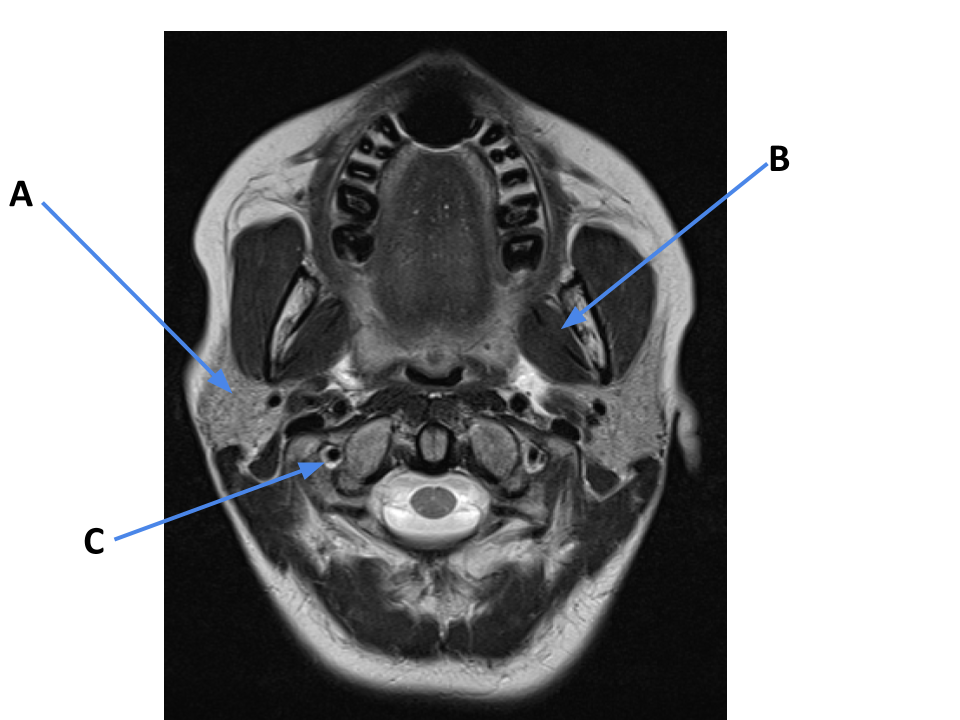


A:_________________________________________________

B:_________________________________________________

C:_________________________________________________

Use the axial MRI below to label the structures described in 26,27 and 28.

26: Label A: A muscle innervated by the oculomotor nerve

27: Label B: The area corresponding to the blindspot on examination

28: Label C: The sinus which partly drains into the middle nasal meatus


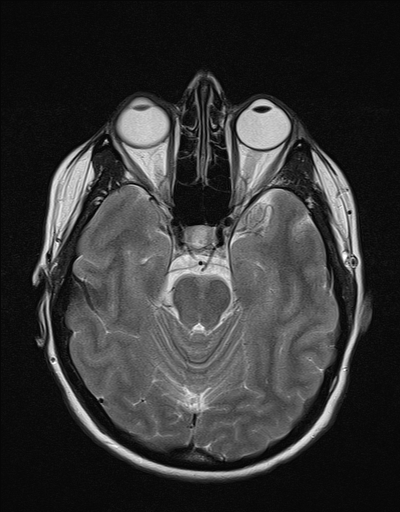


29: In a T2 weighted MRI fluid will appear as _________________________________

30: The structures passing through the jugular foramen include __________________

____________________________________________________________________
