# Supplementary material for: Learning Head and Neck Anatomy Through a Radiological Imaging Platform
Source: MedEdPORTAL. 2022 Mar 10;18:11230. doi: 10.15766/mep_2374-8265.11230 (PMC8907321; doi:10.15766/mep_2374-8265.11230)
Supplement: Supplementary file 1 — Head and Neck Imaging Tutorial.pptxPretest.docxPosttest.docxPretest Answers.docxPosttest Answers.docxHead and Neck Tutorial Survey.docx [file mep_2374-8265.11230-s001.zip › D. Pretest Answers.docx]

| **Q** | **Answer** | **Question Type** |
| --- | --- | --- |
| 1 | E: Sphenoid Bone | Direct |
| 2 | B: Intersection between coronal and sagittal suture | Indirect |
| 3 | B: Facial Nerve | Direct |
| 4 | A: Lateral Pterygoid | Direct |
| 5 | B: Platysma -> thyroid -> trachea -> oesophagus -> cervical vertebrae | Indirect |
| 6 | B: Supraspinous ligament | Direct |
| 7 | C: Frontal sinus | Indirect |
| 8 | D= The inferior pharyngeal constrictor is at the posterior aspect of the oral cavity | Indirect |
| 9 | C: C8 | Indirect |
| 10 | D=Ophthalmic nerve | Indirect |
| 11 | B: Ethmoid Bone | Direct |
| 12 | C: 1st Rib | Direct |
| 13 | A: Abducens Nerve | Indirect |
| 14 | C: Sialolithiasis of the submandibular duct | Indirect |
| 15 | E: Left Abducens nerve | Direct |
| 16 | D: Mental Nerve | Direct |
| 17 | E: Internal acoustic meatus | Indirect |
| 18 | A: Transverse process | Direct |
| 19 | A: Frontal Bone | Direct |
| 20 | B: Hyoid bone | Direct |
| 21 | C: Mastoid Air Cells | Direct |
| 22 | D: Spinous process of C1 Vertebrae (Atlas) | Direct |
| 23 | A: Medial Pterygoid muscle | Direct |
| 24 | B: Masseter muscle | Direct |
| 25 | C: Retromandibular vein | Direct |
| 26 | A: Lateral Rectus Muscle | Direct |
| 27 | B: Occipital Cortex | Direct |
| 28 | C: Sphenoid sinus | Indirect |
| 29 | Black/Dark | Direct |
| 30 | Any two of mandibular nerve V3, lesser petrosal nerve and accessory meningeal artery | Direct |

Indirect= 10

Direct = 20
