# Supplementary material for: Learning Head and Neck Anatomy Through a Radiological Imaging Platform
Source: MedEdPORTAL. 2022 Mar 10;18:11230. doi: 10.15766/mep_2374-8265.11230 (PMC8907321; doi:10.15766/mep_2374-8265.11230)
Supplement: Supplementary file 1 — Head and Neck Imaging Tutorial.pptxPretest.docxPosttest.docxPretest Answers.docxPosttest Answers.docxHead and Neck Tutorial Survey.docx [file mep_2374-8265.11230-s001.zip › E. Posttest Answers.docx]

| **Q** | **Answer** | **Question Type** |
| --- | --- | --- |
| 1 | A: Ethmoid bone | Direct |
| 2 | C: Intersection between sagittal and lambdoid sutures | Indirect |
| 3 | C: Accessory nerve | Indirect |
| 4 | A: Lateral Pterygoid | Direct |
| 5 | C: Symphysis of mandible -> sublingual gland -> submandibular gland -> parotid gland | Indirect |
| 6 | D: Ligamentum flavum | Indirect |
| 7 | B: Maxillary sinus | Indirect |
| 8 | C: The nasopharynx extends from the nasal cavity to epiglottis | Direct |
| 9 | A: C1 | Direct |
| 10 | B: Vestibulocochlear | Direct |
| 11 | A: Sphenoid Bone | Direct |
| 12 | D: 1st Thoracic Vertebrae | Direct |
| 13 | B: Optic Chiasm | Indirect |
| 14 | A: Sialolithiasis of the parotid duct | Indirect |
| 15 | A: Right Trochlear Nerve | Direct |
| 16 | E: Inferior Alveolar Nerve | Indirect |
| 17 | B: Stylomastoid foramen | Direct |
| 18 | A: Intervertebral foramen | Direct |
| 19 | A: Frontal Sinus | Direct |
| 20 | B: Angle of Mandible | Direct |
| 21 | C: Hard palate of maxilla | Direct |
| 22 | D: Vertebral Body of C2 (Axis) | Direct |
| 23 | A: Parotid Gland | Direct |
| 24 | B: Lateral Pterygoid Muscle | Direct |
| 25 | C: Vertebral Artery | Direct |
| 26 | A: Medial Rectus Muscle | Direct |
| 27 | B: Optic nerve entering eye or blind spot | Indirect |
| 28 | C: Ethmoid Air Cell | Direct |
| 29 | White/Bright | Direct |
| 30 | Must include all cranial nerves IX, X, XI (glossopharyngeal, vagus, accessory) plus one of inferior petrosal sinus, meningeal artery branches from occipital and ascending pharyngeal arteries and the sigmoid sinus (becoming the internal jugular vein): | Direct |

Direct: 21

Indirect: 9
