# Supplementary material for: Learning Head and Neck Anatomy Through a Radiological Imaging Platform
Source: MedEdPORTAL. 2022 Mar 10;18:11230. doi: 10.15766/mep_2374-8265.11230 (PMC8907321; doi:10.15766/mep_2374-8265.11230)
Supplement: Supplementary file 1 — Head and Neck Imaging Tutorial.pptxPretest.docxPosttest.docxPretest Answers.docxPosttest Answers.docxHead and Neck Tutorial Survey.docx [file mep_2374-8265.11230-s001.zip › F. Head and Neck Tutorial Survey.docx]

**Head and Neck Tutorial Survey**

Q1. Enter your identification number:

Q2. What age are you?

Q3. What gender do you identify as?

Q4. What specialty(ies) are you currently most interested in pursuing?

Q5. Which one of the following best describes your background?

- Metro
- Rural/Remote
- International

Q6. Overall, what did you think of the tutorial? “1” being excellent and “5” being not good at all.

- 1: Excellent
- 2: Somewhat good
- 3: Moderately good
- 4: Slightly good
- 5: Not good at all

Q7. How effective do you feel this tutorial was at helping you revise head and neck anatomy? “1” being extremely effective and “5” being not effective at all.

- 1: Extremely effective
- 2: Very effective
- 3: Moderately effective
- 4: Slightly effective
- 5: Not effective at all

Q8. How confident do you feel interpreting head and neck medical imaging now having used the tutorial? “1” being extremely significantly more and “5” being “no difference”.

- 1: Significantly More
- 2: Moderately more
- 3: Somewhat more
- 4: Slightly more
- 5: No difference

Q9. How prepared do you feel for starting clinical placements, following use of the tutorial?

“1” being significantly more and “5” being “no difference”.

- 1: Significantly More
- 2: Moderately more
- 3: Somewhat more
- 4: Slightly more
- 5: No difference

Q10. How appropriate was the amount of time allotted for review of the tutorial?

- 1: I needed more time
- 2: Just the right amount of time
- 3: I needed less time

Q11: In your opinion what was most effective about this tutorial and why?

Q12: How could the tutorial improve?

Q13. Any other feedback you would like to provide the developers?
